# Upregulation of Protein O-GlcNAcylation Levels Promotes Zebrafish Fin Regeneration

**DOI:** 10.1016/j.mcpro.2025.100936

**Published:** 2025-03-04

**Authors:** Liyuan Jia, Hanxue Zheng, Juantao Feng, Yi Ding, Xiaotian Sun, Yuan Yu, Xue Hao, Junxiang Wang, Xinyu Zhang, Yuanfeng Tian, Fulin Chen, Jihong Cui

**Affiliations:** 1 School of Medicine, Faculty of Life Science & Medicine, Northwest University, Xi’an, PR China; 2 Laboratory of Tissue Engineering, College of Life Science, Faculty of Life Science & Medicine, Northwest University, Xi’an, PR China; 3 Provincial Key Laboratory of Biotechnology of Shaanxi, Xi’an, PR China; 4 Key Laboratory of Resource Biology and Biotechnology in Western China Ministry of Education, Xi’an, PR China; 5 Pediatric Orthopaedic Hospital, Honghui Hospital, Xi’an Jiaotong University, Xi’an, PR China

**Keywords:** zebrafish, fin regeneration, glycosylation, O-GlcNAc, LC-MS/MS

## Abstract

As one of the most important posttranslational modifications, glycosylation participates in various cellular activities in organisms and is closely associated with many pathogeneses. It has been reported that glycosylation affects the liver, spinal cord, and heart tissue regeneration. The zebrafish fin has become a valuable model due to its high regenerative capacity. The molecular mechanism of regeneration has been a hot research topic in the field for a long time. However, studies on the influence of glycosylation during limb regeneration in zebrafish are relatively scarce. We discovered that N-acetylglucosamine (O-GlcNAc) expression, identified by WGA, was elevated during the regeneration of the injured fin in zebrafish using lectin microarray. This phenomenon is due to the upregulation of the expression of OGT enzymes and elevated O-GlcNAcylation levels. To investigate the effects on the fin regeneration when O-GlcNAcylation changes, we used OSMI-1 or alloxan unilateral microinjection to decrease O-GlcNAcylation and observed that it prevented the fin regeneration. Conversely, the O-GlcNAcylation was impressed by a unilateral microinjection of thiamet-G or glucose into the fin, leading to a stimulation of the fin regeneration. To further understand the role of O-GlcNAcylation in fin regeneration, liquid chromatography-tandem mass spectrometry technology was performed to identify O-GlcNAc-glycoproteins. The results demonstrated that the O-GlcNAc glycoproteins, such as thrombospondin 4 and heparan sulfate proteoglycans, were involved in the regulation of zebrafish fin regeneration process and were closely associated with certain biological processes, such as stem cell differentiation, extracellular matrix-receptor interaction pathway, tissue remodeling, and so on. We demonstrated that O-GlcNAc glycoproteins are crucial for zebrafish fin regeneration, during which OGT promotes the process by upregulating the O-GlcNAcylation levels in the zebrafish fin.

## Highlights

• O-GlcNAcylation upregulated in zebrafish fin regeneration.
• OGT enhances fin regeneration via O-GlcNAcylation, whereas OGA suppresses it in zebrafish.
• O-GlcNAc glycoproteins drive regeneration through glycosylation signaling.

## Graphical Abstract

**Figure:**
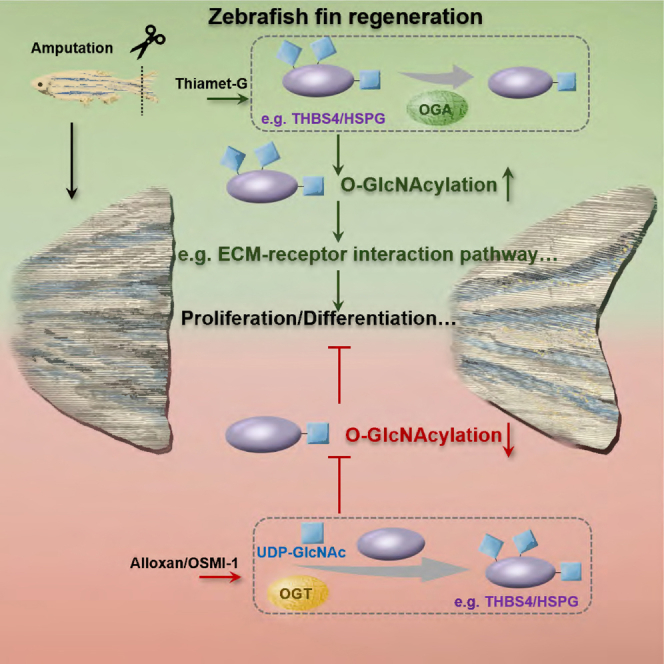

## In Brief

In this study, from the perspectives of glycomics and proteomics, we aimed to construct the protein glycan profile within the regenerated fin of zebrafish and screen for the glycans whose expression altered during this process. Our findings demonstrated that alterations in O-GlcNAc promoted fin regeneration. By identifying the proteins with modified O-GlcNAc, we hope to offer valuable references for future studies concerning glycation modifications in fin regeneration.

Repair and regeneration of severed limbs represent a medical challenge that is formidable. Zebrafish have a strong capacity to regenerate several organs, including the fin, heart, retina, and liver (1). By using zebrafish as a model organism, the study of the regeneration mechanism of its fin will offer an important theoretical basis for the regeneration of amputated limbs in mammals. Once the fin of zebrafish is amputated, it regenerates rapidly (2, 3). This regeneration process generally encompasses three stages. At 0.5 days post amputation (dpa), the apical epidermal cap starts to form in the amputated caudal fin. During this stage, epidermal cells migrate to the wound site to cover it. The earliest molecular marker of this process is β-catenin, whose increased expression promotes cell migration. In addition, *lef1* expression in epidermal cells has been found to promote epithelial–mesenchymal interactions, which are necessary for vertebrate development (4). Within 1 to 2 days post fin amputation, a blastema with division and proliferation abilities forms at the wound site. This blastema formation is the hallmark that distinguishes limb regeneration from embryonic development events. Wnt5 promotes cell proliferation during the blastema formation stage (5). After 4 dpa, fin regeneration shifts from blastema formation to cell differentiation, and a new fin eventually forms. At this stage, *fgf* expression is significantly higher than at the blastema formation stage, and the *msxb* expression is also higher than at the blastema formation stage; moreover, *wnt3a* is only detected at this stage. Fin regeneration is an intricate biological process. Each stage within this process serves distinct functions and is governed by different mechanisms. However, the underlying complex regulatory mechanisms remain incompletely understood (5, 6).

According to earlier research, glycosylation modification is crucial for embryonic growth and organ regeneration (7, 8, 9). N-acetylglucosamine (O-GlcNAc) glycoproteins regulate apoptosis and ectoderm movement during the embryonic development of zebrafish (10). O-mannosylation modification is required in the composition of embryonic development. When O-mannosyltransferases are inhibited, severe embryonic malformations will be induced (9). Both the development of the embryo and the growth of the adult zebrafish depend on the terminal polysialylation of N-glycans on the neural cell adhesion protein. They play a role in the creation of muscular tissue (11), retinal formation (12, 13), axon bundling and commissural axon guidance (14), and neuronal migration. Core fucosylation is necessary for the formation of the axial pattern during embryonic development (15). Furthermore, β-1,4-galactosylation is crucial for establishing the dorsal–ventral axis during embryonic development and facilitating cell migration in the convergent extension movement (16). Ontogeny and cell activation are intimately associated with modifications in sugar chain structure. The physiological activities of cells can occasionally be impacted by the use of exogenous glycosylation inhibitors or glycosidases, suggesting that sugar chains regulate receptor activation responses, cell-cell interactions, and cell migration patterns throughout development. Changes in various kinds of glycosylation accompany the process of tissue regeneration, just as they do throughout embryonic development. Studies on glycosylation and regeneration are currently few, and further research and development are needed in this area.

However, the specific role of glycosylation modification in zebrafish fin regeneration remains unclear. We acquired the glycan expression profile of glycoproteins during zebrafish fin regeneration using lectin microarrays. The results demonstrated that O-GlcNAc expression was elevated during the regeneration process. Then, O-GlcNAc glycosylation-related inhibitors and agonists were injected into the fin via microinjection. It was discovered that O-GlcNAcylation exerted an influence on and was implicated in fin regeneration. Eventually, the types and abundances of O-GlcNAc glycoproteins were identified by means of liquid chromatography-tandem mass spectrometry (LC-MS/MS), and bioinformatics analysis was carried out to explore the protein expression data associated with fin regeneration. This thereby offers novel perspectives for the research into the mechanisms underlying zebrafish fin regeneration.

## Experimental Procedures

### Experimental Design and Statistical Rationale

#### The Study Encompasses Three Phases

1) We constructed the protein glycan profile in the regenerated fin of zebrafish, screened for the glycan whose expression changed during the process. 2) We aimed to investigate the regulatory role of O-GlcNAcylation in fin regeneration through microinjection. 3) We planned to enrich the O-GlcNAcylation proteins for proteomic mass spectrometry identification.

#### Experimental Animal and Fin Amputation

The zebrafish utilized in this study are from the AB-Tubingen strain and were bought from Wuhan National Zebrafish Resource Center. Zebrafish were fed at 26 to 28 °C, with 14 h of regulated light and 10 h of darkness each day. Zebrafish that were mature and had the same ratio of males to females were utilized in this investigation. All animal experimental procedures were approved by the Experimental Animal Management and Ethics Committee of Northwest University (NWU-AWC-20240105Z) and were carried out in accordance with relevant national and international guidelines.

#### Experimental Grouping

Fish were anesthetized with a buffered solution of 0.6 mM tricaine (MS-222, Sigma-Aldrich, Cat# E10521, RRID: AB_2534074) in system water during amputation and imaging. For each replicate, 5 to 10 zebrafish were homogenized together. Tissue samples of regenerating fin were taken 1 mm above the incision plane at 0.5 dpa, 1 dpa, 2 dpa, 4 dpa, and 6 dpa, respectively. The fin fragments amputated immediately were used as control (Ctrl or 0 dpa). The tissue from the amputated fin was cleansed and collected using a 1.5 ml tube for protein or RNA extraction for later use.

#### Data Processing

The lectin microarray comprises 37 types of lectins, along with negative and positive controls. Each spot is replicated three times. After the microarray scanning, the relative fluorescence values undergo median normalization and are subsequently compared with those of the control group.

All peptide-spectrum matches were identified by Proteome Discoverer (https://www.thermofisher.cn/order/catalog/product/CSW0064769?SID=srch-srp-CSW0064769) through probability-based scoring methods (Sequest-HT, with a *p*-value <0.01). Subsequently, they were further validated via label-free quantification (LFQ) of peptide MS/MS identifications combined with MS1-level quantifications, ensuring the accuracy of the assignments. Pooled samples were analyzed to perform data quality control. The datasets were derived from a single shotgun experiment, incorporating one biological replicate and one technical replicate.

### Lectin Microarray

**Fig. 1:**
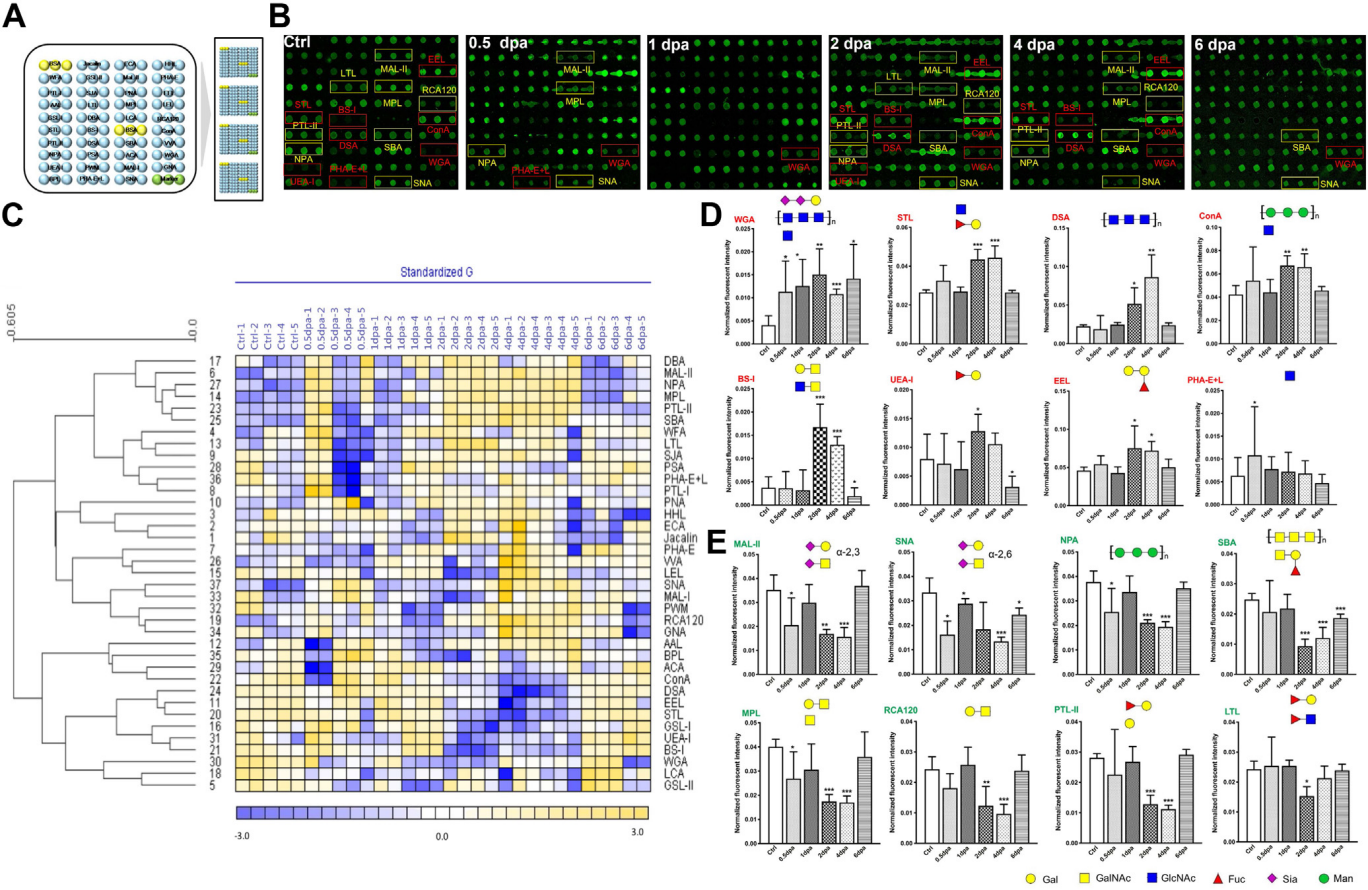
**The different glycopatterns in Ctrl, 0.5 dpa, 1 dpa, 2 dpa, 4dpa, and 6 dpa using lectin microarray.***A*, the layout of the lectin microarrays. Each lectin was spotted in triplicate per block, with quadruplicate blocks on one slide. Cy3-labeled BSA was spotted as a location marker and BSA as a negative control. *B*, the glycopatterns of a Cy3-labeled pooled zebrafish fin tissue protein sample bound to the lectin microarrays. The lectin microarrays revealed significant signal differences between Ctrl, 0.5 dpa, 1 dpa, 2 dpa, 4 dpa, and 6 dpa, marked with *red* frames (upregulation) and *yellow* frames (downregulation). *C*, heat map and hierarchical clustering analysis of the 37 lectins with five biological replicates. Samples were listed in *columns*, and the lectins were listed in *rows*. The color and intensity of each square indicated expression levels relative to other data in the row. *Yellow*, high; *purple*, low; and *white*, medium. 0.001). *D*, significant upregulation among Ctrl, 0.5 dpa, 1 dpa, 2 dpa, 4 dpa, and 6 dpa were analyzed according to Student’s *t* test, respectively. *E*, significant downregulation among Ctrl, 0.5 dpa, 1 dpa, 2 dpa, 4 dpa, and 6 dpa were analyzed according to Student’s *t* test, respectively (∗*p* < 0.05, ∗∗*p* < 0.01, and ∗∗∗*p* ≤ 0.001). The data were the averaged NFI ± SD of five biological replicates. Glc, *blue circle*; GlcNAc, *blue square*; Gal, *yellow circle*; GalNAc, *yellow square*; Fuc, *red triangle*; NeuAc, *purple diamond*; Man, *green circle*. BSA, bovine serum albumin; dpa, days post amputation.

Tissue samples were grinded using a homogenizer, and total protein was isolated using radioimmunoprecipitation assay lysis buffer (Takara). After coloring fin tissue proteins with Cy3 fluorescent dye, the Sephadex G-25 columns purified them (GE HealthCare). Lectin microarrays were developed (17, 18) (Fig. 1*A*, more information about lectin microarray are available at Supplemental Fig. S12). The slides were immobilized, blocked for 1 h with 2% bovine serum albumin in 1 × PBS (0.01 mol/L phosphate buffer, 0.15 mol/L NaCl, pH 7.4), and twice washed with 1 × PBS. Cy3-labeled proteins in 0.6 ml incubation buffer were incubated on the blocked slide for 3 h at room temperature (RT). After incubation, the microarray was washed twice with 1 × PBST (0.2% Tween-20 in 1 × PBS) for 5 min and once with 1 × PBS before drying. Axon Instruments' Genepix 4000B confocal scanner in Foster City, California, scanned microarrays at 70% photomultiplier tube and 100% laser power. For Cy3 detection, Genepix 3.0 software (Original developer: Axon Instruments, now part of Molecular Devices, LLC) evaluated the images at 532 nm.

### Lectin Histochemistry and Immunofluorescence

The paraffin sections of the fin tissue were rehydrated and repaired with 10% sodium citrate antigen. Sections were blocked for 1 h at RT with 5% goat serum in 1 × PBST and coated with 200 ml of primary antibody diluted (Cy5-lectin, O-GlcNAc transferase (OGT), RL-2) in blocking solution overnight at 4 °C in the humid chamber. They were rinsed in 1 × PBS for 1 h at RT and returned to the humid chamber for secondary antibody incubation diluted in blocking solution (it is unnecessary for lectin histochemistry). Slides were washed in 1 × PBST for 1 h at RT and sealed with 30% glycerin (19). The following primary antibodies were used: OGT at 1:200 (Abcam, Cat# ab96718, RRID: AB_10680993), RL-2 at 1:150 (Abcam, Cat# ab2937, RRID: AB_303264). DAPI (Sigma-Aldrich, Cat# D9542, 1:10,000) was applied to label nuclei.

### Lectin Blot and Western Blot

Protein samples were boiled for 5 min at 100 °C with 5 × loading buffer and conducted on a 10% polyacrylamide resolving gel and 3% stacking gel. Proteins in gels were transferred to a polyvinylidene fluoride membrane (Immobilon-P; Millipore Corp) utilizing a wet transfer equipment (Hoefer Scientific) for 1.5 h at 150 mA. Following the transfer, the membranes were washed twice with Tris-buffered saline (150 mM NaCl, 10 mM Tris–HCl, 0.05% v/v Tween-20, pH 7.5) and blocked for 1 h at RT. Carbo-Free Blocking Solution for lectin blot (VectorLabs) and 5% skim milk in Tris-buffered saline containing 0.1% Tween-20 for Western blot. Then, the membranes were treated with Cy5-labeled lectins (2 μg/ml in Carbo-Free Blocking Solution) or antibody diluted overnight at 4 °C in the dark. On the second day, polyvinylidene fluoride membranes continued to incubate with secondary antibodies (it is unnecessary for lectin histochemistry). After washing, exposure scanning can be performed. The following primary antibodies were used: OGT at 1:1000 (Abcam, Cat# ab96718, RRID: AB_10680993), RL-2 at 1:1000 (Abcam, Cat# ab2937, RRID: AB_303264).

### qRT-PCR

RNAiso Plus (Takara, Cat# 9108) was used to extract RNA from 5 to 10 zebrafish fin-regenerating tissue each duplicate. Using Transcriptor First Strand cDNA Synthesis Kit (Roche, Cat# 04897030001), 1 μg of total RNA was synthesized into complementary DNA. SYBR Premix Ex Taq (Takara, Cat# 640022) and a CFX96 Real-Time PCR Detection System (Bio-Rad) were used for the quantitative real-time polymerase chain reactions (qRT-PCR) study. The settings were 95 °C for 10s, 40 cycles at 58 °C, and 72 °C. PCR primers used in this study were shown in Supplemental Table S8.

### Whole Mount *In Situ* Hybridization

PCR was used to clone and ligate 650 bp *ogt* and 703 bp *oga* into the pGM-T vector for culture and transformation. Sequencing established the direction of the connection between the target gene and the vector, enzyme digestion created the linearized plasmid, SP6 RNA (Roche) polymerase created the antisense probe using the linearized plasmid as a template, and digoxin labeled the probe. After 2 to 6 h in 4% paraformaldehyde, fin tissue was gradient dehydrated and rehydrated. Following the addition of Protease K (Merck, 10 μg/ml), the mixture was incubated with continuous shaking for 30 min. Before overnight hybridization with RNA probe at 68 °C, pre-hybridization (Prehyb) solution (Deionized formamide 25 ml, 20 × SCC 12.5 ml, 1 mg/ml yeast RNA 50 mg, 1% Tween-20 0.5 ml, and DEPC water 12.5 ml) was added at 56 °C for 1 h. After recovery, the probe was washed, coated with Roche 1 × blocking solution for 2 h, added antibody solution, and stained overnight at 4 °C. After adding alkaline phosphatase buffer (100 mM Tris–HCl pH 9.5, 100 mM NaCl, 50 mM MgCl2, and 0.2% Tween-20) for prestaining and development buffer for the final time in the dark, the probe's staining time is around 4 h (20).

### Microinjection

Zebrafish were anesthetized under the microscope (Nikon, SMZ800) and pressure microinjection apparatus (Warner, PLI-100A), then respectively injected Alloxan 3 μl (200 mM, Sigma-Aldrich, Cat# A7413), OSMI-1 3 μl (35 mM, Sigma-Aldrich, Cat# SML1621), thiamet-G 3 μl (10 mM, Selleck, Cat# S7213), glucose 3 μl (200 mM, Sigma-Aldrich, Cat# G8270) into the ventral fin and isopycnic dimethyl sulfoxide into the dorsal caudal fin. Put them back into the water to restore vitality (21).

### Purification of O-GlcNAcylated Protein, and MS Analysis

Briefly, 200 μg proteins were precipitated and washed in chloroform/methanol, resuspended in 1% SDS with 20 mM Hepes (pH 7.9), and heated at 90 °C for 10 min until fully dissolved. Protein samples were added to labeling buffer (125 mM NaCl, 50 mM Hepes, 5% NP-40, pH 7.9), MnCl2, 0.5 mM UDP-GalNAz, and Gal-T enzyme (a gift from Prof. Guan Feng, College of Life Sciences, Northwest University, Xi'an, China), incubated overnight at 4 °C, and processed by Click-iT Protein Reaction Buffer Kit (Invitrogen, Cat# C10276). Streptomycin-tagged magnetic beads enriched O-GlcNAcylated proteins, which were digested overnight at 37 °C by trypsin (Promega; Cat# VA9000) at 1:100 (w/w). Using a magnetic frame (Beyotime, Cat# P2151), peptides were isolated and collected by C18 column (Waters Corp, Cat# WAT036905) (22, 23) (Supplemental Fig. S4). The peptide samples were analyzed by LC-MS/MS on an Orbitrap Fusion Lumos Mass Spectrometer (Thermo Fisher Scientific). Samples were separated on a 75 mm × 50 cm nanoViper PepMapTM100 C18 analytical column after being put onto a 75 mm × 2 cm nanoViper PepMapTM100 C18 precolumn. The composition of the mobile phase was 0.1% trifluoroacetic acid (TFA) and 0.1% trifluoroacetic acid/80% acetonitrile. The following settings were made for the gradient profile (220 min): 3 to 7% B for 2 min, 7 to 35% B for 156 min, 35 to 68% B for 40 min, 68 to 99% B for 10 min, and 99% B for 12 min in each case. The mass spectrometry settings were configured as follows: the orbitrap spectra (automatic gain control, 4 × 10^5^) for MS1 had a scan range of 350 to 1, 800 m/z at a resolution of 60 K. For MS2, higher-energy collisional forces broke apart the multiply charged ions in the collision cell. Higher-energy collisional dissociation (collision energy 30%) was used in the collision cell for MS2 to fragment the multiply charged ions, with an isolation window of 1.6 m/z, a maximum injection duration of 30 ms, a resolution of 15 K, and an automatic gain control target of 5 × 10^4^. All Raw files generated by MS were analyzed using proteome search software (Proteome Discoverer 2.3, PD). Zebrafish protein sequence database is downloaded from UniProt database in September 2023 (http://www.uniprot.org). A total of 16,369 peptides were matched and 2865 proteins were identified through searching the UniProt database (September 2023) (Supplemental Table S11-1, MS/MS spectrum database search analysis summary). Fixed modifications were set to carbamidomethyl (C), and variable modifications included oxidation (M), acetyl (N terminus), Met-loss (M), and Met-loss + acetyl (M) (Supplemental Table S11-4, Peptide quantification information). Precursor and fragment mass limits were set at 10 ppm and 0.02 Da, respectively; the filter for protein and peptide identification was 1% protein false discovery rate, and the peptide identification needed at least two peptide-spectrum matches. Proteins in different samples were relatively quantified using the LFQ technique in PD. LFQ was used to calculate protein abundances. The median of all pairwise ratios computed between the three replicates of each peptide abundance was used to determine protein ratios.

### Measurements of Zebrafish and Image Processing

Live photographs of zebrafish fins were captured using a Canon EOS 600D camera, and whole mount *in situ* hybridization (WISH) images were captured using an Olympus SZX10 microscope. The regrowing fin morphology was examined using a dissecting microscope (Olympus). The immunofluorescence pictures were captured using a laser confocal microscope (FV1000, Olympus).

### Quantitative and Statistical Analysis

GraphPad Prism V. 9.0 (https://www.graphpad.com/) conducted statistical analysis. Each experiment was repeated three times. Data were mean ± SD. Student's two-tailed *t* test with *p* < 0.05 was used to compare groups.

## Results

### Alterations in Tissue Glycopatterns during Zebrafish Fin Regeneration

**Fig. 2:**
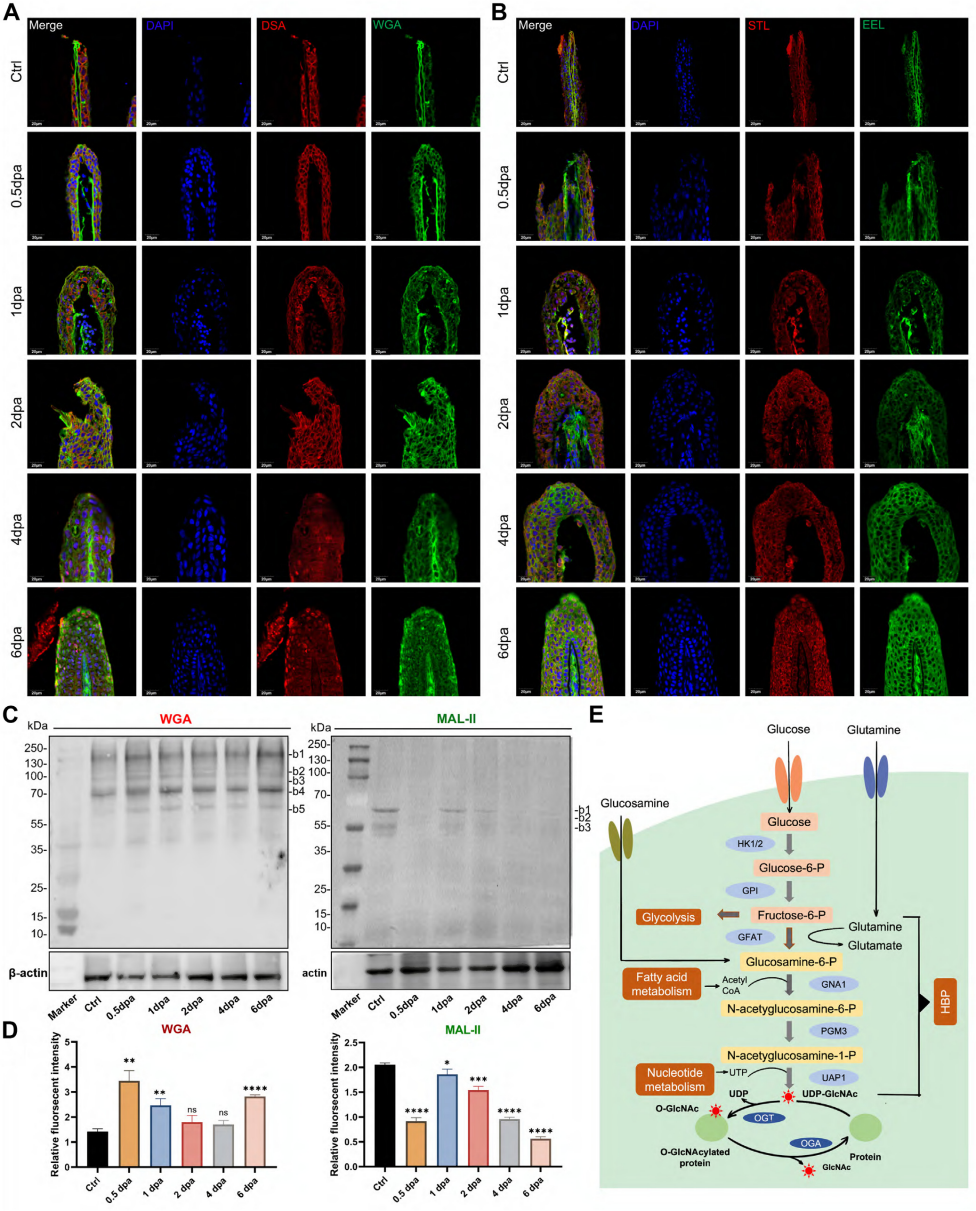
**Validation of the differential expressions of the glycopatterns in zebrafish fin tissues.***A*, triple-labeling of Cy3-DSA and Cy5-WGA-binding glycan increased during the regeneration process (*blue* labeling of nuclei by DAPI; *red* labeling of the perinuclear cytoplasm and the cell membrane by Cy3-DSA; *green* labeling of the perinuclear cytoplasm and the cell membrane by Cy5-WGA) compared with the control. The scale bars represent 20 μm. *B*, triple-labeling of Cy3-STL and Cy5-EEL-binding glycan increased during the regeneration process (*blue* labeling of nuclei by DAPI; *red* labeling of the perinuclear cytoplasm by Cy3-STL; *green* labeling of the intercellular substance by Cy5-EEL) compared with the control. The scale bars represent 20 μm. *C*, the binding pattern of glycoproteins from fin tissues using Cy5-WGA and Cy5-MAL-II blot. The major increased fluorescence intensities WGA glycoproteins located in the b4 with approximately 70 kDa. The major decreased fluorescence intensities MAL-II glycoproteins located in the b1 with approximately 60 kDa. *D*, the fluorescence intensities of the major difference bands were read by ImageJ and showed by relative value in the histogram. Mean ± SEM. Student’s *t* test. ∗*p* < 0.05, ∗∗*p* < 0.01, and ∗∗∗*p* ≤ 0.001, n = 3. *E*, synthesis of O-GlcNAc. DAPI, 4′,6-diamidino-2-phenylindole; O-GlcNAc, N-acetylglucosamine.

There are 37 kinds of lectins arranged on the microarray chip. The microarray was employed to find changes in glycosylation during zebrafish fin regeneration. Figure 1, *A* and *B* illustrated the lectin microarray arrangement and the glycoprotein patterns during zebrafish fin regeneration. The data produced from each regeneration time point were examined using Expander 6.0. The lectins were divided into three main classes using hierarchical cluster analysis. Also, heatmap data clearly showed that lectin signals in the upper group (from DBA to AAL) displayed a rising trend during fin regeneration. In contrast, lectin signals in the bottom group (from BPL to GSL-II) exhibited a decreasing trend (Fig. 1*C*). By quantifying the normalized fluorescence intensities, the distinct patterns between the two clusters were confirmed, and the findings revealed that the majority of the lectin signals were considerably changed in their respective clusters. For instance, throughout the regeneration process, the signals of WGA, STL, DSA, ConA, BS-I, UEA-I, EEL, and PHA-E + L showed a considerable rise (ratio (0.5–6 dpa/Ctrl) > 1.5), whereas the signals of MAL-II, SNA, NPA, SBA, MPL, RCA120, PTL-II, and LTL showed a decrease (ratio (0.5–6 dpa/Ctrl) < 0.67) (Supplemental Table S1). The lectin signals are demonstrably enhanced and lowered in Figure 1, *D* and *E*, respectively. To further validate and assess the expression and distribution of glycosidic residues in zebrafish regeneration fin tissues, fluorescence-based lectin histochemistry was performed with four lectins (DSA, WGA, STL, and EEL) that were randomly selected from lectins that showed significant difference during progression of fin regeneration by lectin microarrays. Each Cy3 and Cy5 labeled-lectin detected the targeted sugar structures present on six sets of tissue sections including Ctrl, 0.5 dpa, 1 dpa, 2 dpa, 4 dpa, and 6 dpa tissues. DSA showed moderate binding to the membrane and the cytoplasm of regeneration tissues; this binding was increasingly intensified in the membranes in one dpa and two dpa tissues. In contrast, wheat germ agglutinin (WGA) and EEL showed strong binding to the intercellular substance and little binding to cytoplasm, this binding of WGA was increased in 0.5 dpa, 1 dpa, and 2 dpa tissues. However, this binding of EEL showed strong fluorescence only in 6 dpa. SJA showed moderate binding to the cytoplasm and the nucleus of regeneration tissues, respectively, and particularly strong binding to the cytoplasm in 2 dpa, 4 dpa, and 6 dpa (Fig. 2, *A* and *B*). These results were basically consistent with the results of lectin microarrays.

To further confirm the results of the lectin microarrays, the lectin blotting analysis was performed with WGA and MAL-II staining, respectively. The results of SDS-PAGE demonstrated that the fin protein bands from zebrafish were similar (Supplemental Fig. S1*A*). The WGA staining showed an increased binding tendency from Ctrl, 0.5 dpa, 1 dpa, 2 dpa, 4 dpa, and 6 dpa subjects according to five apparent bands ranging from 60 to 130 kDa. In contrast, the MAL-II staining showed a decreased binding tendency from Ctrl, 0.5 dpa, 1 dpa, 2 dpa, 4 dpa, and 6 dpa subjects according to three apparent bands ranging from 55 to 60 kDa (Fig. 2, *C* and *D*). These results were basically coincident with the results from the lectin microarrays.

### O-GlcNAcylation Expression Increases During Zebrafish Fin Regeneration

O-GlcNAc synthesis occurs through the hexosamine biosynthesis pathway (HBP), which uses glucose, acetyl-CoA, glutamine, and UTP to produce UDP-GlcNAc (24). Although only about 2 to 3% fructose-6-phosphate enters the HBP, it can reflect the nutritional status of the cell and thus be used for corresponding regulation. O-GlcNAc glycosylation is a dynamic and reversible process controlled by two highly conserved enzymes: OGT, which catalyzes O-GlcNAc glycosylation, and O-GlcNAcase (OGA), which catalyzes deglycosylation (25) (Fig. 2*E*).

**Fig. 3:**
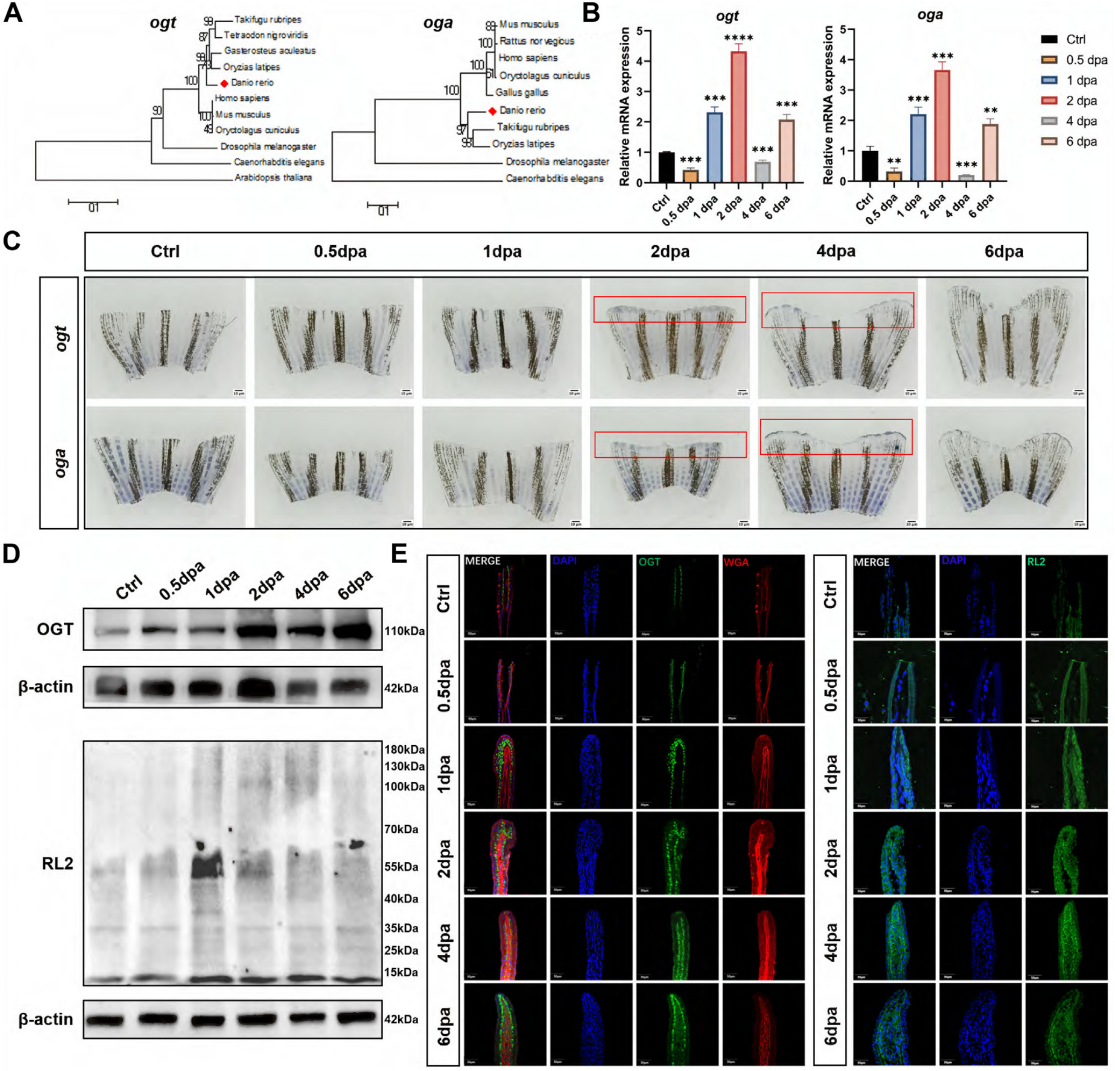
**Expression pattern of OGT and O-GlcNAc during zebrafish fin regeneration.***A*, phylogenetic analysis of *ogt* and *oga*. *B*, relative mRNA expression levels of *ogt* an*d oga* during zebrafish fin regeneration. Mean ± SEM. Student’s *t* test. ∗*p* < 0.05, ∗∗*p* < 0.01, and ∗∗∗*p* ≤ 0.001, n = 3. *C*, spatiotemporal expression pattern of *ogt* and *oga* during zebrafish fin regeneration by using whole-mount *in situ* hybridization. Newly regenerated photoreceptors are indicated with *red* frame. The scale bar represents 10 μm. *D*, protein levels of OGT and O-GlcNAcylation were measured using Western blot. *E*, IF staining for expression of OGT and in control or amputation treatments. *Red dashed boxes* indicate the higher-magnification images in right panels. The scale bar represents 50 μm. OGT, O-GlcNAc transferase; O-GlcNAc, N-acetylglucosamine.

To gain insight into the relationship between O-GlcNAcylation and fin regeneration-associated events in zebrafish, we measured the alteration of O-GlcNAcylation and OGT. Phylogenetic trees of OGT and OGA protein sequences from zebrafish and other animals were constructed by the neighbor-joining algorithm with MEGA 6.06 software (https://www.megasoftware.net/). *Ogt* and *oga* of zebrafish have homology with *Homo sapiens*, *Mus musculus*, and *Oryctolagus cuniculus* to some extent, but there are still some differences due to different species (Fig. 3*A*). qRT-PCR analysis was used to examine the dynamic change in *ogt* and *oga* expression during fin regeneration. In line with the findings of the WISH analysis, the qRT-PCR data showed that the levels of *ogt* and *oga* were elevated after amputation, reached a peak at 2 dpa, and then gradually decreased until regeneration was complete (Fig. 3*B*). Samples were fixed at various regenerating times and submitted to WISH. The blue staining of newly regenerated blastema and regrown fin areas changed from shallow to deep as the regeneration time increased. The 2 dpa and 4 dpa showed the change the most clearly (Fig. 3*C*). Based on our research findings, the transcriptional level of ogt at the wound site exhibits a rapid increase shortly after fin tissue damage. This phenomenon is likely to be the principal driving force behind the elevation of OGT, which subsequently enhances the degree of O-GlcNAc modification.

Western blot analysis and immunohistochemical labeling were used to compare the relative amounts of OGT and O-GlcNAc protein expression in fin tissue to examine the function of O-GlcNAcylation. Figure 3*D* shows that the 2 dpa, 4 dpa, and 6 dpa tissues had considerably greater global levels of OGT than the other groups. Similarly, tissues from the Ctrl group showed significantly lower O-GlcNAc modification abundance than those from the 2 dpa, 4 dpa, and 6 dpa tissues (Figure 3*D*, Supplemental Fig. S1*B*). Also, as shown by the immunofluorescence data displayed in Figure 3*E*, 2 dpa, 4 dpa, and 6 dpa considerably increased the fluorescence intensities of OGT and O-GlcNAcylation compared to the effects on the control group. These findings together revealed that OGT activity and O-GlcNAc alteration were probably connected to the regeneration of the zebrafish fin (Fig. 3*E*).

### O-GlcNAcylation Inhibitor Retards Zebrafish Fin Regeneration

**Fig. 4:**
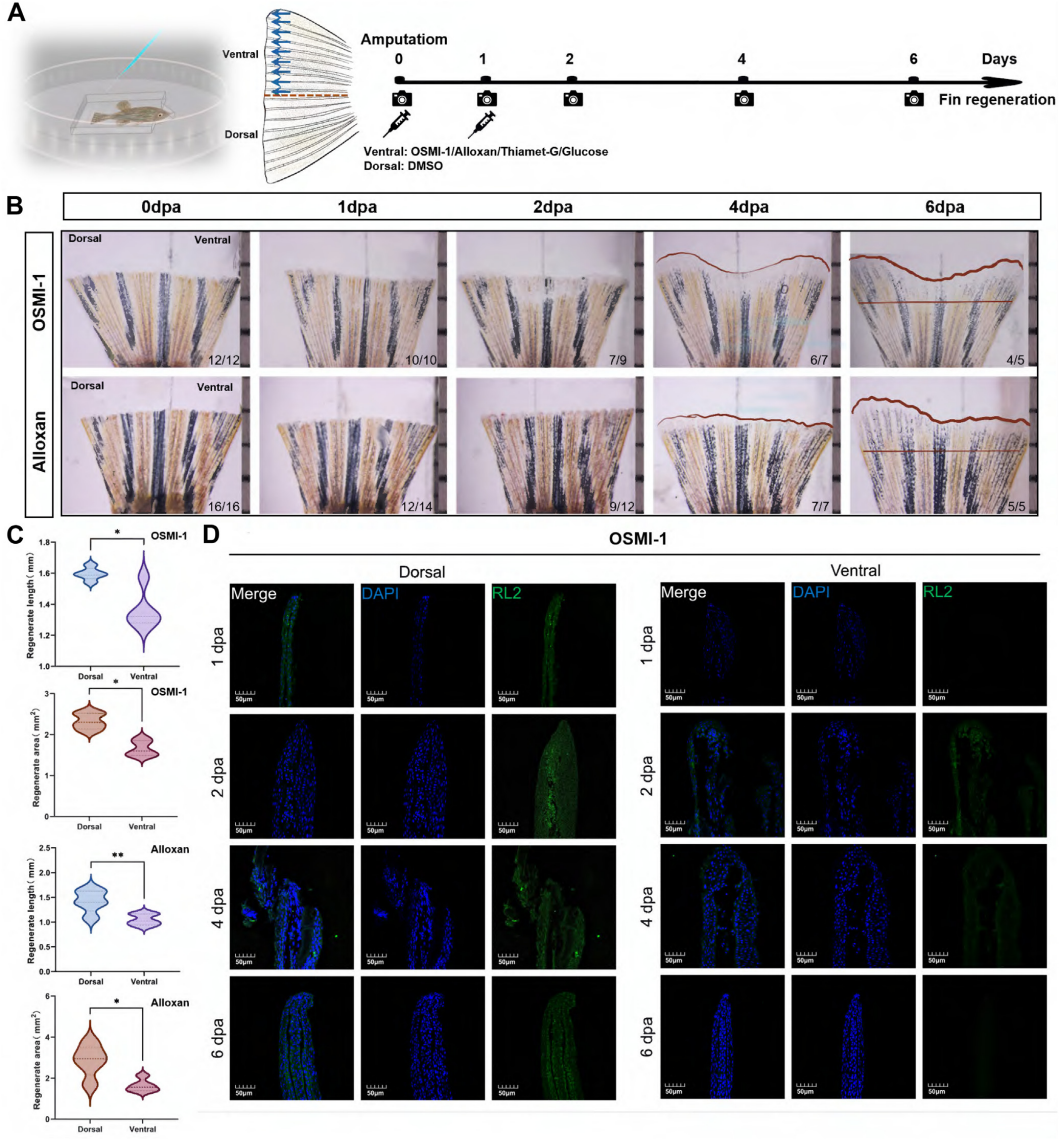
**OSMI-1 and alloxan can suppress zebrafish fin regeneration.***A*, schedule of microinjection. *B*, when OSMI-1 and alloxan was injected into ventral areas of 0 dpa and 1 dpa fin, regeneration was severely suppressed at 4 dpa and 6 dpa, compared with uninjected dorsal fins. In the *bottom right* corner of the picture, the denominator represents the number of fish alive at that point in time, and the numerator represents the number of fish with a regenerative phenotype. *C*, quantification of fin-regenerate lengths and area at 6 dpa. Mean ± SEM. Student’s *t* test. ∗*p* < 0.05, ∗∗*p* < 0.01, and ∗∗∗*p* ≤ 0.001, n = 4 zebrafish in OSMI-1 condition and n = 5 zebrafish in alloxan condition. *D*, RL2 immunofluorescence staining of ventral and dorsal fin when injected with OSMI-1. dpa, days post amputation.

To test whether O-GlcNAcylation is necessary for fin regeneration, we used several strategies to intervene the process. First, we injected the two specific inhibitors of the O-GlcNAcylation (OSMI-1 and alloxan) into the ventral part of the fin stump at 0 dpa (Ctrl) and 1 dpa to explore the functions of O-GlcNAcyaltion in zebrafish fin regeneration (Fig. 4*A*) (26, 27). The immunofluorescence staining assay indicated that OSMI-1 and alloxan could significantly inhibit O-GlcNAc signals compared to the early regeneration stage (1 dpa group), as shown in Figure 4*D*, Supplemental Fig. S2.

The length and area of the injected fin could not regenerate, and depletion of O-GlcNAcylation might even cause the blastema to degenerate (Fig. 4, *B* and *C*). Regeneration of the dorsal fin is normal. These data suggested that OSMI-1 and alloxan mainly exerted inhibitory effects in the middle and later stages of zebrafish fin regeneration.

### Thiamet-G/Glucose Treatment Stimulates Zebrafish Fin Regeneration

**Fig. 5:**
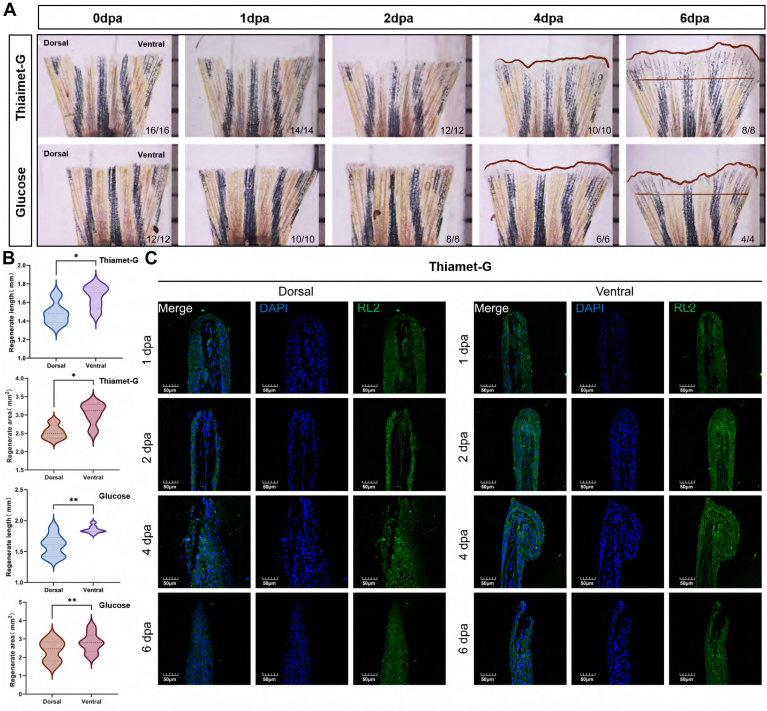
**Glucose and thiamet-G can stimulate zebrafish fin regeneration.***A*, when glucose and thiamet-G was injected into ventral areas of 0 dpa and 1 dpa fin, regeneration was severely stimulated at 4 dpa and 6 dpa, compared with uninjected dorsal fins. *B*, quantification of fin-regenerate lengths and area at 6 dpa. Mean ± SEM. Student’s *t* test. ∗*p* < 0.05, ∗∗*p* < 0.01, and ∗∗∗*p* ≤ 0.001, n = 8 zebrafish in thiamet-G condition and n = 4 zebrafish in glucose condition. *C*, RL2 immunofluorescence staining of ventral and dorsal fin when injected with thiamet-G.

The decreased O-GlcNAcylation level inhibited fin regeneration. Then, we investigated the effect of increasing glycosylation levels on caudal fin regeneration. Thiamet-G as the inhibitor of OGA and glucose as a substrate for HBP could increase O-GlcNAcylation (28, 29). Therefore, they were injected into the ventral fin in this experiment to improve the O-GlcNAcylation level and observe its effect on regeneration. Figure 5*A* shows that thiamet-G and glucose could accelerate ventral fin regeneration. The regenerated length and area of the ventral fin were significantly larger than those of the dorsal fin (Fig. 5*B*). The immunofluorescence staining assay results showed that the expression of O-GlcNAcylation was upregulated in the thiamet-G- and glucose-treated animals at 2 dpa compared with the early regeneration stage (1 dpa group) (Figure 5*C*, Supplemental Fig. S3). The result indicates that thiamet-G and glucose treatment might stimulate fin regeneration via upregulating the O-GlcNAcylation.

### Identification of O-GlcNAcylation Glycoproteins During Zebrafish Fin Regeneration, GO Classification of the Identified O-GlcNAcylation Glycoproteins

**Fig. 6:**
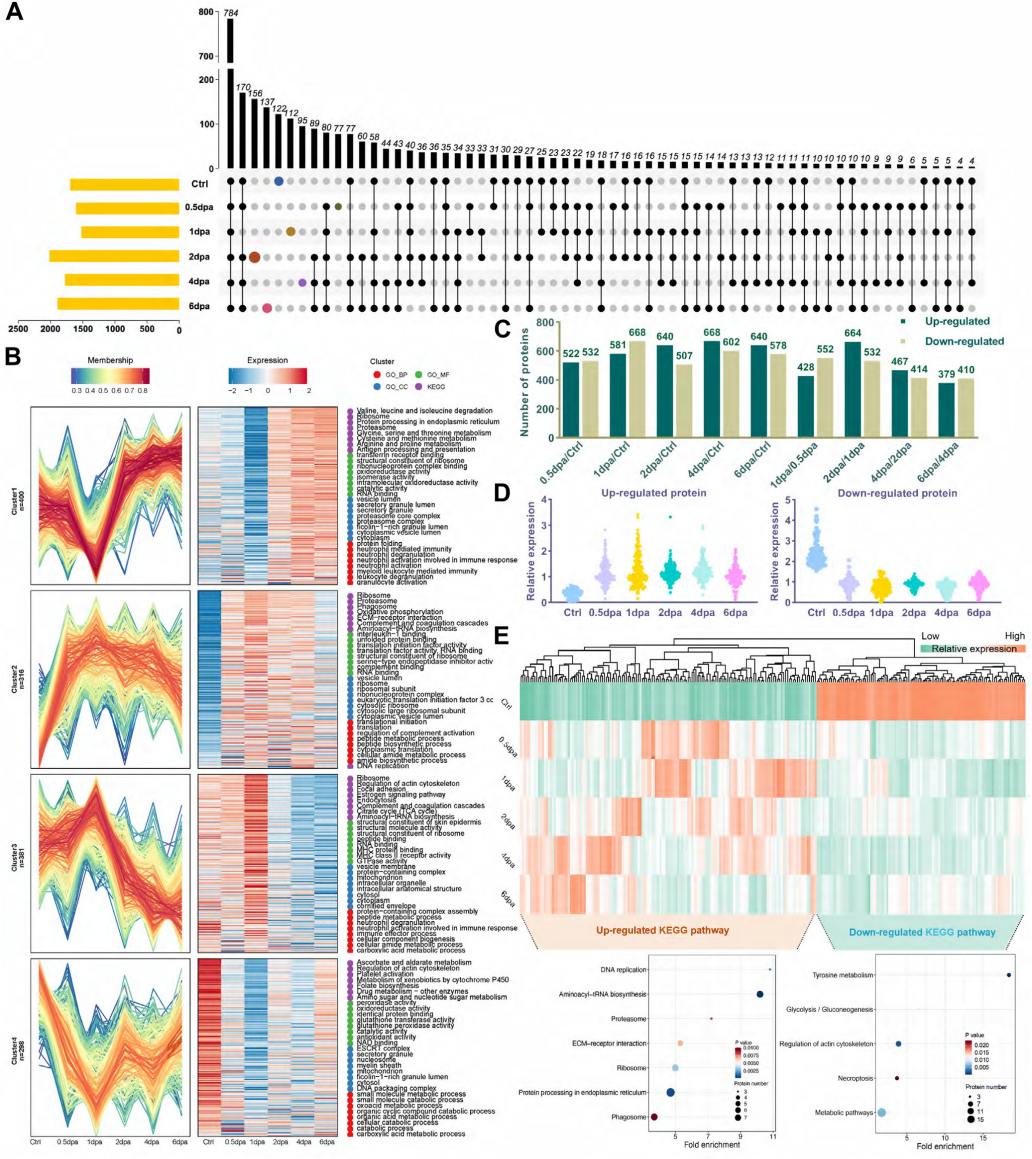
**LC-MS/MS identification and GO annotations of the isolated O-GlcNAcylation glycoproteins during zebrafish fin regeneration.***A*, the Venn diagrams present the numbers of O-GlcNAcylation glycoproteins identified by LC-MS/MS. *B*, total O-GlcNAcylation glycoproteins identified during zebrafish fin regeneration were analyzed for functional enrichment according to four grouping classifications: cellular component, biological process, molecular function, and KEGG. *C*, comparison of O-GlcNAc glycoprotein involved in 0 dpa, 0.5 dpa, 1 dpa, 2 dpa, 4 dpa, and 6 dpa. *D*, expression pattern of upregulated and downregulated proteins. *E*, heatmap, KEGG pathways of upregulated and downregulated O-GlcNAc modification proteins in regeneration fin. LC-MS/MS, liquid chromatography-tandem mass spectrometry; dpa, days post amputation; KEGG, Kyoto Encyclopedia of Genes and Genomes; O-GlcNAc, N-acetylglucosamine.

In order to provide a preliminary insight into the role of O-GlcNAcylation glycoproteins in zebrafish during fin regeneration, the O-GlcNAcylation glycoproteins were isolated from fin tissue protein using a chemical enzyme label and digested by trypsin, then identified by LC-MS/MS (Supplemental Fig. S4*A*). When enriching O-GlcNAc proteins, protein samples without the addition of Gal-T1 were taken as the negative control group. The click chemical reaction was carried out simultaneously with the experimental group. Horseradish peroxidase-labeled streptavidin was used as the primary antibody, and Western blot was employed to detect the specificity of this method (Supplemental Fig. S4*B*). A total of 1595, 1525, 1445, 1892, 1678, and 1778 O-GlcNAcylation proteins were identified from Ctrl, 0.5 dpa, 1 dpa, 2 dpa, 4 dpa, and 6 dpa, respectively. Furthermore, the glycoproteins were exclusively identified in the regenerating animals at Ctrl, 0.5 dpa, 1 dpa, 2 dpa, 4 dpa, and 6 dpa were listed in Supporting Information Supplemental Tables S2–S7 (Fig. 6*A*).

To investigate the major biological functions of zebrafish fin regeneration O-GlcNAcylation glycoproteins, FunRich (http://funrich.org/download) was applied to analyze the total unique proteins for functional enrichment according to three grouping classifications: cellular components, biological processes, and molecular functions (Supplemental Fig. S5). Through Gene Ontology (GO) enrichment analysis, we found that some differences in biological processes involved in O-GlcNAcyaltion glycoproteins were related to zebrafish fin regeneration. Therefore, the biological processes that might be related to regeneration were compared, as shown in Supplemental Table S9.

We used the Mfuzz method to perform cluster analysis on the protein expression of these continuous samples. The proteins were divided into four clusters, obtaining four different protein expression trends among these six groups of samples. To further understand the biological processes in which the proteins in each cluster participate, we performed GO functional and Kyoto Encyclopedia of Genes and Genomes (KEGG) pathway enrichment analyses on the proteins in each cluster, respectively. The hierarchical clustering analysis of these differentially expressed proteins showed that there were significant differences in the biological processes and signal pathways involved in the proteins of each cluster (Fig. 6*B*). We used PD to perform quantitative analysis of the complete peptide segments in the six protein samples and screened out the glycoproteins corresponding to the differentially expressed complete peptide segments. Among them, there were 552 upregulated and 532 downregulated glycoproteins in the 12 hpa, 581 upregulated and 668 downregulated glycoproteins in the 1 dpa, 640 upregulated and 507 downregulated glycoproteins in the 2 dpa, 668 upregulated and 602 downregulated glycoproteins in the 4 dpa, and 640 upregulated and 578 downregulated glycoproteins in the 6 dpa (Fig. 6*C*). We then screened out proteins that are co-high and co-low expressed during the regeneration phase. The expression trend of these proteins was analyzed by cluster analysis (Fig. 6, *D* and *E*). Both upregulated proteins are involved in the extracellular matrix (ECM)-receptor interaction pathway, and so on. Moreover, both downregulated proteins are involved in the metabolic pathway, and so on (Fig. 6*E*, Supplemental Table S10).

### KEGG Pathway Analysis and Functional Protein Association Networks

The separated O-GlcNAc binding proteins were then further examined for their involvement in a variety of biological processes, such as energy metabolism, glycolysis and gluconeogenesis, signal pathway, and tumor growth, using the KEGG network analysis tool.

**Fig. 7:**
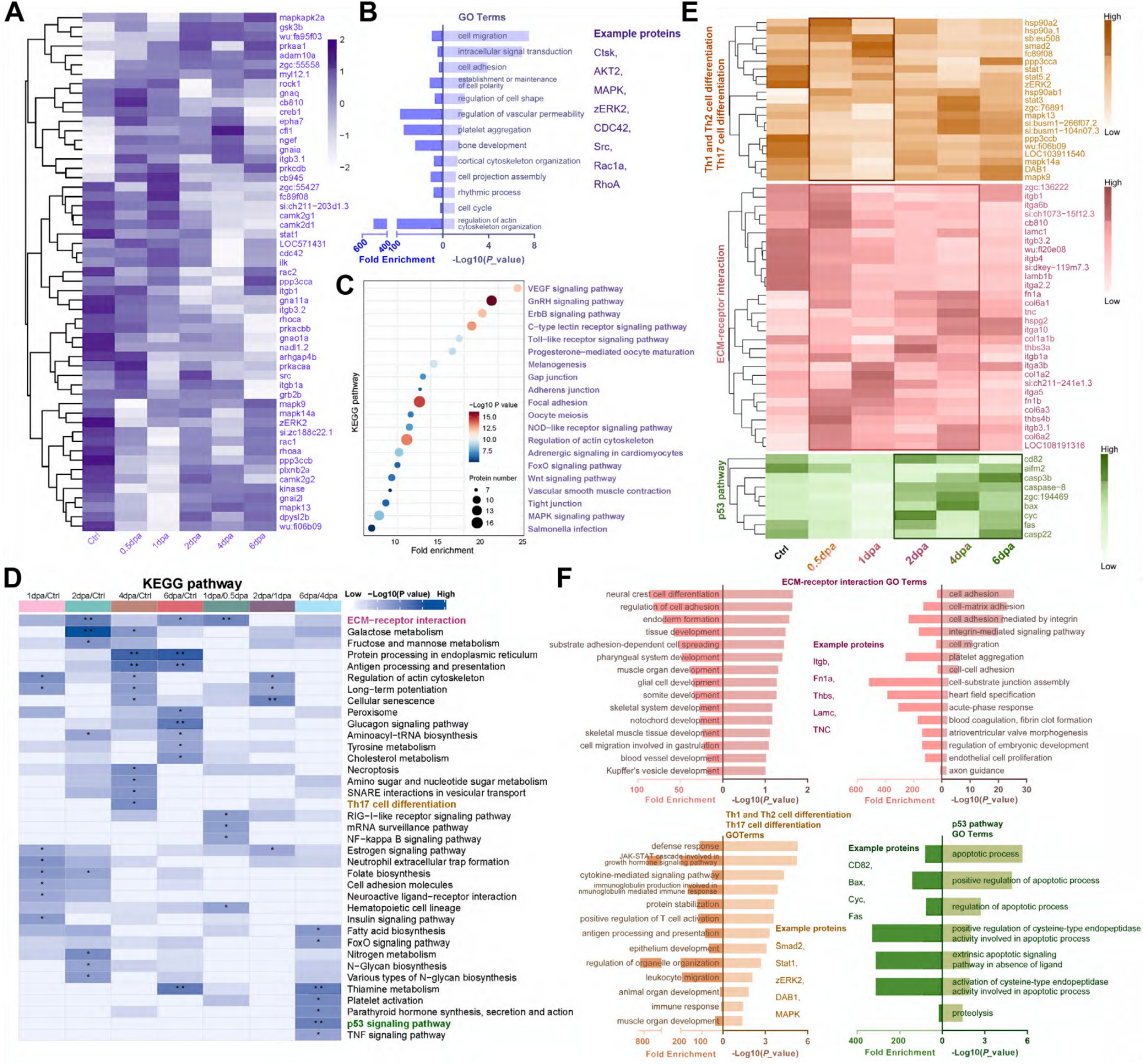
**Clusters of Orthologous Groups (COG) and Eukaryotic Orthologous Groups (KOG), GO, KEGG analysis in O-GlcNAcylation protein of fin regeneration.***A*, heatmap of development and regeneration related proteins expressed in regeneration fin. *B*, enriched GO terms and examples of development and regeneration related proteins. *C*, KEGG enrichment in O-GlcNAcylation protein of fin regeneration. *D*, KEGG pathway analysis of fin regeneration. *E*, heatmap of O-GlcNAcylation proteins enriched in ECM-receptor interaction, Th1 and Th2 cell differentiation, Th17 cell differentiation, p53 pathway. *F*, enriched GO terms and examples of O-GlcNAcylation proteins enriched in ECM-receptor interaction, Th1 and Th2 cell differentiation, Th17 cell differentiation, p53 pathway. ECM, extracellular matrix; GO, gene ontology; KEGG, Kyoto Encyclopedia of Genes and Genomes; O-GlcNAc, N-acetylglucosamine.

The results showed several pathways most closely related to the differentially expressed glycoproteins. KEGG pathway enrichment analysis of O-GlcNAc glycoprotein during zebrafish fin regeneration was carried out using DAVID online software (https://www.baidu.com/link?url=YZN-z9hwTILc13rbL9HiM_mkls-GfBmFZL4uz8sHUXevkozn36lv_UEiQGgLXv6g&wd=&eqid=ee3a25d4000014ec0000000267dbccc3). We performed a comparative analysis of mass spectrum data to cluster the differentially expressed proteins associated with developmental and regenerative pathways (Fig. 7*A*, Supplemental Figs. S6–S9, Supplemental Table S11). Briefly, proteins were assigned to GO terms that included bone development, regulation of cell shape, and cell cycle, which are strongly associated with fin regeneration. Proteins involved in the pathway such as MAPK signaling, VEGF signaling, FoxO signaling, and so on. Those have been shown to be involved in regenerative regulation (Fig. 7, *B* and *C*).

After a unified comparison of KEGG pathway with differentially expressed proteins in each group, we found most of the O-GlcNAc glycoproteins involved in pathways were similar, with high levels of enrichment in several pathways: Th1 and Th2 cell differentiation, Th17 cell differentiation, ECM-receptor interaction, N-glycan biosynthesis, galactose metabolism, p53 pathway, and so on. However, compared with the ctrl group, several pathways were highly enriched and only appeared in the regeneration stage, presumably related to fin regeneration. (Fig. 7*D*). In the 2 dpa sample, the differentially expressed proteins involved in ECM-receptor interaction pathway included Lamc, Lamb, Thbs, Itga, Itgb, Tnc, Fn1a, Fn1b, and Hspg, and so on (Supplemental Fig. S11). The differential expressed proteins in the ECM-receptor interaction pathway were aligned with the following GO terms: notochord development, skeletal system development, tissue development, and so on. In the 0.5 dpa sample, the differentially expressed proteins involved in Th1 and Th2 cell differentiation and Th17 cell differentiation pathway included STAT, MAPK, Smad2, zERK2, and so on. The differential expressed proteins in the pathway were aligned with the following GO terms: muscle organ development, immune response, regulation of organelle organization, and so on. In the 6 dpa sample, the differentially expressed proteins involved in p53 signaling pathway included CD82, Bax, Cyc, and so on. The proteins were related to the GO terms regulation of apoptotic process (Fig. 7, *E* and *F*). These results suggest that the related differentially expressed proteins may provide valuable information for the molecular mechanisms of different regeneration stages in zebrafish caudal fin, indirectly providing possibilities for tissue repair and limb regeneration. In summary, we have systematically elucidated the biological functions of O-GlcNAc proteins in zebrafish fin regeneration at five different regeneration nodes from the perspective of proteomics and provided a series of potential molecular candidates involved in regeneration regulation.

## Discussion

Protein glycosylation and alterations in these modifications have significant impacts on certain biological processes, such as cell proliferation, recognition, and adhesion, during the regeneration process. Various signaling pathways are involved in regulating different regeneration stages in zebrafish organs, such as the caudal fin, heart, spinal cord, and retina, and most of the signaling molecules involved in this regulation are glycoproteins. Current research indicates that the orderly processes of cell dedifferentiation, proliferation, and redifferentiation during regeneration are regulated by the glycosylation of signaling molecules. Adult mammals have nearly lost all tissue regeneration ability. Amputated limbs, damaged cardiac muscle, brain tissues, and injured spinal cord tissue in adult mammals are unable to self-repair. Therefore, conducting in-depth investigations into the mechanisms governing tissue and organ regeneration in model organisms like zebrafish is of great significance. Such studies can enhance our understanding of similar processes in humans and open up new avenues for the development of regenerative medicine.

In our experiment, GlcNAc glycans emerged as the dominant species among the upregulated glycan structures recognized by lectins. While WGA, STL, ConA, and PHA-E + L are all lectins that similarly recognize GlcNAc, these have different affinities for GlcNAc. As a consequence, when compared to the ctrl (control) group, these lectins generate varying signal intensities and display differences at different time points. The expression of glycans detected by WGA demonstrated a consistent upregulation throughout the fin regeneration process. In contrast, the expression patterns recognized by the other lectins were predominantly upregulated at 1 and 2 dpa. Among the downregulated glycan chains recognized by lectins, sialic acid, galactose, fucose, and high-mannose glycans were predominant. These results indicate that the changes in the glycosylation of fin proteins indeed occur during fin regeneration and follow a certain pattern.

Lectin array analysis revealed a significant increase in O-GlcNAc recognized by WGA, STL, and ConA. Elevated levels of O-GlcNAc glycosylation during fin regeneration are presumed to be induced by elevated levels of *ogt* transcription. Moreover, qRT-PCR and *in situ* hybridization experiments have shown that the transcription of *oga* is elevated leading to enhanced hydrolysis of O-GlcNAc. Intriguingly, this phenomenon does not conflict with the overall elevation of O-GlcNAc glycosylation levels. Under normal conditions, glycosyltransferases and glycosidases are in dynamic equilibrium to maintain the glycosylation levels within a normal range. When the OGT expression is elevated during caudal fin regeneration, leading to elevated levels of O-GlcNAc glycosylation, the organism responds by producing more OGA to hydrolyze O-GlcNAc and restore the O-GlcNAc glycosylation levels back to their normal baseline.

O-GlcNAcylation modification represents a distinctive form of posttranslational modification. The glycosylation modification of O-GlcNAc is highly dynamic and cyclic. Using UDP-GlcNAc as the donor, it is catalyzed by OGT and forms an O-glucoside bond with the serine/threonine residues of proteins. Conversely, O-GlcNAc is hydrolyzed from serine/threonine residues under the action of OGA (30). Changes in external glucose levels can affect UDP-GlcNAc donors through the hexosamine pathway, subsequently, affecting the O-GlcNAc glycosylation modification of intracellular proteins. O-GlcNAcylation plays a regulatory role in signal transmission, transcription, and proteasomal degradation, which are among hundreds of protein posttranslational alterations. It significantly impacts protein function and has been linked to various diseases (25). Moreover, O-GlcNAcylation is also important in embryonic development. Webster *et al.* found that OGT knockdown or overexpression in zebrafish embryos delayed epiboly, decreased brain growth, and increased cell death (10). In zebrafish mutants, indirect enzyme alterations disrupt glycosylation and cause significant actin and tubulin disintegration in the extraembryonic yolk syncytial layer (31). The Pou5f1/Oct4 transcription factor, which is altered by O-GlcNAc in human embryonic stem cells, loses its function in growing embryos, causing cytoskeletal defects (10). O-GlcNAc modification alters Pou5f1 activity in embryonic stem cells and throughout the embryo, which might be essential for morphogenesis and cell survival.

We enriched the proteins modified by O-GlcNAcylation during zebrafish fin regeneration and identified them by mass spectrometry. In regenerating protein samples at different time points, various signaling pathways are enriched, which may be related to the regeneration process. The prediction of glycosylation sites is shown in Supplemental Fig. S10 (https://services.healthtech.dtu.dk/services/YinOYang-1.2/). We hypothesized that these signaling pathways were regulated by the glycosylation of these proteins, which influenced fin regeneration. We enriched the Th1 and Th2, and Th17 cell differentiation-related pathways in 0.5 dpa. We speculate that it might be possible in the early stage of regeneration, the proinflammatory state dominates, while the antiinflammatory state in the later stages plays a key role in promoting tissue repair and regeneration. The O-GlcNAcylaton proteins are involved in these pathways that promote wound healing. This speculation remains to be further validated. Studies have shown that O-GlcNAcylation is involved in the regulation of inflammatory responses and plays an important role. When the body is subjected to inflammatory stimulation, OGT is capable of facilitating the O-GlcNAc modification of NF-κB subunits. This, in turn, effectively drives the translocation of NF-κB into the nucleus and markedly enhances its transcriptional activity (32). This sequential process leads to the significant upregulation of the expression of a series of inflammation—related genes, including tumor necrosis factor-α, interleukin-1β, and chemokines, ultimately culminating in the triggering of an inflammatory response (33). Moreover, O-GlcNAc modification can also exert an impact on the signal transduction within macrophages, inducing macrophages to polarize into the M1 type. Consequently, this polarization strengthens their phagocytic function and augments the release of proinflammatory cytokines (34). Simultaneously, O-GlcNAc modification is intimately intertwined with the survival and apoptosis of inflammatory cells. Both excessively elevated and diminished levels of O-GlcNAc modification are highly likely to disrupt the intracellular balance, giving rise to abnormal apoptosis or excessive proliferation of inflammatory cells. As a result, this affects the progression of inflammation. In numerous chronic inflammatory diseases, such as diabetic complications, cardiovascular diseases, and neuroinflammation, the level of O-GlcNAc modification exhibits substantial and significant changes (35, 36, 37). From these research results, we can speculate that O-GlcNAcylation may also be involved in the regulation of the inflammatory response during fin regeneration. In addition, ECM-receptor interaction signaling pathway is significantly enriched during fin regeneration. This pathway plays a crucial role in influencing cell proliferation and migration, and the genes involved in this pathway may participate in the regulation of body regeneration and development. Cells communicate with each other via ECM receptors, transmitting multiple signals to regulate critical cellular developmental processes such as motility, differentiation, proliferation, survival, and modulating the activity of cytokines and growth factors, as well as directly or indirectly activating intracellular signals (38). Hyaluronic acid, proteoglycans, collagen, glycosaminoglycans, elastin, SPARC, tenascin, osteopontin, and thrombospondin (THBS) are found in the extracellular microenvironment. These proteins were identified from our data and are closely associated with O-GlcNAc modification. Thrombospondin belongs to a family of glycoproteins. In the tumor microenvironment, it serves as a crucial mediator, facilitating interactions between cells and potential matrix components. It significantly impacts angiogenesis, synaptic formation, and the construction of connective tissue. Specifically, THBS4 participates in biological processes such as cell proliferation, adhesion, pain signal transduction, and tumor progression, as noted in reference (39). Heparan sulfate proteoglycans (HSPG) represents a pivotal constituent of the ECM. It exhibits the capacity to bind to a diverse array of ECM molecules, and more importantly, it can efficiently bind to various growth factors. Therefore, if there is a lack of HSPG in the microenvironment after myocardial injury, the efficiency of endogenous or exogenous growth factor action will be reduced, which is unfavorable for tissue injury repair (40). Early research confirmed that HSPG expression is prevalent in regenerating zebrafish fins (41). Our data suggest that O-GlcNAc modification of ECM-receptor interaction signaling pathway plays an essential role in zebrafish fin regeneration.

Our study does have certain limitations. Firstly, the regulation of glycosylation on cell activities is complex and diverse. In our research, changes in various glycans were detected during fin regeneration, and in the later stage, we focused on the mechanism of O-GlcNAcylation. Notably, the sialic acid recognized by MAL-II and SNA showed a significant decline during regeneration. Sialic acid is a negatively charged structure on the cell surface and is the primary reason for the negative charge of the cell surface; thus, it is frequently explored in the context of cell adhesion (42, 43). The level of sialic acid expression is associated with tumor cell adhesion, migration, invasion, and metastasis, and many cancer cells have elevated sialic acid levels on their surface and exhibit decreased cell adhesion, accompanied by increased tumor metastasis. The impact of decreased sialic acid modifications on fin regeneration and the underlying regulatory mechanisms warrant further in-depth exploration. Secondly, the level of glycosylation was changed through *in vivo* experimental injection of inhibitors to observe its effects on fin regeneration. In future, investigations into the impact of O-GlcNAcylation regulation on the fin regeneration mechanism, appropriate interference techniques can be selected for further verification, depending on requirements. Moreover, constructing corresponding mutant or transgenic zebrafish strains is also essential for subsequent research. Thirdly, we discovered that the Th1 and Th2 cell differentiation, Th17 cell differentiation, ECM-receptor interaction, and p53 pathways exhibited significant differences at different regeneration stages through the analysis of O-GlcNAc modified proteomics. This indicates that they are regulated by the O-GlcNAc glycosylation network. However, additional molecular mechanisms need to be validated through relevant molecular biology experiments and *in vivo* studies.

## Conclusion

In this article, we pioneered the identification of glycans with differential expression during zebrafish fin regeneration. The results indicated that the glycosylation modification was changed during fin regeneration. The expression of O-GlcNAcylation was significantly upregulated in the fin regeneration. We conclude that the precise localization and alteration of O-GlcNAcylation associated with pathological changes in fin regeneration may offer crucial information to help understand the biological functions of glycans and how to exert them through their recognition by a wide variety of the O-GlcNAcylation proteins, which could lead to the development of new limb regeneration and repair strategies. During zebrafish fin regeneration, OGT was found to promote the process by upregulating the O-GlcNAcylation level, while OGA inhibited fin regeneration by downregulating this level. Additionally, O-GlcNAc glycoproteins were revealed to play a pivotal role in regulating the regeneration process. This study provides a novel dimension to the exploration of regeneration mechanism. Nevertheless, the full spectrum of functions and effects of the altered O-GlcNAcylation during fin regeneration remains to be comprehensively investigated.

## Data Availability

The mass spectrometry proteomics data have been uploaded to iProX (44, 45) with the dataset identifier IPX0010629000. URL link (https://www.iprox.cn/page/PSV023.html;?url=1740489607103p0eG) and password (R4uv).

## Supplemental data

This article contains supplemental data. Supplemental Data, including Supplementary figure and text, Supplementary Tables S1–S11.

## Conflict of interest

The authors declare no competing interests.
