# Supplementary material for: Upregulation of Protein O-GlcNAcylation Levels Promotes Zebrafish Fin Regeneration
Source: Mol Cell Proteomics. 2025 Mar 4;24(4):100936. doi: 10.1016/j.mcpro.2025.100936 (PMC12002929; doi:10.1016/j.mcpro.2025.100936)
Supplement: Supplementary Figure [file mmc1.docx]

**Supplementary materials**


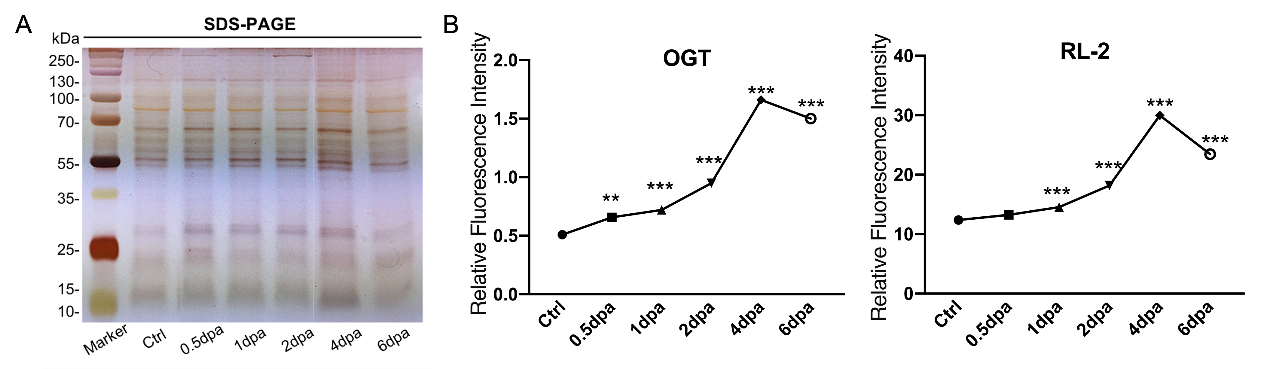


Supplementary Figure 1.

(A) Binding pattern/profiles of proteins from six groups of fin tissue samples using SDS-PAGE silver stain. (B) The fluorescence intensities of the major difference bands were read by ImageJ and showed by relative value of OGT and RL2 in the histogram.


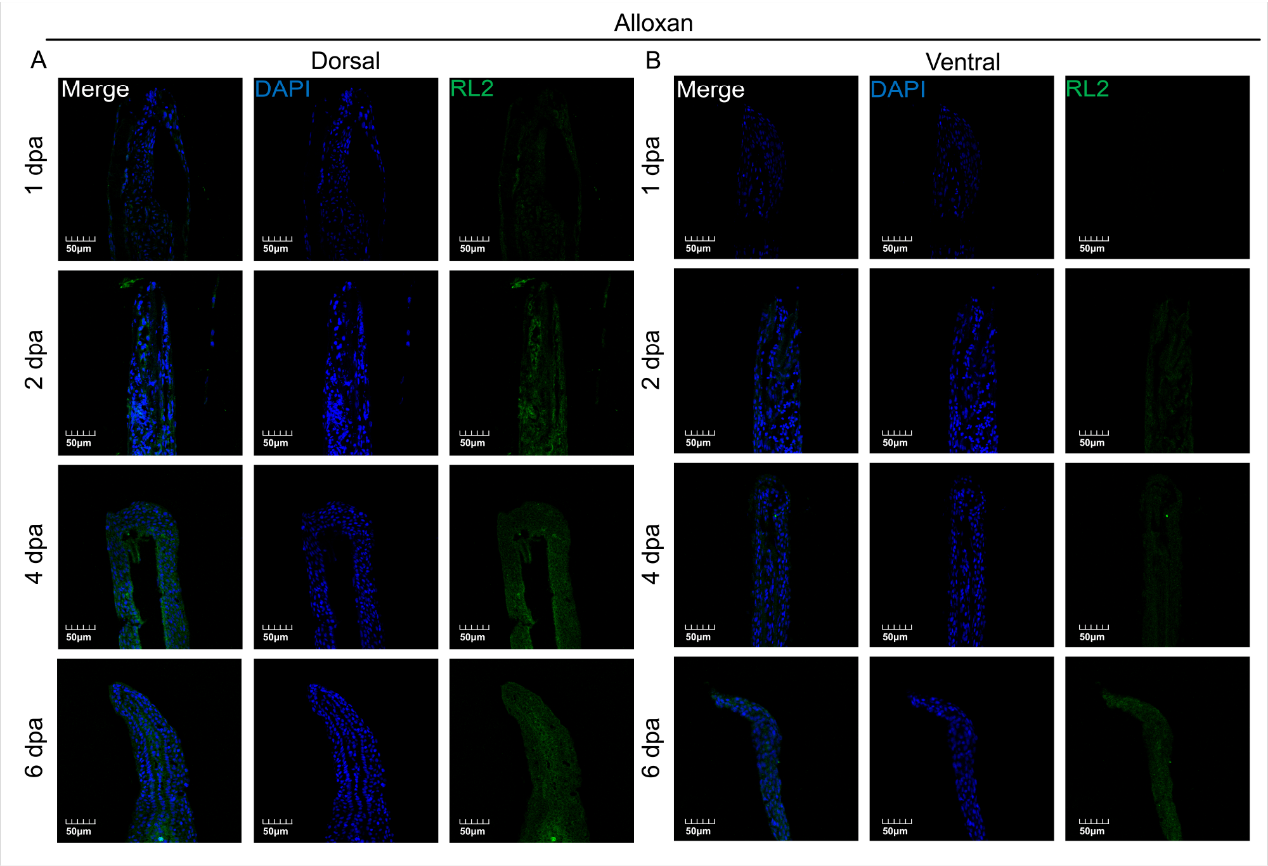


Supplementary Figure 2. RL2 immunofluorescence staining of ventral and dorsal fin when injected with Alloxan.


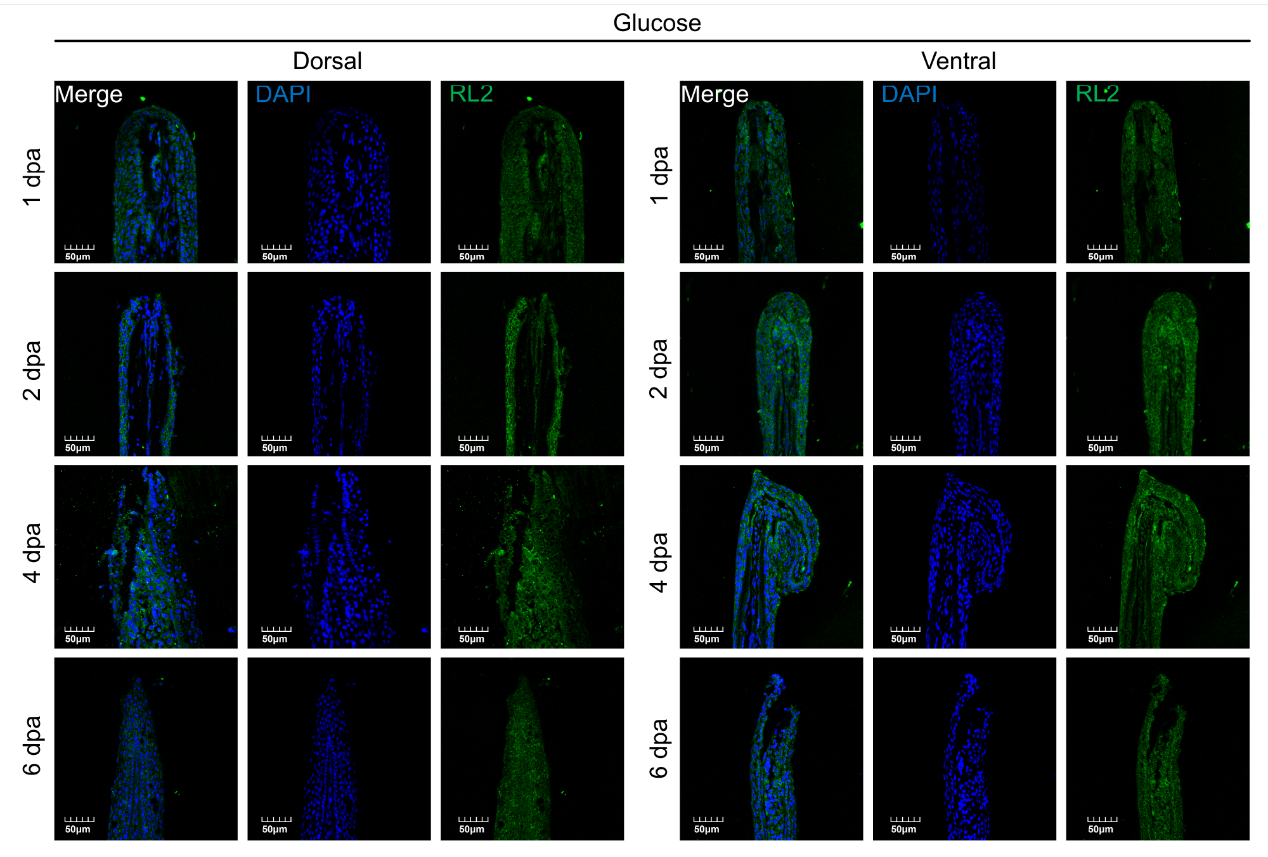


Supplementary Figure 3. RL2 immunofluorescence staining of ventral and dorsal fin when injected with Glucose.


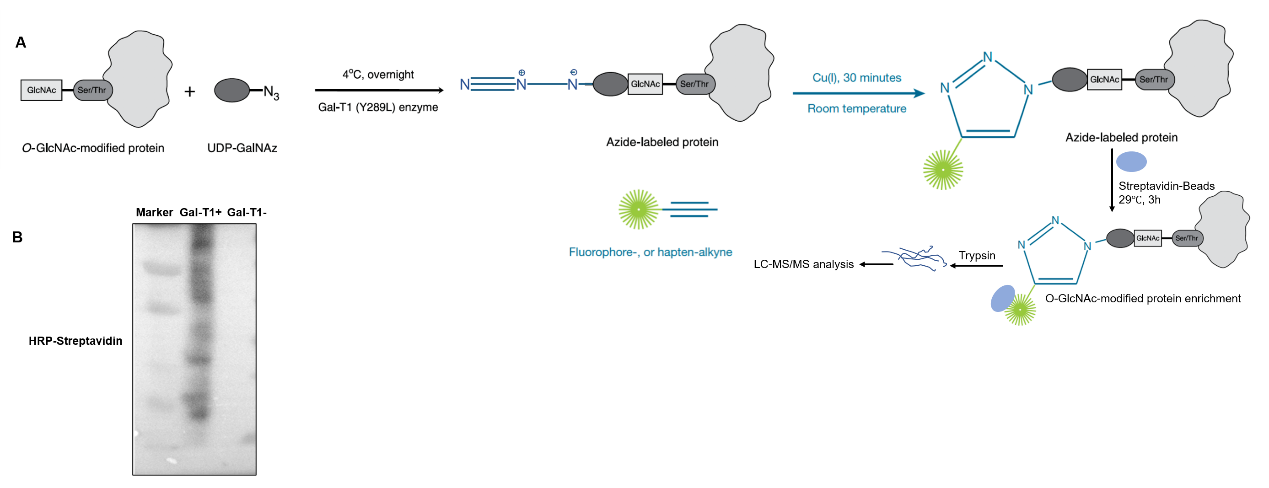
 Supplementary Figure 4. (A) Schematic diagram of O-GlcNAc protein enrichment. (B) Specificity of chemical enzyme labeling and efficiency of protein enrichment.


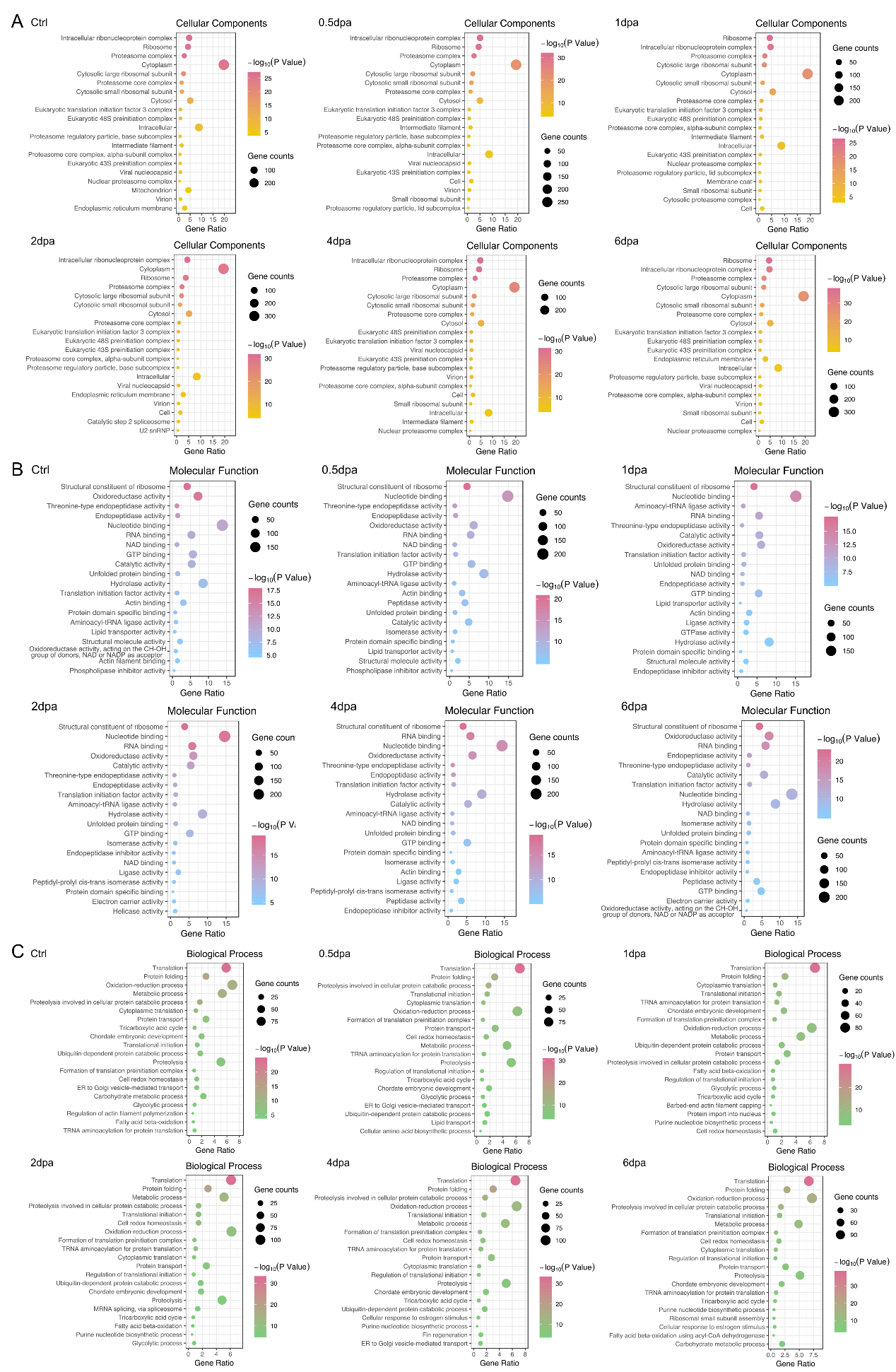


Sullementary Figure 5. Protein BP, CC, MF enrichment analysis bubble map of six groups of fin tissue samples.


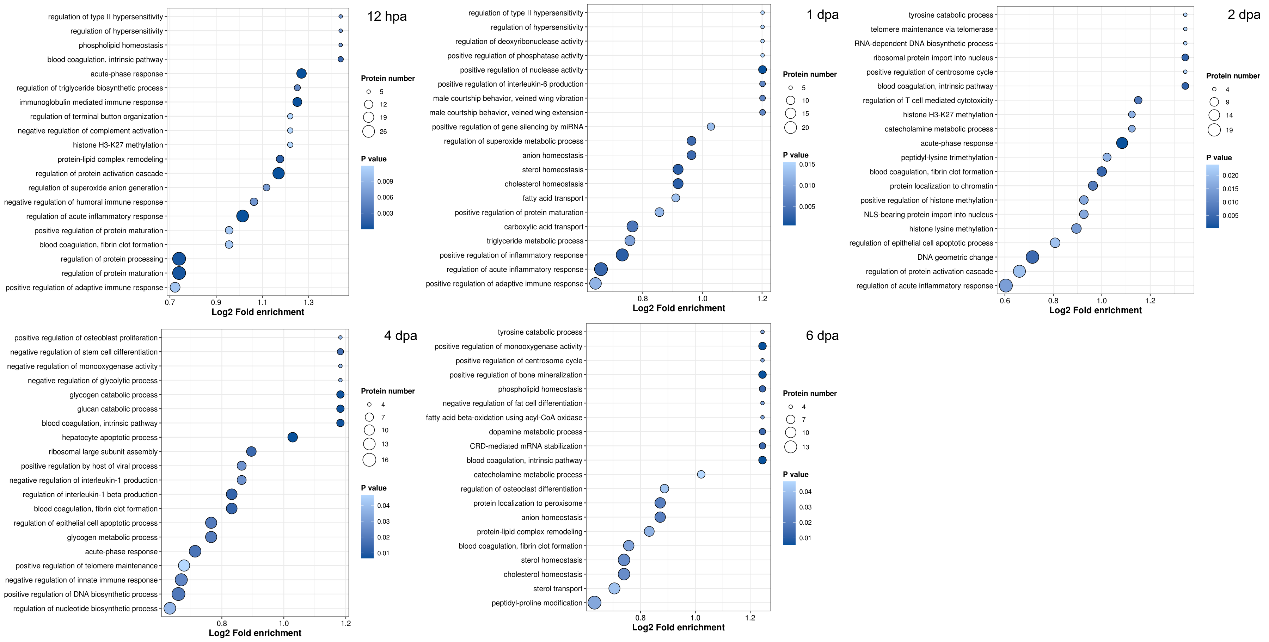


Supplementary Figure 6. Bubble map of BP enrichment analysis of differentially expressed proteins.


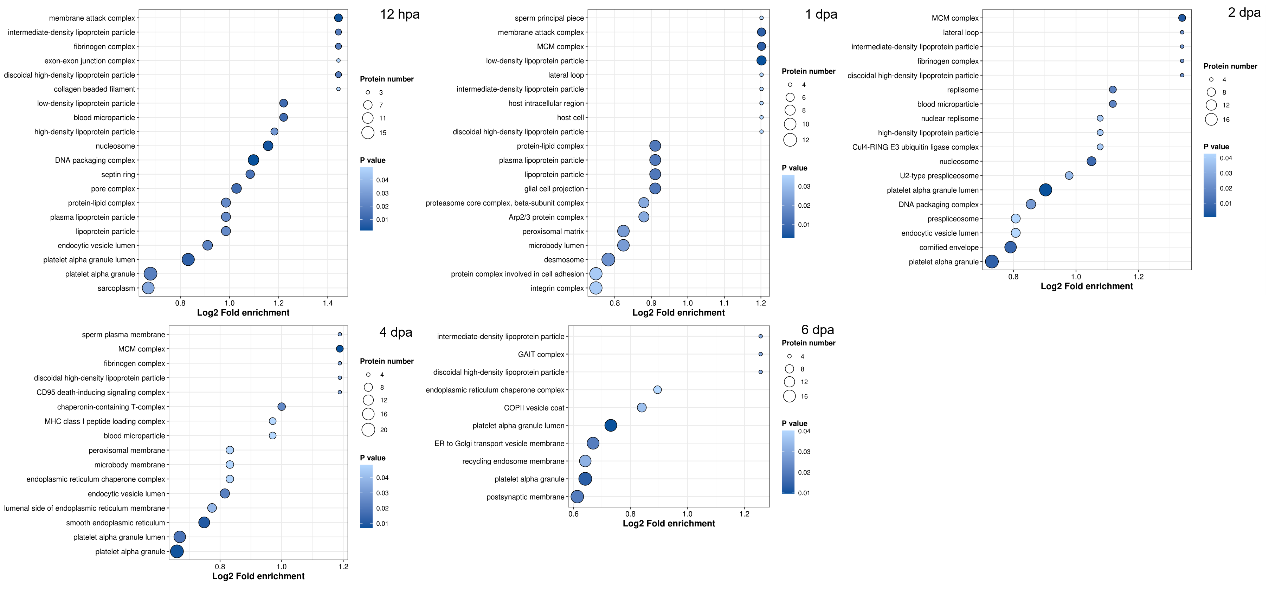


Supplementary Figure 7. Bubble map of CC enrichment analysis of differentially expressed proteins.


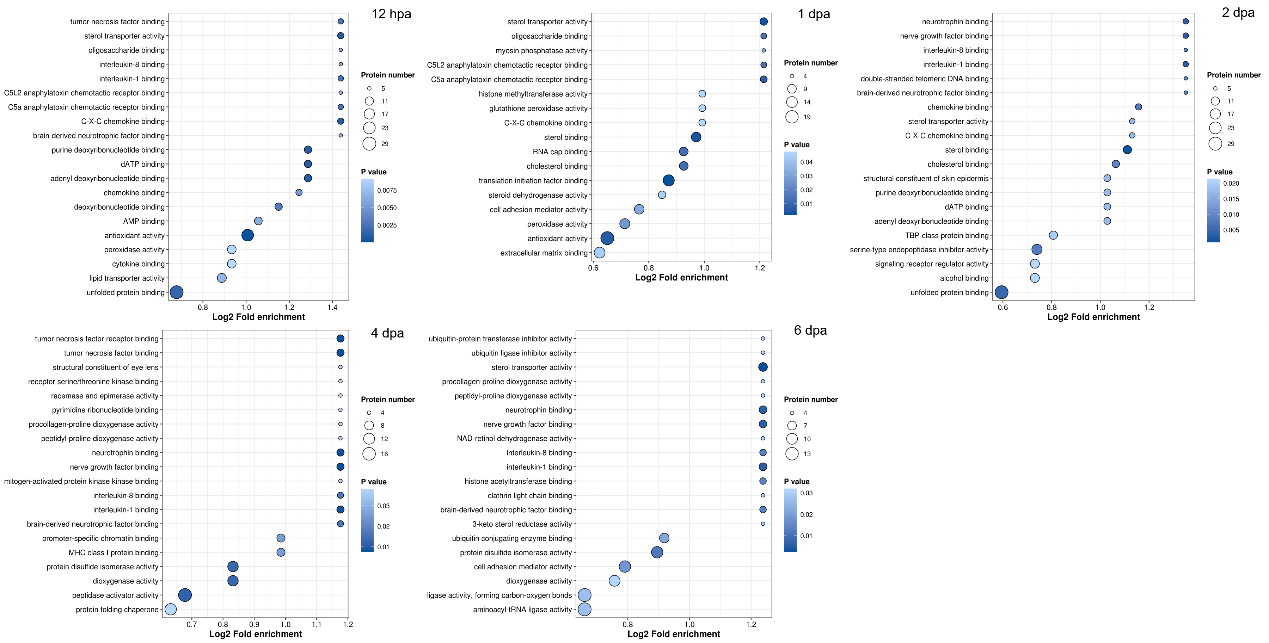


Sullementary Figure 8. Bubble map of MF enrichment analysis of differentially expressed proteins.


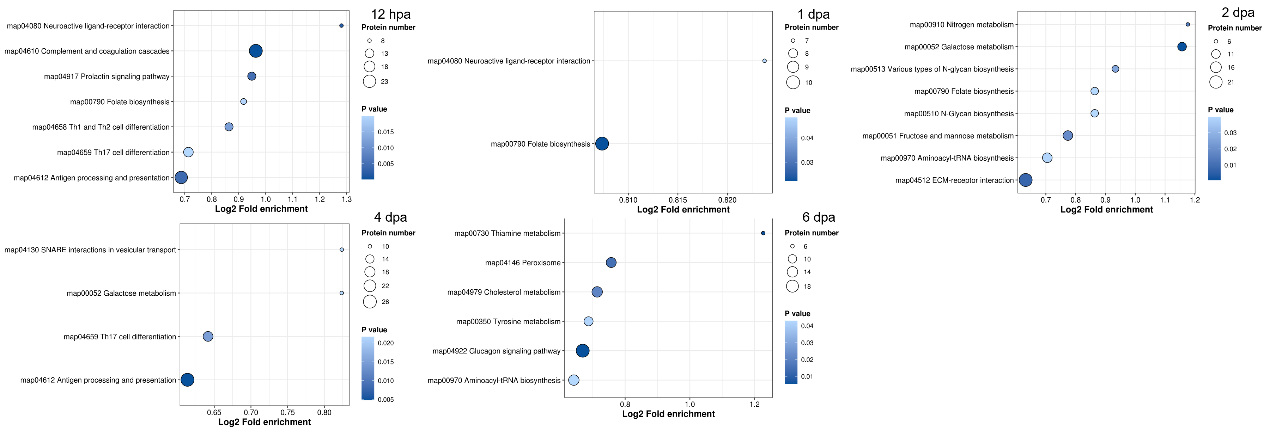


Supplementary Figure 9. Bubble map of KEGG enrichment analysis of differentially expressed proteins.


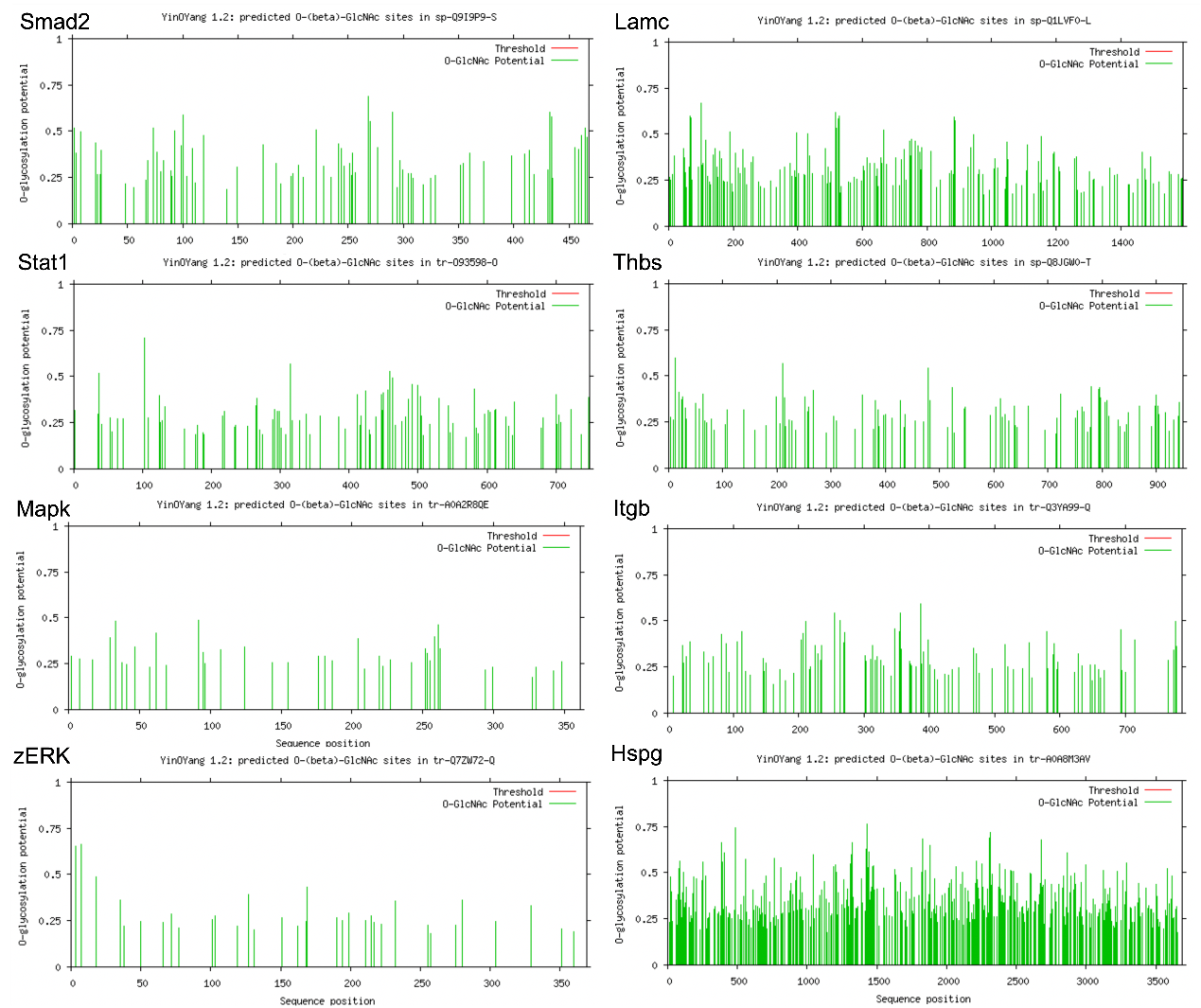
 Supplementarym Figure 10. Prediction of glycosylation sites of the differential expressed proteins.


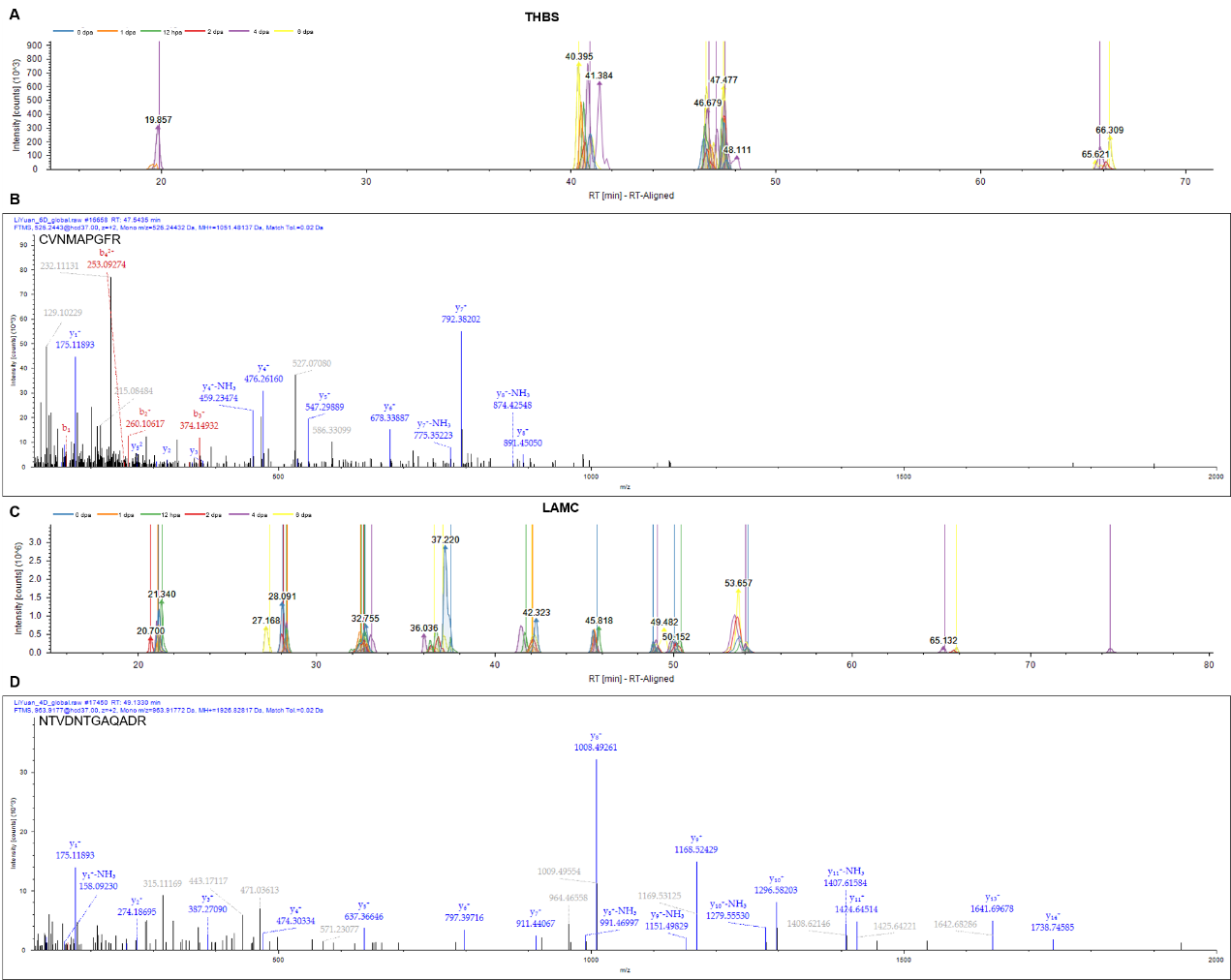


Supplementarym Figure 11. Separation and identification of O-GlcNAc Protein by LC-MS/MS. (A) The spectra of the peptides identified in THBS. (B) The annotated spectrum of the peptide CVNMAPGFR (THBS). (C) The spectra of the peptides identified in LAMC. (D) The annotated spectrum of the peptide NTVDNTGAQADR (LAMC).

**
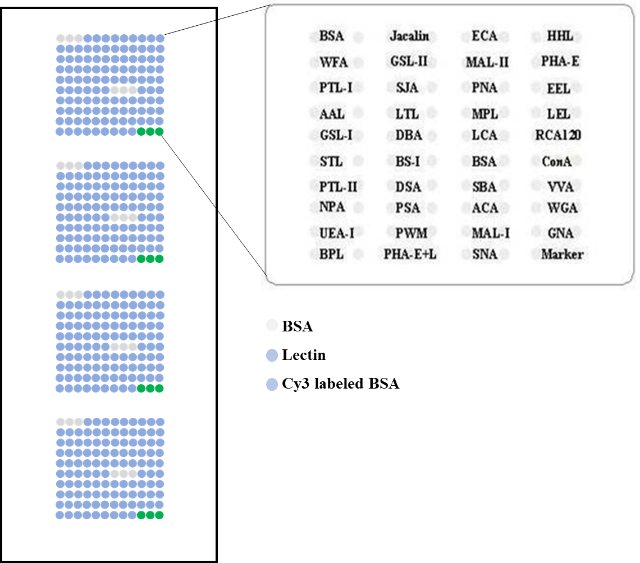
**

Supplementarym Figure 12. Lectins are carbohydrate-binding proteins that discriminate glycans based on subtle differences in structure. With the advent of high-throughput glycome techniques, the lectin microarray that enables the observation of multiple, distinct binding interactions simultaneously, becomes one of the main ways to investigate glycosylation. Briefly, 37 lectins (purchased from Vector Laboratories [Burlingame, CA], Sigma-Aldrich [St. Louis, MO], and Calbiochem [San Diego, CA]) with different binding preferences covering N- and O-linked glycans were used to produce lectin microarrays according to a previously reported protocol. The lectins were dissolved to a concentration of 1 mg/mL in the manufacturer’s recommended buffer and spotted onto homemade epoxysilane-coated slides with Stealth micro spotting pins (SMP-10B) (TeleChem, Sunnyvale, CA) by a Capital Smart Microarrayer (CapitalBio, Beijing, China). Each lectin was spotted in triplicate per block, with triplicate blocks on each slide. The microarrays were scanned with a 70% photomultiplier tube and 100% laser power settings using a Genepix 4000B confocal scanner (Axon Instruments, Union City, CA). The acquired images were analyzed at 532 nm for Cy3 detection using Genepix 3.0 software (Axon Instruments, Inc.).
