# Supplementary material for: Upregulation of Protein O-GlcNAcylation Levels Promotes Zebrafish Fin Regeneration
Source: Mol Cell Proteomics. 2025 Mar 4;24(4):100936. doi: 10.1016/j.mcpro.2025.100936 (PMC12002929; doi:10.1016/j.mcpro.2025.100936)
Supplement: Supplementary Table 1-9 [file mmc2.docx]

**Supplementary Table 1 Alteration of the glycopattern in Ctrl, 0.5dpa,1dpa, 2dpa, 4dpa and 6dpa zebrafish fin tissue**

| **Lectin** | **Specificity** | **Normalized fluorescent intensity，NFIs±SD** | | | | | | **Fold change** | | | | |
| --- | --- | --- | --- | --- | --- | --- | --- | --- | --- | --- | --- | --- |
|  |  | **Ctrl** | **0.5 dpa** | **1 dpa** | **2 dpa** | **4 dpa** | **6 dpa** | **0.5 dpa/Ctrl** | **1 dpa/Ctrl** | **2 dpa/Ctrl** | **4 dpa/Ctrl** | **6 dpa/Ctrl** |
| MAL-II | Siaα2-3Galβ1-4Glc(NAc)/Glc | 0.035±0.006 | 0.021±0.011 | 0.03±0.008 | 0.03±0.008 | 0.016±0.004 | 0.037±0.006 | 0.58 | - | 0.48 | 0.45 | - |
| EEL | Galα1-3(Fucα1-2)、Gal(blood group B antigen) | 0.046±0.004 | 0.054±0.011 | 0.043±0.008 | 0.043±0.008 | 0.072±0.012 | 0.05±0.011 | - | - | 1.64 | 1.57 | - |
| LTL | Fucα1-2Galβ1-4GlcNAc, Fucα1-3(Galβ1-4)GlcNAc | 0.024±0.003 | 0.025±0.01 | 0.025±0.002 | 0.025±0.002 | 0.021±0.004 | 0.024±0.002 | - | - | 0.63 | - | - |
| MPL | Galβ1-3GalNAc, GalNAc | 0.04±0.003 | 0.027±0.011 | 0.031±0.011 | 0.031±0.011 | 0.017±0.003 | 0.036±0.01 | - | - | 0.43 | 0.42 | - |
| RCA120 | β-Gal, Galβ-1,4GlcNAc(type II), Galβ1-3GlcNAc(type I) | 0.024±0.004 | 0.018±0.005 | 0.026±0.006 | 0.026±0.006 | 0.01±0.003 | 0.024±0.005 | - | - | 0.51 | 0.40 | - |
| STL | trimers and tetramers of GlcNAc, core (GlcNAc)of N-glycan, oligosaccharide Containing GlcNAc and MurNAc | 0.026±0.001 | 0.033±0.008 | 0.027±0.002 | 0.027±0.002 | 0.044±0.006 | 0.026±0.001 | - | - | 1.64 | 1.68 | - |
| BS-I | α-Gal, α-GalNAc, Galα-1,3Gal, Galα-1,6Glc | 0.004±0.002 | 0.004±0.004 | 0.003±0.004 | 0.003±0.004 | 0.013±0.002 | 0.002±0.002 | - | - | 4.52 | 3.50 | 0.52 |
| ConA | High-Mannose, Manα1-6(Manα1-3)Man, terminal GlcNAc | 0.042±0.008 | 0.054±0.029 | 0.044±0.011 | 0.044±0.011 | 0.066±0.011 | 0.046±0.003 | - | - | 1.59 | 1.56 | - |
| PTL-II | Gal, blood group H, T-antigen | 0.028±0.001 | 0.023±0.015 | 0.027±0.005 | 0.027±0.005 | 0.011±0.001 | 0.029±0.002 | - | - | 0.46 | 0.40 | - |
| DSA | β-D-GlcNA, (GlcNAcβ1-4)n | 0.023±0.002 | 0.019±0.018 | 0.025±0.003 | 0.025±0.003 | 0.086±0.029 | 0.024±0.003 | - | - | 2.29 | 3.82 | - |
| SBA | aα- or β-linked terminal GalNAc, (GalNAc)n, GalNAcα1-3Gal, blood-group A | 0.025±0.002 | 0.021±0.01 | 0.022±0.005 | 0.022±0.005 | 0.012±0.004 | 0.019±0.001 | - | - | 0.38 | 0.49 | - |
| NPA | High-Mannose, Manα1-6Man | 0.038±0.004 | 0.026±0.01 | 0.034±0.007 | 0.034±0.007 | 0.019±0.002 | 0.035±0.003 | - | - | 0.56 | 0.52 | - |
| WGA | Multivalent Sia and (GlcNAc)_n_ | 0.004±0.002 | 0.011±0.007 | 0.013±0.006 | 0.013±0.006 | 0.011±0.001 | 0.011±0.011 | 2.77 | 3.08 | 3.69 | 2.65 | 2.59 |
| UEA-I | Fucα1-2Galβ1-4Glc(NAc) | 0.008±0.004 | 0.007±0.005 | 0.006±0.005 | 0.006±0.005 | 0.011±0.002 | 0.003±0.002 |  | - | 1.60 | - | 0.39 |
| PHA-E+L | Bisecting GlcNAc, bi-, tri- and tetra-antennary complex-type N-glycan | 0.006±0.004 | 0.011±0.011 | 0.008±0.003 | 0.008±0.003 | 0.007±0.003 | 0.005±0.002 | 1.71 | - | - | - | - |
| SNA | Sia2-6Gal/GalNAc | 0.033±0.006 | 0.016±0.006 | 0.029±0.002 | 0.029±0.002 | 0.013±0.002 | 0.024±0.003 | 0.49 | - | 0.55 | 0.40 | - |

**Supplementary Table 2 Detailed information of O-GlcNAcylation protein (Ctrl-only)**

| **No.** | **Protein Acc No.** | **Protein name** | **Gene name** | **Coverage (%)** | **Unique Peptides** | **MW (kDa)** | **Score Sequest** |
| --- | --- | --- | --- | --- | --- | --- | --- |
| 1 | A0AUP9_DANRE | LOC571089 protein (Fragment) | tpra LOC571089 | 4 | 5 | 148.9 | 6.71 |
| 2 | NEUAB_DANRE | N-acylneuraminate cytidylyltransferase B | cmasb cmas2 | 3 | 1 | 47.6 | 2.56 |
| 3 | Q498V7_DANRE | Zgc:112414 | psph zgc:112414 | 4 | 1 | 24.9 | 2.29 |
| 4 | A0A2R8QK78_DANRE | Versican b | vcanb | 0 | 1 | 172.6 | 0 |
| 5 | F1REL9_DANRE | Laminin, beta 1b | lamb1b | 10 | 19 | 197.5 | 31.35 |
| 6 | Q6PHD6_DANRE | Integrin-linked kinase | ilk | 2 | 1 | 51 | 0 |
| 7 | A2BGB4_DANRE | Platelet-activating factor acetylhydrolase 1b, catalytic subunit 2 (Si:ch211-139a5.3) | pafah1b2 si:ch211-139a5.3 | 4 | 1 | 25.5 | 1.69 |
| 8 | F1QNM8_DANRE | Si:dkey-217f16.1 | si:dkey-217f16.1 | 1 | 1 | 189.9 | 2.55 |
| 9 | A0A0R4IQR0_DANRE | Microtubule-associated protein, RP/EB family, member 2 | mapre2 | 9 | 1 | 43.2 | 4.1 |
| 10 | G1K2H7_DANRE | Ras-related protein Rab-4B | rab4b | 10 | 1 | 23.7 | 2.36 |
| 11 | F1R008_DANRE | ARP10 actin-related protein 10 homolog | actr10 | 2 | 1 | 46 | 2.18 |
| 12 | A8WGN9_DANRE | Lipase | lipf | 2 | 1 | 44.7 | 0 |
| 13 | A0A2R8QBA1_DANRE | C-terminal-binding protein 2a | ctbp2a | 1 | 1 | 131.3 | 4.76 |
| 14 | X1WEZ3_DANRE | Cytochrome P450, family 8, subfamily B, polypeptide 2 (Fragment) | cyp8b2 | 2 | 1 | 60.5 | 2.22 |
| 15 | F1QF86_DANRE | Serine/threonine-protein phosphatase (EC 3.1.3.16) | ppp3ccb | 17 | 7 | 57.5 | 25 |
| 16 | A5PMG8_DANRE | Sortilin | sort1b | 2 | 1 | 91.5 | 2.32 |
| 17 | A0A0R4IAZ9_DANRE | SEC24 homolog C, COPII coat complex component | sec24c | 2 | 2 | 140.5 | 2.21 |
| 18 | X1WBD6_DANRE | TAP-binding protein (tapasin)-like | tapbpl | 2 | 1 | 45 | 0 |
| 19 | A0A0R4IS22_DANRE | Zgc:66475 | zgc:66475 | 2 | 1 | 60.6 | 1.71 |
| 20 | B8A4S4_DANRE | Collagen, type XVII, alpha 1a | col17a1a si:ch211-281g13.3 | 5 | 6 | 139.2 | 16.03 |
| 21 | A0A2R8QMP2_DANRE | Regulator of complement activation group 2 gene 2 | rca2.2 | 2 | 1 | 35.9 | 0 |
| 22 | A0A2R8QBL5_DANRE | Actinin, alpha 2b | actn2b | 5 | 2 | 103.3 | 4.9 |
| 23 | B8A558_DANRE | Protein phosphatase 2, regulatory subunit B', delta (Fragment) | ppp2r5d | 2 | 1 | 73.1 | 2.21 |
| 24 | B2GQ13_DANRE | Succinate dehydrogenase [ubiquinone] flavoprotein subunit, mitochondrial (EC 1.3.5.1) | sdha | 2 | 1 | 72 | 1.82 |
| 25 | A0A0G2KPJ7_DANRE | RNA-binding motif, single stranded-interacting protein 1b | rbms1b | 5 | 1 | 23.1 | 2.03 |
| 26 | Q6NYB0_DANRE | Zgc:77366 | hnrnpa0a zgc:77366 | 17 | 4 | 32 | 9.63 |
| 27 | A0A286YAF3_DANRE | Uncharacterized protein | LOC100534815 | 10 | 2 | 21 | 0 |
| 28 | AGR2_DANRE | Anterior gradient protein 2 homolog (Zagr2) | agr2 si:ch211-201m19.5 wu:fj29g05 zgc:112187 | 11 | 2 | 19.7 | 1.9 |
| 29 | Q9YH61_DANRE | Glycogen synthase kinase 3 alpha (Glycogen synthase kinase 3 alpha b) | gsk3ab GSK3 gsk3a | 8 | 2 | 48 | 9.62 |
| 30 | B1H1H9_DANRE | Pseudouridine synthase 7 (Pus7 protein) | pus7 | 5 | 3 | 71.6 | 1.82 |
| 31 | F1R9B4_DANRE | 3-hydroxybutyrate dehydrogenase, type 1 (Fragment) | bdh1 | 6 | 2 | 40.6 | 3.33 |
| 32 | F1QSR5_DANRE | Vacuolar protein-sorting-associated protein 25 | vps25 | 6 | 1 | 20.7 | 4.69 |
| 33 | Q5TZ05_DANRE | Methionine sulfoxide reductase A | msra si:dkey-221h15.1 | 4 | 1 | 26.6 | 2.74 |
| 34 | A7MCR4_DANRE | Vaccinia related kinase 1 | vrk1 | 2 | 1 | 47.6 | 0 |
| 35 | A0A0N4SU18_DANRE | Zgc:123103 (Fragment) | zgc:123103 | 7 | 3 | 40 | 1.95 |
| 36 | E7FBK8_DANRE | C1GALT1-specific chaperone 1 | c1galt1c1 | 2 | 1 | 36 | 1.87 |
| 37 | F1QKG3_DANRE | UDP glucuronosyltransferase 5 family, polypeptide F1 | ugt5f1 | 2 | 1 | 63.7 | 2.06 |
| 38 | A2RUX4_DANRE | Zgc:158327 protein | syne3 zgc:158327 | 1 | 1 | 174.8 | 2.66 |
| 39 | Q3B727_DANRE | Zgc:123289 | zgc:123289 | 40 | 1 | 41.8 | 94.11 |
| 40 | F1RDK3_DANRE | Protein lin-7 homolog | lin7b | 5 | 1 | 24.3 | 1.65 |
| 41 | A0A2R8Q696_DANRE | EMAP-like 2 | eml2 | 10 | 9 | 92 | 15.74 |
| 42 | GPD1L_DANRE | Glycerol-3-phosphate dehydrogenase 1-like protein | gpd1l | 4 | 2 | 38.3 | 3.41 |
| 43 | Q4FE55_DANRE | Ubiquitin specific protease 9 (Ubiquitin-specific peptidase 9) | usp9 | 1 | 1 | 290.1 | 2.13 |
| 44 | A0A2R8Q650_DANRE | Tropomyosin 3 | tpm3 | 25 | 1 | 33 | 14.58 |
| 45 | A0A0R4IM03_DANRE | Si:ch73-18b11.1 | si:ch73-18b11.1 | 1 | 1 | 103.9 | 1.67 |
| 46 | A8E7E7_DANRE | Intersectin 1 (SH3 domain protein) | itsn1 | 1 | 1 | 193.9 | 0 |
| 47 | A1L1F7_DANRE | Synaptogyrin | syngr2a | 5 | 1 | 24.6 | 0 |
| 48 | A0A1D5NSN6_DANRE | Zgc:175177 (Fragment) | zgc:175177 | 4 | 1 | 29.6 | 2.43 |
| 49 | A8E7F4_DANRE | MHC class I antigen ZBA transcript variant 1 (Major histocompatibility complex class I ZBA) (Zgc:64115 protein) | mhc1zba mhc1zha zgc:64115 | 3 | 1 | 45.3 | 2.01 |
| 50 | F1R7J0_DANRE | UDP-N-acetylhexosamine pyrophosphorylase-like protein 1 | uap1l1 | 6 | 2 | 56.5 | 5.64 |
| 51 | E7F741_DANRE | Si:ch211-208g24.8 | si:ch211-208g24.8 | 3 | 1 | 33.2 | 1.62 |
| 52 | A0A0R4IPT3_DANRE | Si:ch211-209n20.1 | si:ch211-209n20.1 | 2 | 1 | 73.1 | 2.04 |
| 53 | F1QG85_DANRE | Integrin beta | itgb1a | 5 | 2 | 88.6 | 5.15 |
| 54 | A0A2U9DRX9_DANRE | Caspase 7 | casp7 | 4 | 1 | 39.5 | 1.77 |
| 55 | C6ESH0_DANRE | Camel | chl1a | 1 | 1 | 129.8 | 0 |
| 56 | A8E7L0_DANRE | Annexin | anxa13l | 6 | 2 | 35.5 | 2.43 |
| 57 | A0A286Y8P7_DANRE | Mannose-1-phosphate guanyltransferase alpha-A | gmppaa | 2 | 1 | 47.6 | 2.48 |
| 58 | Q502D7_DANRE | Sepiapterin reductase a | spra spr | 23 | 1 | 28.5 | 13.7 |
| 59 | A2BGR9_DANRE | Aldehyde dehydrogenase 2 family member, tandem duplicate 1 | aldh2.1 | 10 | 1 | 56.5 | 14.65 |
| 60 | Q5RG84_DANRE | Family with sequence similarity 129, member Aa | fam129aa si:dkeyp-11g8.6 | 3 | 2 | 95.2 | 5.37 |
| 61 | A0A2R8RR95_DANRE | Arpin | arpin | 4 | 1 | 25.6 | 1.76 |
| 62 | F1QC05_DANRE | Epoxide hydrolase (EC 3.3.2.9) | ephx1 | 2 | 1 | 51.9 | 2.07 |
| 63 | A1L1T1_DANRE | Family with sequence similarity 129, member Ba (Zgc:158319) | fam129ba fam129b zgc:158319 | 1 | 1 | 95.5 | 0 |
| 64 | E7F6E2_DANRE | RNA-binding motif protein 12B | rbm12b | 2 | 2 | 78.6 | 0 |
| 65 | Q1LUX9_DANRE | Thromboxane A synthase 1 (platelet) | tbxas1 | 2 | 1 | 62.7 | 0 |
| 66 | X1WD07_DANRE | Si:ch211-210b2.3 | si:ch211-210b2.3 | 5 | 1 | 102.4 | 13.54 |
| 67 | A2BIC9_DANRE | Zgc:101679 | zgc:101679 | 3 | 1 | 35.6 | 2.04 |
| 68 | A0A2R8PX52_DANRE | Hephaestin-like 1a | hephl1a | 1 | 1 | 127.2 | 1.79 |
| 69 | A0A2R8QU83_DANRE | Si:ch211-113e8.5 | si:ch211-113e8.5 | 4 | 1 | 34.6 | 0 |
| 70 | Q1RLT7_DANRE | Advillin (Zgc:136857) | avil zgc:136857 | 1 | 1 | 90.9 | 2.2 |
| 71 | A0A0A0MPW9_DANRE | Si:ch211-250m6.2 | si:ch211-250m6.2 | 8 | 4 | 64.5 | 2.16 |
| 72 | A0A2R8QDT4_DANRE | Choline dehydrogenase (EC 1.1.99.1) | chdh | 3 | 2 | 71.9 | 3.81 |
| 73 | Q567C7_DANRE | Peroxisomal trans-2-enoyl-CoA reductase | pecr | 4 | 1 | 32.1 | 0 |
| 74 | X1WHC5_DANRE | Switching B cell complex subunit SWAP70a | swap70a | 3 | 2 | 69.2 | 2.29 |
| 75 | F1QPY7_DANRE | Small nuclear ribonucleoprotein G (snRNP-G) | snrpg | 9 | 1 | 8.4 | 2.09 |
| 76 | Q08CF9_DANRE | LOC558785 protein (Fragment) | si:dkey-73p2.1 LOC558785 | 6 | 1 | 34.9 | 4.01 |
| 77 | A7YYH0_DANRE | Zgc:65960 protein | macrod2 zgc:65960 | 14 | 5 | 50.1 | 16.41 |
| 78 | F8W4Z6_DANRE | NADPH--cytochrome P450 reductase (CPR) (P450R) (EC 1.6.2.4) | porb POR | 2 | 2 | 76.3 | 2.27 |
| 79 | A9JSV0_DANRE | Uncharacterized protein (Fragment) | tyrp1a | 2 | 1 | 64.9 | 2.72 |
| 80 | A0A0R4ITP2_DANRE | Ataxin 3 | atxn3 | 3 | 1 | 35.2 | 1.88 |
| 81 | Q8QHI1_DANRE | Sodium/potassium-transporting ATPase subunit beta | atp1b3a | 3 | 1 | 32 | 0 |
| 82 | A0A0R4IPL1_DANRE | Profilin | pfn2 | 20 | 3 | 15 | 6.93 |
| 83 | F1QYU0_DANRE | Laminin, alpha 4 | lama4 | 9 | 15 | 207.4 | 30.23 |
| 84 | A0A2R8Q8G0_DANRE | Mitochondrial fission 1 protein | fis1 | 5 | 1 | 17 | 3.06 |
| 85 | Q6P271_DANRE | Sodium/potassium-transporting ATPase subunit alpha | atp1a3a atp1a3b CH211-270K23.1-001 | 12 | 4 | 112.5 | 24.63 |
| 86 | F6PEJ8_DANRE | Sc:d0202 | sc:d0202 | 2 | 1 | 35.8 | 1.82 |
| 87 | B0UYL3_DANRE | Poly [ADP-ribose] polymerase (PARP) (EC 2.4.2.-) | si:ch211-219a4.3 | 0 | 1 | 200.9 | 0 |
| 88 | E9QIZ5_DANRE | Cadherin 26, tandem duplicate 1 | cdh26.1 | 1 | 1 | 93 | 2.1 |
| 89 | TBX6_DANRE | T-box transcription factor TBX6 (T-box protein 6) | tbx6 tbx24 | 1 | 1 | 94.9 | 0 |
| 90 | A0A0G2KLA5_DANRE | Poly [ADP-ribose] polymerase (PARP) | si:cabz01030277.1 | 1 | 2 | 153 | 2.05 |
| 91 | Q7SZY8_DANRE | Glyoxylate reductase/hydroxypyruvate reductase a (Novel protein similar to human glyoxylate reductase/hydroxypyruvate reductase (GRHPR)) | grhpra grhpr si:ch211-240l14.3 zC240L14.3-001 | 3 | 1 | 35.1 | 2.02 |
| 92 | B0S576_DANRE | Tumor protein p73 | tp73 | 2 | 1 | 71.2 | 0 |
| 93 | Q6XG62_DANRE | Protein S100 (S100 calcium-binding protein) | icn | 22 | 3 | 10.4 | 5.59 |
| 94 | F1R5A4_DANRE | Stomatin (EPB72)-like 3b | stoml3b | 11 | 1 | 31 | 2.95 |
| 95 | B0S553_DANRE | Neurofilament, medium polypeptide (Neurofilament, medium polypeptide b) | nefmb nefm DKEY-222N6.1-001 | 2 | 1 | 81.6 | 0 |
| 96 | Q6PFV1_DANRE | cAMP-regulated phosphoprotein 19a | arpp19a arpp19 | 8 | 1 | 12.4 | 2.12 |
| 97 | Q8AXX6_DANRE | Serine/threonine protein phosphatase 2A regulatory subunit | ppp2r5ea ppp2r5e2 | 4 | 1 | 54.6 | 1.65 |
| 98 | A0A2R8QIU8_DANRE | Si:dkey-283b15.4 | si:dkey-283b15.4 | 2 | 1 | 48.4 | 2.17 |
| 99 | B7ZUW2_DANRE | Coxsackie virus and adenovirus receptor (Cxadr protein) | cxadr | 5 | 2 | 40.6 | 3.71 |
| 100 | Q5U3J4_DANRE | Clathrin interactor 1 (Clathrin interactor 1a) | clint1a clint1 | 2 | 1 | 68.6 | 2.95 |
| 101 | A0A2R8QJ98_DANRE | Hexosaminidase (glycosyl hydrolase family 20, catalytic domain)-containing | hexdc | 3 | 1 | 54.8 | 1.69 |
| 102 | Q4V914_DANRE | Core histone macro-H2A | h2afy2 zgc:114051 | 12 | 6 | 39.8 | 2.16 |
| 103 | Q6JIZ2_DANRE | Pur-alpha (Pura protein) (Purine-rich element binding protein A) | puraa pura | 6 | 1 | 32.1 | 0 |
| 104 | CHM4C_DANRE | Charged multivesicular body protein 4c | chmp4c chmp4b zgc:55566 | 17 | 4 | 25.2 | 8.46 |
| 105 | AEP1_DANRE | Aerolysin-like protein | aep1 dln1 jac5 zgc:113413 | 14 | 1 | 34.3 | 7.63 |
| 106 | A4JYP8_DANRE | Cntn1a | cntn1a | 1 | 1 | 114.2 | 1.91 |
| 107 | A4JYP5_DANRE | cDNA, clone cssl:d0220 | sc:d0220 | 9 | 1 | 27.9 | 5.99 |
| 108 | F1QTF0_DANRE | 3-hydroxy-3-methylglutaryl-CoA lyase | hmgcl | 2 | 1 | 35.8 | 1.93 |
| 109 | A2BIR3_DANRE | Ubiquitin-conjugating enzyme E2L 3b | ube2l3b ube2l3l | 21 | 3 | 17.9 | 6.23 |
| 110 | F1QY52_DANRE | Si:dkey-79f11.10 (Fragment) | si:dkey-79f11.10 | 3 | 1 | 36.5 | 2.74 |
| 111 | Q08CK8_DANRE | Zgc:152960 protein | irgf4 zgc:152960 | 1 | 1 | 63.6 | 1.68 |
| 112 | A0A2R8QNU6_DANRE | Acyl-CoA thioesterase 13 | acot13 | 7 | 1 | 15.6 | 0 |
| 113 | F1RB94_DANRE | U2 small nuclear RNA auxiliary factor 1 | u2af1 | 3 | 1 | 28.4 | 1.99 |
| 114 | A0A2R8Q7A5_DANRE | START domain-containing 14 | stard14 | 3 | 1 | 30.8 | 0 |
| 115 | B3DI32_DANRE | Vcl protein | vcla vcl | 9 | 2 | 124 | 27.62 |
| 116 | CSN5_DANRE | COP9 signalosome complex subunit 5 | cops5 zgc:73130 | 2 | 1 | 37.6 | 0 |
| 117 | F1R619_DANRE | Collagen, type XVII, alpha 1b (Fragment) | col17a1b | 2 | 3 | 137.9 | 4.64 |
| 118 | Q5RGD1_DANRE | Syntaxin-binding protein 5a (tomosyn) | stxbp5a | 1 | 1 | 124.5 | 2.25 |
| 119 | E7F4G7_DANRE | Ankyrin 2a, neuronal | ank2a | 0 | 1 | 537.5 | 0 |
| 120 | A0A2R8Q2X2_DANRE | Sarcolemma-associated protein b | slmapb | 3 | 1 | 43.7 | 2.64 |
| 121 | Q6P0G2_DANRE | Zgc:77262 | zgc:77262 | 3 | 1 | 37.2 | 0 |
| 122 | Q6PBL5_DANRE | Family with sequence similarity 3, member A (Zgc:73350) | fam3a zgc:73350 | 5 | 1 | 25.1 | 2.76 |

**Supplementary Table 3 Detailed information of O-GlcNAcylation protein (0.5 dpa-only)**

| **No.** | **Protein Acc No.** | **Protein name** | **Gene name** | **Coverage (%)** | **Unique Peptides** | **MW (kDa)** | **Score Sequest** |
| --- | --- | --- | --- | --- | --- | --- | --- |
| 1 | A0A2R8RL59_DANRE | Zgc:171517 | zgc:171517 | 3 | 1 | 28.1 | 1.76 |
| 2 | F1REP3_DANRE | Metalloendopeptidase (EC 3.4.24.-) | npsn npsnl | 7 | 2 | 31.4 | 4.77 |
| 3 | A0A0R4I9N1_DANRE | Thrombospondin 4a | thbs4a | 1 | 1 | 104.7 | 0 |
| 4 | A5PN62_DANRE | Dimethylarginine dimethylaminohydrolase 2 (Si:dkey-46a12.1) | ddah2 si:dkey-46a12.1 | 5 | 1 | 31 | 2.48 |
| 5 | RA21A_DANRE | Double-strand-break repair protein rad21 homolog A (SCC1 homolog) [Cleaved into: 64-kDa C-terminal product (64-kDa carboxy-terminal product)] | rad21a rad21 SCC1 | 1 | 1 | 72.2 | 0 |
| 6 | F1QJD1_DANRE | Uncharacterized protein | apoa4b.2 apoa4b.3 | 30 | 1 | 29.3 | 17.25 |
| 7 | A0A0R4IN46_DANRE | Transglutaminase 1-like 1 (Fragment) | tgm1l1 | 1 | 1 | 90.7 | 1.87 |
| 8 | K7DY99_DANRE | Tetratricopeptide repeat domain 27 | ttc27 | 1 | 1 | 95.2 | 0 |
| 9 | A0A024B5K5_DANRE | Endothelin converting enzyme 2 (EC 3.4.24.71) (Endothelin-converting enzyme 2b) | ece2b ECE2 | 1 | 1 | 86.3 | 1.84 |
| 10 | A0A1D5NSW6_DANRE | EMAP-like 3 | eml3 | 1 | 1 | 107.8 | 0 |
| 11 | A0A0R4IUZ0_DANRE | Leucine zipper transcription factor-like 1 | lztfl1 | 3 | 1 | 34.5 | 0 |
| 12 | A0A286Y9P8_DANRE | Solute carrier family 2 (facilitated glucose transporter), member 3b | slc2a3b | 1 | 1 | 59.7 | 1.86 |
| 13 | G1K2X0_DANRE | Titin, tandem duplicate 1 | ttn.1 ttnb | 0 | 1 | 3234.3 | 1.68 |
| 14 | A2BHK1_DANRE | Si:dkey-58f10.10 | si:dkey-58f10.10 | 3 | 1 | 23.7 | 0 |
| 15 | Q3B732_DANRE | High mobility group nucleosomal-binding domain 2 | hmgn2 zgc:123210 | 17 | 1 | 7.9 | 0 |
| 16 | Q803V2_DANRE | Phosphoribosyl pyrophosphate synthetase-associated protein 2 | prpsap2 | 2 | 1 | 39.5 | 0 |
| 17 | A4IG64_DANRE | Zgc:162287 protein | exoc8 zgc:162287 | 1 | 1 | 78.7 | 1.66 |
| 18 | F1RDJ4_DANRE | SH3-domain GRB2-like endophilin B2a | sh3glb2a | 4 | 1 | 43.2 | 0 |
| 19 | F1R0E4_DANRE | Nucleoporin 50 | nup50 | 2 | 1 | 44.6 | 2.37 |
| 20 | E7F2N2_DANRE | Aminopeptidase | si:ch211-276a23.5 | 1 | 1 | 109.8 | 3.65 |
| 21 | A0A2R8PXU3_DANRE | High mobility group nucleosomal-binding domain 3 | hmgn3 | 11 | 1 | 12.9 | 2.92 |
| 22 | E7F101_DANRE | Glutamate receptor, ionotropic, N-methyl D-aspartate 1a | grin1a | 1 | 1 | 108.4 | 0 |
| 23 | A0A0R4IQ22_DANRE | Si:ch1073-110a20.7 | si:ch1073-110a20.7 | 5 | 1 | 27.9 | 0 |
| 24 | F1R108_DANRE | Integrin, alpha 3a | itga3a | 1 | 1 | 115.6 | 1.84 |
| 25 | A8WFS0_DANRE | LOC560210 protein | hsp70l LOC560210 | 11 | 2 | 70.5 | 26.57 |
| 26 | E7F368_DANRE | Protein kinase C (EC 2.7.11.13) | prkcea | 1 | 1 | 84.3 | 0 |
| 27 | A0A0R4IYU4_DANRE | Stomatin (EPB72)-like 2 | stoml2 | 3 | 1 | 39.4 | 0 |
| 28 | E7FFM8_DANRE | Zgc:92481 | zgc:92481 | 7 | 6 | 87 | 12.91 |
| 29 | F1QC76_DANRE | Matrix metallopeptidase 9 | mmp9 | 1 | 1 | 76.5 | 0 |
| 30 | F1QS22_DANRE | Ferritin | zgc:56095 | 4 | 1 | 20.4 | 0 |
| 31 | F1QBP2_DANRE | RAB14, member RAS oncogene family,-like | rab14l | 9 | 1 | 23.9 | 7.15 |
| 32 | Q503Z0_DANRE | Tpd52-like 1 | tpd52l1 zgc:110003 | 5 | 1 | 21 | 0 |
| 33 | Q6PHE3_DANRE | Si:rp71-61h23.4 protein (Fragment) | plin3 si:rp71-61h23.4 | 7 | 1 | 45.7 | 1.65 |
| 34 | E7EZG2_DANRE | Myosin IXAa | myo9aa myo9al1 | 0 | 1 | 290 | 0 |
| 35 | A0A0R4IGK8_DANRE | Transglutaminase 5,-like | tgm5l | 4 | 3 | 76 | 5.11 |
| 36 | X1WD01_DANRE | Si:ch211-210b2.4 | si:ch211-210b2.4 | 2 | 2 | 102.4 | 4.54 |
| 37 | A0A2R8QDV0_DANRE | Uncharacterized protein | emd | 10 | 2 | 20.8 | 5.14 |
| 38 | Q9DDL2_DANRE | MALT paracaspase 2 (Paracaspase) | malt2 malt1 malt1a | 2 | 1 | 90.7 | 2.48 |
| 39 | A0A0G2L836_DANRE | Nidogen 1b | nid1b | 1 | 1 | 136.7 | 2.31 |
| 40 | E7F897_DANRE | Carboxypeptidase B2 (plasma) | cpb2 | 4 | 2 | 49.1 | 2.03 |
| 41 | A0A0H4LGZ0_DANRE | Mannose receptor c type 1 | mrc1b | 1 | 2 | 163.1 | 1.66 |
| 42 | P87359_DANRE | Plasticin | prph plasticin | 6 | 2 | 51.5 | 9.66 |
| 43 | F6NMD4_DANRE | Si:ch211-288g17.4 | si:ch211-288g17.4 | 2 | 1 | 61.4 | 0 |
| 44 | A0A2R8QPL1_DANRE | TIA1 cytotoxic granule-associated RNA-binding protein | tia1 | 2 | 1 | 42.4 | 1.82 |
| 45 | B3DIZ3_DANRE | Zgc:171967 (Zgc:171967 protein) | zgc:171967 | 6 | 1 | 104.1 | 9.03 |
| 46 | M1ZMM0_DANRE | Aldehyde oxidase 5 (Aldehyde oxidase alpha) | aox5 aox3 | 1 | 1 | 147.1 | 2.04 |
| 47 | Q5RHK5_DANRE | Myotubularin-related protein 9 | mtmr9 | 2 | 1 | 62.7 | 0 |
| 48 | GPT3L_DANRE | Glycerol-3-phosphate acyltransferase 3-like | agpat9l si:ch211-155m12.4 | 2 | 1 | 50.5 | 0 |
| 49 | F1QD18_DANRE | Caspase 8-associated protein 2 | casp8ap2 | 0 | 1 | 221.8 | 0 |
| 50 | A0A2R8QDC7_DANRE | Actinin, alpha 4 | actn4 | 20 | 13 | 106.1 | 53.22 |
| 51 | F1R2L7_DANRE | Immunoglobulin light 4 variable 8 | igl4v8 | 5 | 1 | 29.5 | 0 |
| 52 | F1Q649_DANRE | Tensin 1b | tns1b tns1 | 1 | 1 | 184.9 | 0 |
| 53 | TPL2B_DANRE | Tumor necrosis factor, alpha-induced protein 8-like protein 2 B | tnfaip8l2b tnfaip8l tnfaip8l2 zgc:64074 | 4 | 1 | 21.3 | 0 |
| 54 | A0A0R4ICZ6_DANRE | Anthrax toxin receptor | antxr2a | 1 | 1 | 55.7 | 1.8 |
| 55 | A0A0R4IHJ1_DANRE | Small nuclear ribonucleoprotein polypeptide F | snrpf | 9 | 1 | 9.9 | 2.73 |
| 56 | Q58ER0_DANRE | RNA-binding motif protein 39b | rbm39b rnpc2l | 2 | 1 | 59.7 | 2.15 |
| 57 | A5PKS6_DANRE | Hapln1a protein | hapln1a | 16 | 6 | 36.8 | 12.2 |
| 58 | Q0P3Z6_DANRE | Uncharacterized protein (Fragment) |  | 4 | 1 | 29 | 2.25 |
| 59 | A0A0G2KHM6_DANRE | Peroxidasin | pxdn | 1 | 2 | 163.5 | 3.51 |
| 60 | E7FE29_DANRE | Myosin XVIIIAb | myo18ab | 0 | 1 | 286.1 | 0 |
| 61 | E7FE02_DANRE | Clustered mitochondria (cluA/CLU1) homolog b | cluhb si:ch211-274p24.5 | 1 | 1 | 160.1 | 2.16 |
| 62 | A0A0R4ISS9_DANRE | Protein C (inactivator of coagulation factors Va and VIIIa), a | proca | 2 | 1 | 48.5 | 2.31 |
| 63 | A0A0R4INK2_DANRE | UDP-N-acetylglucosamine pyrophosphorylase 1 | uap1 | 5 | 2 | 58 | 3.35 |
| 64 | E7F4T4_DANRE | Zinc finger CCCH-type-conaining 7Bb | zc3h7bb zc3h7b | 1 | 1 | 99.8 | 0 |
| 65 | Q568M0_DANRE | RAS-related 2 (Related RAS viral (R-ras) oncogene homolog 2) (Rras2 protein) | rras2 | 6 | 1 | 23.2 | 3.33 |
| 66 | A8E5B7_DANRE | NADH dehydrogenase (Ubiquinone) 1 beta subcomplex 5 (NADH:ubiquinone oxidoreductase subunit B5) | ndufb5 | 4 | 1 | 21.7 | 0 |
| 67 | Q6DRF7_DANRE | 40S ribosomal protein s11 | rps11 | 14 | 3 | 18.4 | 6.65 |
| 68 | Q568K1_DANRE | Zgc:110246 protein | pcyox1 zgc:110246 | 3 | 2 | 56.6 | 3.57 |
| 69 | B2CZB7_DANRE | Calcium-transporting ATPase | atp2b1b | 5 | 1 | 137.9 | 10.11 |
| 70 | B0R026_DANRE | Protein arginine N-methyltransferase 5 | prmt5 zC14A17.2-001 | 1 | 1 | 71.8 | 0 |
| 71 | Q6PBY8_DANRE | S-phase kinase-associated protein 1 (S-phase kinase-associated protein 1A) | skp1 skp1a | 6 | 1 | 18.7 | 3.08 |
| 72 | Q6PBT1_DANRE | 40S ribosomal protein S27 | rps27.2 zgc:73262 | 23 | 1 | 9.5 | 2.34 |
| 73 | A7YQ79_DANRE | Serpind1 protein | serpind1 | 2 | 1 | 58.1 | 2.3 |
| 74 | A0A0G2L4S8_DANRE | Jacalin 3 | jac3 si:ch211-241c24.3 | 8 | 1 | 34.6 | 7.41 |
| 75 | F1QLV3_DANRE | Poly [ADP-ribose] polymerase (PARP) | parp2 | 1 | 1 | 73.1 | 0 |
| 76 | A0A2R8QB14_DANRE | Uncharacterized protein |  | 0 | 1 | 217.3 | 0 |
| 77 | F1QXF4_DANRE | THO complex 2 | thoc2 | 1 | 1 | 178.3 | 0 |

**Supplementary Table 4 Detailed information of O-GlcNAcylation protein (1 dpa-only)**

| **No.** | **Protein Acc No.** | **Protein name** | **Gene name** | **Coverage (%)** | **Unique Peptides** | **MW (kDa)** | **Score Sequest** |
| --- | --- | --- | --- | --- | --- | --- | --- |
| 1 | X1WHH2_DANRE | Si:dkey-159n16.2 (Fragment) | si:dkey-159n16.2 | 6 | 3 | 139.5 | 11.4 |
| 2 | R4GE14_DANRE | Si:ch211-207i1.2 | si:ch211-207i1.2 | 0 | 1 | 997.3 | 2.1 |
| 3 | F1QNE7_DANRE | Trafficking protein particle complex 4 | trappc4 | 4 | 1 | 24.5 | 0 |
| 4 | A0A0G2KGL2_DANRE | Phosphoribosylglycinamide formyltransferase | gart | 3 | 1 | 108.5 | 3.92 |
| 5 | A0A2R8QI77_DANRE | Periaxin | prx | 0 | 1 | 445.1 | 0 |
| 6 | E7FF26_DANRE | Extended synaptotagmin-like protein 2a | esyt2a | 1 | 1 | 98.9 | 2.26 |
| 7 | A0A2R8RWP8_DANRE | Peptidylprolyl isomerase | fkbp5 | 3 | 1 | 53.4 | 2.13 |
| 8 | Q6TNT6_DANRE | U4/U6-associated RNA splicing factor | prpf3 HPRP3P | 2 | 1 | 78.8 | 2.1 |
| 9 | A0A0R4IY81_DANRE | NLR family, CARD domain-containing 3-like | nlrc3l | 1 | 1 | 93.7 | 1.98 |
| 10 | A0A385NIA3_DANRE | Carbonic anhydrase II | ca2 | 12 | 3 | 28.9 | 6.05 |
| 11 | E9QDE9_DANRE | RAB GTPase-activating protein 1-like | rabgap1l | 1 | 1 | 115.5 | 2.64 |
| 12 | X1WHB9_DANRE | Zgc:114181 | zgc:114181 | 2 | 1 | 54.5 | 2.38 |
| 13 | A0A0B4J1B0_DANRE | Insulin-like growth factor 2 mRNA-binding protein 2a | igf2bp2a | 2 | 1 | 66.5 | 2.44 |
| 14 | A7MBR5_DANRE | Nop58 protein | nop58 nol5 | 3 | 1 | 62 | 0 |
| 15 | JGN1B_DANRE | Protein jagunal homolog 1-B | jagn1b si:ch73-162i18.3 zgc:101045 | 7 | 1 | 20.7 | 0 |
| 16 | F1QH49_DANRE | Cyclin G-associated kinase | gak | 1 | 1 | 146.4 | 2.55 |
| 17 | A0A2R8Q6T7_DANRE | Dihydropyrimidinase like 2 | dpysl2a | 2 | 1 | 62.9 | 3.17 |
| 18 | F1QLE3_DANRE | Zgc:153722 | zgc:153722 | 4 | 1 | 32.6 | 2.55 |
| 19 | Q802Y1_DANRE | Serine and arginine-rich-splicing factor 4 | srsf4 sfrs4 zgc:55809 | 2 | 1 | 41.6 | 2.07 |
| 20 | E7FCW1_DANRE | Wu:fk65c09 | wu:fk65c09 | 4 | 2 | 56.9 | 4.38 |
| 21 | Q7T3G4_DANRE | Phosphoglycerate mutase | pgam2 | 4 | 1 | 28.8 | 5.6 |
| 22 | A0A0R4IP12_DANRE | PDS5 cohesin-associated factor B | pds5b | 1 | 1 | 159.7 | 0 |
| 23 | Q08CE0_DANRE | Si:dkey-42i9.6 protein | si:dkey-42i9.6 | 3 | 1 | 27.9 | 0 |
| 24 | B0R118_DANRE | Protein kinase C and casein kinase substrate in neurons 3 | pacsin3 | 3 | 1 | 50.6 | 0 |
| 25 | A0A2R8Q718_DANRE | 26S proteasome non-ATPase regulatory subunit 1 | psmd1 | 5 | 6 | 110.4 | 10.7 |
| 26 | A0A0R4IG81_DANRE | Calpain 1, (mu/I) large subunit b | capn1b | 3 | 1 | 80.1 | 4.84 |
| 27 | A0A0R4IFV9_DANRE | Receptor protein-tyrosine kinase (EC 2.7.10.1) | egfra | 1 | 1 | 132.4 | 1.8 |
| 28 | A9JR96_DANRE | WD repeat domain 43 | wdr43 wdr43l | 1 | 1 | 71.6 | 0 |
| 29 | F1RBY1_DANRE | A kinase (PRKA) anchor protein 1b | akap1b | 2 | 1 | 89.8 | 1.94 |
| 30 | F8W4V6_DANRE | G-rich RNA sequence-binding factor 1 | grsf1 | 2 | 1 | 46 | 1.64 |
| 31 | A5PMG4_DANRE | Adenosylhomocysteinase | ahcyl1 CH211-199O1.3-001 | 4 | 1 | 61.3 | 0 |
| 32 | F1QN57_DANRE | Si:dkey-145c18.3 (Fragment) | si:dkey-145c18.3 | 3 | 2 | 79.9 | 1.94 |
| 33 | Q6DHP3_DANRE | ADP-ribosylation factor 3a | arf3a | 4 | 1 | 20.6 | 2.45 |
| 34 | F1QZ57_DANRE | Poly-U-binding-splicing factor a | puf60a | 1 | 1 | 59.3 | 0 |
| 35 | F1QQU8_DANRE | Protein BCCIP homolog | bccip | 3 | 1 | 34 | 2.24 |
| 36 | TSN31_DANRE | Tetraspanin-31 (Tspan-31) | tspan31 sas zgc:56710 | 4 | 1 | 23.3 | 1.72 |
| 37 | Q66IF0_DANRE | Dehydrogenase/reductase (SDR family) member 13a, tandem duplicate 2 (Zgc:101565) | dhrs13a.2 zgc:101565 | 7 | 2 | 34.8 | 0 |
| 38 | RPZ_DANRE | Protein rapunzel | rpz | 4 | 1 | 26.6 | 2.56 |
| 39 | CNO11_DANRE | CCR4-NOT transcription complex subunit 11 | cnot11 zgc:163002 | 2 | 1 | 49.7 | 0 |
| 40 | Q5TYY2_DANRE | Exocyst complex component 4 | exoc4 | 1 | 1 | 109.6 | 2.32 |
| 41 | Q9DEY8_DANRE | Cytoskeleton-associated LIM domain protein | lima1a eplin lima1 | 6 | 4 | 70 | 8.89 |
| 42 | F1QV31_DANRE | Keratin 5 | krt5 | 44 | 11 | 59.1 | 116.03 |
| 43 | F1QJ31_DANRE | Uncharacterized protein | iws1 | 1 | 1 | 86.6 | 1.83 |
| 44 | ERLN1_DANRE | Erlin-1 (Endoplasmic reticulum lipid raft-associated protein 1) | erlin1 si:ch211-223p8.2 zgc:110547 | 10 | 4 | 38.3 | 5.2 |
| 45 | Q6PH40_DANRE | Major histocompatibility complex class I UXA2 protein | mhc1uga mhc1uxa2 | 3 | 1 | 39.6 | 1.87 |
| 46 | F1QIQ2_DANRE | BCL2-associated athanogene 6 | bag6 | 1 | 1 | 128.1 | 2.22 |
| 47 | B8A552_DANRE | SWI/SNF-related, matrix-associated, actin-dependent regulator of chromatin, subfamily a, member 5 | smarca5 | 2 | 2 | 120.5 | 4.04 |
| 48 | Q642H6_DANRE | Carnitine O-acetyltransferase a (Zgc:92317) | crata zgc:92317 | 2 | 1 | 71.2 | 1.61 |
| 49 | F1RCB2_DANRE | DEAD-box helicase 3 X-linked b | ddx3xb ddx3b pl10 | 5 | 3 | 75.8 | 8.3 |
| 50 | A0A0R4IAT9_DANRE | Stromal interaction molecule 1b | stim1b | 3 | 2 | 75.6 | 2.43 |
| 51 | E7F3G8_DANRE | Si:ch211-113j14.1 | si:ch211-113j14.1 | 2 | 1 | 66 | 2.06 |
| 52 | Q4QRI0_DANRE | Adh8a protein (Alcohol dehydrogenase 8a) | adh8a | 2 | 1 | 40.6 | 2.04 |
| 53 | A0A0R4ILC0_DANRE | Polymerase (DNA-directed), delta-interacting protein 3 (Fragment) | poldip3 | 3 | 1 | 51.4 | 0 |
| 54 | Q7ZTY5_DANRE | Eukaryotic translation initiation factor 2B, subunit 3 gamma | eif2b3 | 3 | 1 | 50.5 | 0 |
| 55 | B1H1M2_DANRE | Zgc:63611 protein | aqr zgc:63611 | 1 | 2 | 176.8 | 2 |
| 56 | Q7ZW85_DANRE | DnaJ (Hsp40) homolog, subfamily C, member 5 gamma a | dnajc5ga dnajc5g zgc:56703 | 5 | 1 | 22.3 | 1.86 |
| 57 | F1Q7P7_DANRE | Pdgfa-associated protein 1b | pdap1b pdap1 | 6 | 1 | 19.9 | 2.71 |
| 58 | Q561X8_DANRE | Proteasome subunit beta (EC 3.4.25.1) | psmb8f zgc:110346 | 3 | 1 | 30.6 | 0 |
| 59 | A0A286Y9F1_DANRE | Clathrin light chain | cltb | 7 | 2 | 25.3 | 2.05 |
| 60 | A0A2R8RKV0_DANRE | TNF receptor-associated protein 1 | trap1 | 2 | 1 | 88.7 | 1.71 |
| 61 | Q803Z3_DANRE | Exportin 6 | xpo6 | 1 | 1 | 128.3 | 0 |
| 62 | A0A2R8QIY5_DANRE | Protein kinase C (EC 2.7.11.13) | prkcaa prkca | 1 | 1 | 76.2 | 0 |
| 63 | F1RBF6_DANRE | 14-3-3 protein beta/alpha-B | ywhabb | 32 | 2 | 27.4 | 31.98 |
| 64 | F1QBH0_DANRE | Olfactomedin-like protein 3A | olfml3a | 2 | 1 | 44.9 | 0 |
| 65 | Q5RGX4_DANRE | Thymopoietin a | tmpoa tmpo | 1 | 1 | 75.3 | 2 |
| 66 | E7F533_DANRE | Eph receptor A2 b | epha2b | 2 | 1 | 108.9 | 3.94 |
| 67 | A0A0G2LAI5_DANRE | Uncharacterized protein |  | 3 | 2 | 62.1 | 1.83 |
| 68 | A2RV44_DANRE | Zgc:158846 protein | zgc:158846 | 12 | 1 | 55.2 | 21.84 |
| 69 | F1QKC0_DANRE | SMU1, DNA replication regulator and spliceosomal factor a | smu1a smu1 | 3 | 1 | 57.5 | 0 |
| 70 | E7F4M2_DANRE | Alpha thalassemia/mental retardation syndrome X-linked homolog (human) | atrx | 0 | 1 | 227.6 | 0 |
| 71 | Q90XR8_DANRE | Claudin b (Cldnb protein) | cldnb | 6 | 1 | 22.6 | 2.47 |
| 72 | A1A5V2_DANRE | Zgc:158252 | palm3 zgc:158252 | 2 | 1 | 47.5 | 2.24 |
| 73 | B7ZUU8_DANRE | Adaptor-related protein complex 3 subunit delta 1 | ap3d1 si:ch211-129c21.6 | 1 | 1 | 140.1 | 1.62 |
| 74 | A0A2R8QR37_DANRE | Uncharacterized protein |  | 5 | 1 | 19.5 | 0 |
| 75 | B3DG87_DANRE | Numb homolog (Drosophila) | numb | 1 | 1 | 73 | 2.64 |
| 76 | A0A0R4IWR3_DANRE | DnaJ homolog subfamily C member 21 | dnajc21 | 2 | 1 | 62.8 | 0 |
| 77 | A7MCK6_DANRE | Small ubiquitin-related modifier (SUMO) | sumo1 | 9 | 1 | 11.4 | 2.1 |
| 78 | MBB1A_DANRE | Myb-binding protein 1A-like protein | mybbp1a | 1 | 1 | 143.9 | 0 |
| 79 | CP062_DANRE | UPF0505 protein C16orf62 homolog | zgc:163107 | 1 | 1 | 108.6 | 1.79 |
| 80 | Q8JHD9_DANRE | Casein kinase 1, alpha 1 (Casein kinase 1-alphaL) | csnk1a1 | 8 | 1 | 40.5 | 1.85 |
| 81 | K9L5F8_DANRE | SEL1L | sel1l | 1 | 1 | 85.8 | 1.61 |
| 82 | Q6DG11_DANRE | Spliceosome-associated factor 1, recruiter of U4/U6.U5 tri-snRNP (Squamous cell carcinoma antigen recognised by T cells) | sart1 | 1 | 1 | 88.8 | 2 |
| 83 | E7F0R9_DANRE | SIN3 transcription regulator family member Ab | sin3ab | 1 | 1 | 144 | 2.49 |
| 84 | IMPA3_DANRE | Inositol monophosphatase 3 (IMP 3) (IMPase 3) (EC 3.1.3.25) | impad1 impa3 zgc:123256 | 3 | 1 | 37.4 | 2 |
| 85 | B2GRK9_DANRE | Tkt protein | tktb tkt | 7 | 5 | 68 | 15.11 |
| 86 | Q6NZ14_DANRE | Tyrosine-protein phosphatase non-receptor type 12 (EC 3.1.3.48) | ptpn12 | 2 | 1 | 64.7 | 2.02 |
| 87 | F1Q4X5_DANRE | Complement component 4 | c4 | 1 | 1 | 190.9 | 2.4 |
| 88 | A0A2R8Q7C7_DANRE | Cadherin 26, tandem duplicate 1 | cdh26.1 | 1 | 1 | 97.7 | 2.17 |
| 89 | F8W411_DANRE | RAD50 homolog, double strand break repair protein | rad50 | 1 | 2 | 153.6 | 1.97 |
| 90 | F1QU24_DANRE | ADAM metallopeptidase domain 9 | adam9 | 2 | 2 | 90.2 | 2.41 |
| 91 | B8JIV0_DANRE | RAP2A, member of RAS oncogene family b | rap2ab | 6 | 1 | 20.7 | 1.93 |
| 92 | F8W428_DANRE | Cullin 4B | cul4b | 1 | 1 | 99.4 | 1.99 |
| 93 | M10B1_DANRE | Putative helicase mov-10-B.1 (EC 3.6.4.13) | mov10b.1 si:dkeyp-38g6.2 | 1 | 1 | 116.3 | 2.24 |
| 94 | Q803F3_DANRE | Pdip5 protein (Protein disulfide isomerase-related protein (Provisional)) | pdia6 pdip5 | 6 | 2 | 48 | 6.71 |
| 95 | A0A0R4IEA2_DANRE | Exocyst complex component | exoc6b | 1 | 1 | 92.3 | 0 |
| 96 | A0A0R4IRU6_DANRE | Rho GTPase-activating protein 24 | arhgap24 | 1 | 1 | 84.1 | 0 |
| 97 | Q5XJJ8_DANRE | Adaptor-related protein complex 3, sigma 1 subunit | ap3s1 | 6 | 1 | 21.7 | 2.55 |
| 98 | A0A0R4IK69_DANRE | RAB11-binding protein RELCH homolog | relch | 2 | 2 | 133 | 1.63 |
| 99 | A0A2R8QIF2_DANRE | Exocyst complex component 3 | exoc3 | 1 | 1 | 86.9 | 1.88 |
| 100 | I3ISA6_DANRE | Epiplakin 1 | eppk1 | 4 | 2 | 611.3 | 4.6 |
| 101 | X1WCH5_DANRE | Zinc finger protein 839 | znf839 | 1 | 1 | 107.2 | 1.62 |
| 102 | F1QZK7_DANRE | FinTRIM family, member 36 | ftr36 | 4 | 3 | 75.4 | 4.36 |
| 103 | Q9PVF7_DANRE | Cell-adhesion protein plakoglobin (Junction plakoglobin) | jupa jup | 15 | 10 | 80 | 25.48 |
| 104 | A1L1T9_DANRE | ArfGAP with SH3 domain, ankyrin repeat and PH domain 2b | asap2b ddef2 ddef2b | 2 | 2 | 113.6 | 0 |
| 105 | Q6PFK8_DANRE | Zgc:66306 | nup160 zgc:66306 | 1 | 1 | 135 | 3.29 |
| 106 | Q6PBJ3_DANRE | VAMP/synaptobrevin (Vesicle-associated membrane protein 1) | vamp1 | 6 | 1 | 13 | 1.76 |
| 107 | F1Q554_DANRE | Kinesin family member 14 | kif14 | 0 | 1 | 168.5 | 0 |
| 108 | F1Q932_DANRE | Cortactin | cttn | 1 | 1 | 60.3 | 1.78 |
| 109 | F1QNY5_DANRE | Copine I | cpne1 | 8 | 4 | 59.2 | 6.6 |
| 110 | A0A0R4IYD0_DANRE | 40S ribosomal protein S27 | rps27l | 10 | 1 | 9.5 | 2.58 |
| 111 | A9JRG9_DANRE | AFG3-like AAA ATPase 2 (Si:ch211-12e1.4 protein) | afg3l2 si:ch211-12e1.4 | 2 | 1 | 88.6 | 1.87 |

**Supplementary Table 5 Detailed information of O-GlcNAcylation protein (2 dpa-only)**

| **No.** | **Protein Acc No.** | **Protein name** | **Gene name** | **Coverage (%)** | **Unique Peptides** | **MW (kDa)** | **Score Sequest** |
| --- | --- | --- | --- | --- | --- | --- | --- |
| 1 | F1QJE0_DANRE | Ubiquinol-cytochrome c reductase core protein 2a | uqcrc2a | 2 | 1 | 49.2 | 2.2 |
| 2 | Q1MTE6_DANRE | Sideroflexin | sfxn2 CH211-208D15.3-001 | 5 | 2 | 35.6 | 1.71 |
| 3 | A2BGL0_DANRE | Poly(A) polymerase gamma | papolg | 2 | 1 | 83 | 3.05 |
| 4 | A0A0R4IBL4_DANRE | DCN1-like protein (Defective in cullin neddylation protein 1-like protein) | dcun1d1 | 4 | 1 | 32.7 | 1.69 |
| 5 | E7FD05_DANRE | Si:dkey-7j14.6 | si:dkey-7j14.6 | 8 | 2 | 24.2 | 0 |
| 6 | A0A0R4IC31_DANRE | A kinase (PRKA) anchor protein 12b | akap12b | 1 | 1 | 172.1 | 1.75 |
| 7 | WDR11_DANRE | WD repeat-containing protein 11 | wdr11 | 1 | 1 | 137.1 | 2.3 |
| 8 | F1QJB8_DANRE | GTPase-activating protein (SH3 domain)-binding protein 1 (Fragment) | g3bp1 | 17 | 1 | 39.1 | 17.52 |
| 9 | F1QJB3_DANRE | Si:ch1073-280e3.1 | si:ch1073-280e3.1 | 1 | 1 | 92.9 | 2 |
| 10 | A0A0B4J1A5_DANRE | EWS RNA-binding protein 1a | ewsr1a | 1 | 1 | 64.9 | 0 |
| 11 | Q6IQM9_DANRE | Ribonuclease (EC 3.1.26.4) | rnaseh2a | 4 | 1 | 34.2 | 1.92 |
| 12 | R4GE93_DANRE | Si:zfos-2330d3.7 (Fragment) | si:zfos-2330d3.7 | 4 | 1 | 28.2 | 2.06 |
| 13 | A0A0R4IMP0_DANRE | U2 snRNP auxiliary factor large subunit | u2af2b | 2 | 1 | 54.2 | 0 |
| 14 | A3QJW5_DANRE | CTP synthase (EC 6.3.4.2) (UTP--ammonia ligase) | ctps1a | 5 | 3 | 67 | 2.64 |
| 15 | A3KMS7_DANRE | Snw1 protein | snw1 | 3 | 1 | 63.6 | 3.03 |
| 16 | Q6IMW9_DANRE | LSM6 homolog, U6 small nuclear RNA and mRNA degradation-associated (LSM6 homolog, U6 small nuclear RNA associated (S. cerevisiae)) (Lsm6 protein) | lsm6 | 14 | 1 | 9.1 | 2.34 |
| 17 | A0A2R8Q2N0_DANRE | 10-formyltetrahydrofolate dehydrogenase (EC 1.5.1.6) | aldh1l1 | 2 | 1 | 100.1 | 4.53 |
| 18 | A5PMG2_DANRE | Si:ch211-199o1.2 | si:ch211-199o1.2 | 13 | 1 | 70.6 | 20.77 |
| 19 | B0V0U4_DANRE | Polypeptide N-acetylgalactosaminyltransferase (EC 2.4.1.-) (Protein-UDP acetylgalactosaminyltransferase) | galnt2 si:dkey-162b3.1 | 2 | 1 | 63.9 | 1.67 |
| 20 | F1QST7_DANRE | Retinoblastoma-binding protein 5 | rbbp5 | 2 | 1 | 56.4 | 1.9 |
| 21 | A5WV16_DANRE | Acyl-CoA synthetase long chain family member 4b | acsl4b | 6 | 4 | 73.5 | 8.5 |
| 22 | B6IDE7_DANRE | Fras1-related extracellular matrix protein 2b (Frem2b) | frem2b | 0 | 1 | 347.5 | 2.85 |
| 23 | Q4VBH5_DANRE | Gna13a protein (Guanine nucleotide binding protein (G protein), alpha 13a) | gna13a | 12 | 3 | 43.8 | 9.25 |
| 24 | A5PMI3_DANRE | NOP2/Sun RNA methyltransferase family, member 2 | nsun2 | 2 | 2 | 86.7 | 0 |
| 25 | A0JMK0_DANRE | Periostin, osteoblast specific factor | postnb postn | 17 | 1 | 82.9 | 33.88 |
| 26 | F1QAR9_DANRE | Alkaline phosphatase (EC 3.1.3.1) | alpi.1 alpi | 4 | 1 | 58.3 | 2.77 |
| 27 | A8E546_DANRE | Interferon regulatory factor 10 | irf10 | 2 | 1 | 44.4 | 1.74 |
| 28 | A0A0G2KTT4_DANRE | Si:dkey-167k11.5 | si:dkey-167k11.5 | 5 | 1 | 26.2 | 0 |
| 29 | F6NNU9_DANRE | Si:ch211-114l13.3 | si:ch211-114l13.3 | 7 | 5 | 120.7 | 20.4 |
| 30 | A0A2R8QN75_DANRE | Nuclear receptor subfamily 3, group C, member 1 | nr3c1 | 1 | 1 | 81.9 | 2.4 |
| 31 | A0A2R8QBR5_DANRE | Solute carrier family 39 member 11 |  | 6 | 1 | 33.1 | 3.35 |
| 32 | Q7ZVX9_DANRE | Thioredoxin domain-containing 9 (Txndc9 protein) | txndc9 | 5 | 1 | 26.1 | 2.87 |
| 33 | Q568V5_DANRE | Uroporphyrinogen decarboxylase (EC 4.1.1.37) (Fragment) | urod | 2 | 1 | 45.2 | 1.8 |
| 34 | A0A2R8Q6F8_DANRE | E3 ubiquitin-protein ligase TRIP12 | trip12 | 0 | 1 | 232.3 | 0 |
| 35 | A0A0R4INC0_DANRE | EMAP-like 1 | eml1 | 1 | 1 | 92.7 | 0 |
| 36 | Q7ZVX1_DANRE | Na(+)/H(+) exchange regulatory cofactor NHE-RF | slc9a3r1a slc9a3r1 slc9a3r2 | 3 | 1 | 41.4 | 1.82 |
| 37 | GALC_DANRE | Galactocerebrosidase (GALCERase) (EC 3.2.1.46) | galc galca si:ch211-199l3.4 zgc:92561 | 2 | 1 | 73.8 | 3.24 |
| 38 | A0A158VDR9_DANRE | C1qA | c1qa | 4 | 1 | 26.1 | 2.72 |
| 39 | A5WUM0_DANRE | Non-specific serine/threonine protein kinase (EC 2.7.11.1) | prkaa1 DKEY-226M8.8-001 | 3 | 2 | 65 | 2.22 |
| 40 | A0A2R8QMX8_DANRE | Protein tyrosine phosphatase, receptor type, f, b | ptprfb | 1 | 1 | 213.9 | 2.04 |
| 41 | F6NVK8_DANRE | Cullin 3a | cul3a cul3b | 2 | 1 | 89.8 | 3.96 |
| 42 | Q502M3_DANRE | LOC553451 protein (Fragment) | sptbn1 LOC553451 | 12 | 1 | 113.8 | 26.82 |
| 43 | Q7ZZ04_DANRE | Enoyl CoA hydratase, short chain, 1, mitochondrial | echs1 | 4 | 1 | 31.2 | 1.65 |
| 44 | A0A2R8Q5W8_DANRE | Elongation factor Ts, mitochondrial (EF-Ts) (EF-TsMt) | tsfm TSFM | 4 | 2 | 33.4 | 0 |
| 45 | Q5KTT6_DANRE | eIF4G-related protein NAT1B | eif4g2b NAT1B | 4 | 4 | 102.2 | 7.69 |
| 46 | Q6Q419_DANRE | Ribosomal protein S29 | rps29 | 13 | 1 | 6.7 | 0 |
| 47 | Q6PE45_DANRE | Protein RER1 | rer1 RER1 | 5 | 1 | 22.7 | 0 |
| 48 | A0A2R8QMB8_DANRE | Coiled-coil domain-containing protein 47 | ccdc47 | 2 | 1 | 60.1 | 0 |
| 49 | F1RDL0_DANRE | Protein phosphatase 1 regulatory subunit 21 | ppp1r21 | 3 | 2 | 75.7 | 1.78 |
| 50 | Q7ZV59_DANRE | Synaptojanin 2-binding protein (Zgc:56207) | synj2bp zgc:56207 | 8 | 1 | 16.1 | 2.72 |
| 51 | SELS_DANRE | Selenoprotein S (SelS) (VCP-interacting membrane protein) | vimp sels zgc:136970 | 9 | 1 | 21.2 | 3.31 |
| 52 | F1R0Q1_DANRE | Solute carrier family 2 (facilitated glucose transporter), member 1b | slc2a1b | 3 | 2 | 53.6 | 1.83 |
| 53 | A5X6X5_DANRE | Titin a | ttn.2 ttna | 0 | 2 | 3646.1 | 0 |
| 54 | E7F6V6_DANRE | Si:dkeyp-27c8.2 | si:dkeyp-27c8.2 | 11 | 2 | 18.4 | 0 |
| 55 | F1RA35_DANRE | Microtubule-actin crosslinking factor 1a | macf1a | 0 | 1 | 652.1 | 0 |
| 56 | Q7ZUL8_DANRE | Zgc:55558 (Zgc:55558 protein) | zgc:55558 | 13 | 2 | 21.4 | 4.28 |
| 57 | F1RA25_DANRE | Chitinase, acidic.4 | chia.4 | 4 | 2 | 52.7 | 0 |
| 58 | F1R3T4_DANRE | Galactokinase 1 | galk1 | 3 | 1 | 42.3 | 2.42 |
| 59 | F1QFY1_DANRE | Cullin 5b | cul5b | 1 | 1 | 92.2 | 0 |
| 60 | E7EYW5_DANRE | Cystatin 14a, tandem duplicate 1 | cst14a.1 | 12 | 1 | 11.2 | 2.81 |
| 61 | Q504F5_DANRE | Calpain 2, (M/II) large subunit b (Capn2b protein) | capn2b | 18 | 13 | 78.2 | 46 |
| 62 | F1RCY6_DANRE | UPF1, RNA helicase and ATPase | upf1 | 2 | 2 | 122 | 2.26 |
| 63 | F1RCY7_DANRE | RNA-binding motif protein 15 | rbm15 | 1 | 1 | 94.9 | 0 |
| 64 | B0JZP5_DANRE | Deoxyribonuclease II, lysosomal (Dnase2 protein) | dnase2 | 2 | 1 | 40.7 | 0 |
| 65 | Q0PWB8_DANRE | PDZ and LIM domain 3b (PDZ-LIM protein ALP) | pdlim3b pdlim3 DKEY-276L17.2-001 | 5 | 2 | 34.7 | 0 |
| 66 | A0A2R8QPR3_DANRE | Deoxyuridine triphosphatase | dut | 5 | 1 | 20.2 | 0 |
| 67 | A5WVI9_DANRE | DnaJ (Hsp40) homolog, subfamily A, member 2, like | dnaja2b dnaja2l | 6 | 1 | 45.6 | 4.52 |
| 68 | A0A0R4IYB7_DANRE | N(alpha)-acetyltransferase 25, NatB auxiliary subunit | naa25 | 1 | 1 | 111.6 | 1.71 |
| 69 | A0A0R4IBS8_DANRE | Sorting nexin 7 | snx7 | 2 | 1 | 54.1 | 0 |
| 70 | A1L273_DANRE | Zgc:158680 | tmed1b zgc:158680 | 3 | 1 | 26 | 1.67 |
| 71 | A0A2R8Q9T2_DANRE | Calcium uptake protein 1, mitochondrial | micu1 | 2 | 1 | 56.4 | 1.85 |
| 72 | E9QFE1_DANRE | Myosin, heavy chain 14, non-muscle | myh14 | 4 | 2 | 233.2 | 13.56 |
| 73 | Q5RFW4_DANRE | Protein-L-isoaspartate O-methyltransferase (EC 2.1.1.77) | pcmt | 6 | 1 | 28.6 | 2.16 |
| 74 | E7F2B6_DANRE | Poly (ADP-ribose) glycohydrolase a | parga | 3 | 2 | 86.9 | 0 |
| 75 | F1QZN3_DANRE | Si:ch211-212c13.10 | si:ch211-212c13.10 | 7 | 1 | 160.1 | 26.31 |
| 76 | A2BGN0_DANRE | Metabolism of cobalamin-associated B | mmab si:dkey-189p24.5 | 4 | 1 | 25.9 | 0 |
| 77 | Q1RLR0_DANRE | Cryl1 protein (Fragment) | cryl1 im:6895749 | 3 | 1 | 36.4 | 2.07 |
| 78 | Q803N5_DANRE | TAR DNA-binding protein,-like (Tardbpl protein) | tardbpl | 13 | 1 | 34.3 | 7.62 |
| 79 | Q6P6E2_DANRE | 40S ribosomal protein S26 | rps26 | 16 | 2 | 13 | 4.7 |
| 80 | Q4U0S6_DANRE | N-ethylmaleimide-sensitive factor b | nsfb | 10 | 1 | 82.7 | 8.88 |
| 81 | UHRF1_DANRE | E3 ubiquitin-protein ligase UHRF1 (EC 2.3.2.27) (RING-type E3 ubiquitin transferase UHRF1) | uhrf1 | 2 | 1 | 87 | 2.6 |
| 82 | A0A2R8RZT8_DANRE | UDP-N-acetylhexosamine pyrophosphorylase-like protein 1 | uap1l1 | 6 | 2 | 56.3 | 9.57 |
| 83 | E9QJQ5_DANRE | Si:dkeyp-110e4.6 | pld7 si:dkeyp-110e4.6 | 1 | 1 | 86.3 | 1.93 |
| 84 | Q7T3L7_DANRE | Cell division control protein 2 (Cell division cycle 2) (Cyclin-dependent kinase 1) | cdk1 cdc2 | 7 | 1 | 34.5 | 5.33 |
| 85 | A0A0R4IHV1_DANRE | Pyruvate kinase (EC 2.7.1.40) | pkmb | 6 | 2 | 58.3 | 4.66 |
| 86 | X1WD79_DANRE | TAO kinase 2a | taok2a | 1 | 1 | 129.5 | 2.15 |
| 87 | B2GSC5_DANRE | Eukaryotic translation initiation factor 2A (eIF-2A) | eif2a zgc:110460 | 4 | 2 | 64.2 | 2.33 |
| 88 | A0A0R4IHT0_DANRE | Solute carrier family 25 (aspartate/glutamate carrier), member 12 | slc25a12 | 3 | 2 | 75.3 | 4.6 |
| 89 | M9MMA4_DANRE | Immunoglobulin superfamily, member 3 | igsf3 | 2 | 2 | 116.9 | 2.03 |
| 90 | B0S5U2_DANRE | E3 ubiquitin-protein ligase TRIM33 | trim33 | 1 | 1 | 129.4 | 2.67 |
| 91 | F1RCN0_DANRE | DNA (cytosine-5)-methyltransferase (EC 2.1.1.37) | dnmt1 | 3 | 1 | 169 | 3.9 |
| 92 | Q08CA5_DANRE | Phosphoribosyl pyrophosphate synthetase 1B (Prps1b protein) | prps1b | 4 | 1 | 34.8 | 2.27 |
| 93 | TIM13_DANRE | Mitochondrial import inner membrane translocase subunit Tim13 | timm13 tim13a timm13a zgc:92895 | 11 | 1 | 10.6 | 0 |
| 94 | Q7T3D5_DANRE | Programmed cell death 6 | pdcd6 | 5 | 1 | 21.9 | 0 |
| 95 | A4QNX5_DANRE | Ubiquitin-conjugating enzyme E2D 4 (putative) | ube2d4 zgc:162263 | 7 | 1 | 16.7 | 2.01 |
| 96 | A9JT20_DANRE | Zgc:101030 protein | arf4a zgc:101030 | 14 | 1 | 20.3 | 4.23 |
| 97 | A5D6U5_DANRE | Taxilin alpha (Zgc:162882 protein) | txlna zgc:162882 | 4 | 2 | 58.7 | 1.76 |
| 98 | EXOS9_DANRE | Exosome complex component RRP45 (Exosome component 9) | exosc9 | 2 | 1 | 44.1 | 0 |
| 99 | A0A0R4IY13_DANRE | Si:ch1073-416d2.3 | si:ch1073-416d2.3 | 2 | 1 | 50.1 | 0 |
| 100 | U6BNE9_DANRE | MHC class I antigen ZKA transcript variant 1 (Major histocompatibility complex class I ZKA) | mhc1zka | 7 | 1 | 43.8 | 6.97 |
| 101 | A0A0A0MPL4_DANRE | Syntaxin 7-like | stx7l | 5 | 1 | 28.9 | 2.36 |
| 102 | F1QZ64_DANRE | ATP-binding cassette, sub-family F (GCN20), member 2a | abcf2a | 6 | 4 | 70 | 7.13 |
| 103 | A0A2R8QTF0_DANRE | Cold shock domain-containing E1, RNA-binding | csde1 | 2 | 1 | 96.6 | 3.25 |
| 104 | A0A2R8PWG5_DANRE | PRP6 pre-mRNA-processing factor 6 homolog (S. cerevisiae) | prpf6 | 2 | 2 | 109.3 | 0 |
| 105 | A0A0G2L3U3_DANRE | Nucleus accumbens-associated 1, BEN and BTB (POZ) domain-containing a | nacc1a | 1 | 1 | 58.4 | 0 |
| 106 | F1Q5Q7_DANRE | Exosome component 10 | exosc10 | 1 | 1 | 102.3 | 0 |
| 107 | E9QBZ4_DANRE | Fermitin family member 1 | fermt1 si:ch73-22c10.1 | 2 | 1 | 76 | 2.35 |
| 108 | A9JTB6_DANRE | Serine (Or cysteine) proteinase inhibitor, clade D (Heparin cofactor), member 1 | serpind1 | 1 | 1 | 58.1 | 1.64 |
| 109 | A9JTC4_DANRE | Mediator of cell motility 1 (Protein MEMO1) | memo1 | 2 | 1 | 33.4 | 0 |
| 110 | T179B_DANRE | Transmembrane protein 179B | tmem179b zgc:110591 | 5 | 1 | 24.5 | 1.95 |
| 111 | A0A2R8RYU6_DANRE | Cleft lip and palate transmembrane protein 1 homolog | clptm1 | 1 | 1 | 73.1 | 1.77 |
| 112 | E7F3D6_DANRE | SCY1-like, kinase-like 1 | scyl1 si:ch211-276i12.7 | 2 | 2 | 94.2 | 1.85 |
| 113 | A0A2R8RNA7_DANRE | SH3-domain GRB2-like endophilin B1b | sh3glb1b | 4 | 1 | 40.3 | 1.76 |
| 114 | A0A0D5X166_DANRE | LGP2 variant 1 | dhx58 | 3 | 2 | 77.7 | 2.81 |
| 115 | A0A2U9DQK9_DANRE | Caspase 8a | casp8 | 4 | 2 | 55 | 4.73 |
| 116 | F8W298_DANRE | Zgc:174259 | zgc:174259 | 18 | 1 | 46.7 | 14 |
| 117 | Q5RGR6_DANRE | Wiskott-Aldrich syndrome-like b | waslb | 5 | 3 | 55.6 | 1.98 |
| 118 | Q801U3_DANRE | Serine and arginine-rich-splicing factor 3a (Splicing factor, arginine/serine-rich 3a) | srsf3a sfrs3 sfrs3a | 9 | 2 | 20.3 | 3.82 |
| 119 | EF1A_DANRE | Elongation factor 1-alpha (EF-1-alpha) | eef1a ef1a | 31 | 19 | 50 | 78.23 |
| 120 | Q4V984_DANRE | RAB1B, member RAS oncogene family b (Zgc:86773 protein) | rab1bb zgc:86773 | 33 | 1 | 22.5 | 20.3 |
| 121 | A0A2R8RTM3_DANRE | Paralemmin 1b | palm1b | 3 | 1 | 27.2 | 0 |
| 122 | Q6DG42_DANRE | Non-specific serine/threonine protein kinase (EC 2.7.11.1) | pak2a pak2 | 21 | 11 | 57.3 | 25.49 |
| 123 | Q5CZM4_DANRE | Glycoprotein M6Bb (Gpm6bb protein) | gpm6bb | 3 | 1 | 29.5 | 1.72 |
| 124 | A4QP95_DANRE | LOC563828 protein (Fragment) | si:ch1073-280e3.1 LOC563828 | 1 | 1 | 93.2 | 1.71 |
| 125 | Q6IQ59_DANRE | Ubiquinol-cytochrome c reductase core protein 2b (Ubiquinol-cytochrome c reductase core protein II) | uqcrc2b uqcrc2 | 11 | 4 | 48.1 | 7.76 |
| 126 | Q7SYE1_DANRE | Zgc:63524 | canx zgc:63524 | 17 | 10 | 67.8 | 25.56 |
| 127 | A2VD22_DANRE | Metalloendopeptidase (EC 3.4.24.-) | npsn npsnl | 3 | 1 | 30.6 | 0 |
| 128 | F1RC25_DANRE | Transient receptor potential melastatin 4b1 | trpm4b.2 TRPM4b1 trpm4c | 1 | 1 | 139.4 | 1.76 |
| 129 | Q6DRE5_DANRE | NOP56 (NOP56 ribonucleoprotein homolog) | nop56 nol5a | 2 | 1 | 60.6 | 2.3 |
| 130 | Q6DG15_DANRE | Zgc:92392 | gnal2 zgc:92392 | 5 | 1 | 46.8 | 5.14 |
| 131 | A4FVI7_DANRE | LSM7 homolog, U6 small nuclear RNA and mRNA degradation-associated (LSM7 homolog, U6 small nuclear RNA associated (S. cerevisiae)) | lsm7 | 8 | 1 | 11.6 | 0 |
| 132 | E7F981_DANRE | Nucleoporin 98 | nup98 | 1 | 1 | 195.7 | 1.83 |
| 133 | A0A0R4IA93_DANRE | Cytoskeleton-associated protein 5 | ckap5 | 1 | 1 | 225.2 | 0 |
| 134 | F1R142_DANRE | Zgc:153284 (Fragment) | zgc:153284 | 9 | 1 | 16.3 | 0 |
| 135 | Q6DRF4_DANRE | Replication factor C subunit RFC4 | rfc4 | 3 | 1 | 39.9 | 0 |
| 136 | B0S5G8_DANRE | Wiskott-Aldrich syndrome (eczema-thrombocytopenia) b | wasb | 2 | 1 | 51.6 | 0 |
| 137 | A0A2R8RJS5_DANRE | Fragile X mental retardation, autosomal homolog 2 | fxr2 | 1 | 1 | 75.4 | 1.73 |
| 138 | A0A0R4IK82_DANRE | Si:ch211-233m11.2 | si:ch211-233m11.2 | 1 | 1 | 102.6 | 2.05 |
| 139 | F1Q8I4_DANRE | Tyrosine-protein phosphatase non-receptor type (EC 3.1.3.48) | ptpn2a | 3 | 1 | 45.3 | 0 |
| 140 | PEF1_DANRE | Peflin (PEF protein with a long N-terminal hydrophobic domain) (Penta-EF hand domain-containing protein 1) | pef1 zgc:100787 | 8 | 2 | 29.4 | 2.64 |
| 141 | Q1LXK8_DANRE | Novel protein containing an Acyl-CoA dehydrogenase, N-terminal and C-terminal domain (Fragment) | CH211-262K23.4-001 zgc:85777 | 2 | 1 | 49.2 | 2.31 |
| 142 | Q6DRM9_DANRE | Structural maintenance of chromosomes protein | smc1al smc1a smc1l1 | 13 | 17 | 143.1 | 42.03 |
| 143 | A0A2R8S0I4_DANRE | Achaete-scute homolog 1b | ascl1b | 3 | 3 | 100.6 | 3.76 |
| 144 | F1QE30_DANRE | V-type proton ATPase subunit a | atp6v0a1b | 1 | 1 | 95.6 | 1.95 |
| 145 | Q05AQ0_DANRE | Zgc:152682 | mhc2dab zgc:152682 | 8 | 1 | 28.2 | 6.87 |
| 146 | Q6IQB1_DANRE | Ribosomal protein L5b | rpl5b | 16 | 4 | 34 | 10.1 |
| 147 | A0A2R8Q3E9_DANRE | Sp2 transcription factor | sp2 | 1 | 1 | 64.7 | 1.8 |
| 148 | Q6IQE6_DANRE | Zgc:86798 | ralbb zgc:86798 | 13 | 1 | 23.3 | 4.44 |
| 149 | B0JZB9_DANRE | Sart3 protein | sart3 | 1 | 1 | 109.8 | 0 |
| 150 | Q6PC13_DANRE | Prohibitin 2 (Prohibitin 2a) | phb2a phb2 | 4 | 1 | 33.3 | 1.96 |
| 151 | A9UH06_DANRE | Muscleblind-like protein 1D (Muscleblind-like-splicing regulator 1) | mbnl1 mbnl1d | 2 | 1 | 45.1 | 0 |
| 152 | Q6IQF8_DANRE | Peptidyl-prolyl cis-trans isomerase (PPIase) (EC 5.2.1.8) | ppih | 8 | 2 | 23.8 | 3.85 |
| 153 | F1Q8P0_DANRE | MHC class I antigen ZFA transcript variant 1 (Major histocompatibility complex class I ZFA) | mhc1zfa | 6 | 1 | 43.4 | 6.84 |
| 154 | Q6TH07_DANRE | Flotillin 1 (Flotillin 1b) | flot1b FLOT1 | 2 | 1 | 47.2 | 2.22 |
| 155 | A0A0R4INZ8_DANRE | Dihydrolipoamide acetyltransferase component of pyruvate dehydrogenase complex (EC 2.3.1.-) (Fragment) | dbt | 2 | 1 | 58.4 | 0 |
| 156 | A0A0R4IAH0_DANRE | Histone deacetylase (EC 3.5.1.98) | hdac4 | 2 | 2 | 116.4 | 0 |

**Supplementary Table 6 Detailed information of O-GlcNAcylation protein (4 dpa-only)**

| **No.** | **Protein Acc No.** | **Protein name** | **Gene name** | **Coverage (%)** | **Unique Peptides** | **MW (kDa)** | **Score Sequest** |
| --- | --- | --- | --- | --- | --- | --- | --- |
| 1 | F1R4C8_DANRE | NADH:ubiquinone oxidoreductase core subunit S3 | ndufs3 | 5 | 1 | 29.5 | 1.86 |
| 2 | F1QNB1_DANRE | Ankyrin 3a | ank3a | 0 | 1 | 455.3 | 2.59 |
| 3 | B3DHC5_DANRE | Apolipoprotein A-IV b, tandem duplicate 2 (Zgc:194131) | apoa4b.2 zgc:194131 | 35 | 8 | 29.4 | 25.27 |
| 4 | Q6NUY4_DANRE | Trafficking protein particle complex 6b-like (Trappc6bl protein) | trappc6bl | 5 | 1 | 18.4 | 0 |
| 5 | Q503S3_DANRE | Golgi phosphoprotein 3 (Golph3 protein) | golph3 | 7 | 1 | 33.6 | 2.39 |
| 6 | F1QJC9_DANRE | Collagen, type I, alpha 1a | col1a1a | 3 | 3 | 136.8 | 6.76 |
| 7 | A0A0R4IML0_DANRE | Capping protein (actin filament), gelsolin-like b | capgb | 46 | 16 | 38.6 | 44.47 |
| 8 | A0A0R4IQR2_DANRE | Protein-tyrosine sulfotransferase (EC 2.8.2.20) | tpst1 | 3 | 1 | 42.2 | 0 |
| 9 | A8KBL3_DANRE | Kctd12.2 protein (Fragment) | kctd12.2 | 4 | 1 | 32 | 1.9 |
| 10 | NIPS2_DANRE | Protein NipSnap homolog 2 (NipSnap2) | nipsnap2 gbas | 3 | 1 | 33.6 | 0 |
| 11 | A0A2R8PZB2_DANRE | Si:dkey-156n14.5 | si:dkey-156n14.5 | 2 | 1 | 75.6 | 2.78 |
| 12 | E7EY65_DANRE | Metastasis-associated 1 family, member 3 | mta3 | 1 | 1 | 80 | 2.14 |
| 13 | F1QT86_DANRE | Collagen, type V, alpha 2a (Fragment) | col5a2a | 1 | 1 | 147.3 | 1.86 |
| 14 | A0A0G2KQ62_DANRE | Fibrillin 2b | fbn2b | 0 | 1 | 310 | 1.78 |
| 15 | Q6P3H9_DANRE | Solute carrier family 3 (amino acid transporter heavy chain), member 2b (Zgc:55813) | slc3a2b zgc:55813 | 2 | 1 | 56 | 1.7 |
| 16 | A2BG19_DANRE | Novel protein similar to vertebrate skeletal alpha-actin 1 | actc1c | 31 | 1 | 42 | 115.07 |
| 17 | E7FGC6_DANRE | Uncharacterized protein | LOC799279 | 2 | 1 | 62.4 | 1.83 |
| 18 | A0A0R4IQ83_DANRE | Si:ch211-156b7.4 | si:ch211-156b7.4 | 7 | 1 | 19.7 | 1.62 |
| 19 | Q2TV65_DANRE | Superoxide dismutase (EC 1.15.1.1) | sod2 | 6 | 2 | 25 | 0 |
| 20 | F1QKG7_DANRE | Tropomyosin 4a | tpm4a | 5 | 1 | 32.7 | 3.4 |
| 21 | F1Q731_DANRE | COP9 signalosome complex subunit 3 | cops3 | 3 | 1 | 48.6 | 2.83 |
| 22 | Q7ZV25_DANRE | Tubulin-specific chaperone A | tbca | 6 | 1 | 12.6 | 1.6 |
| 23 | Q7ZUR5_DANRE | Signal sequence receptor, gamma | ssr3 | 8 | 1 | 21.1 | 2.53 |
| 24 | A0A2R8PY75_DANRE | Isocitrate dehydrogenase [NAD] subunit, mitochondrial | idh3b | 4 | 1 | 41.9 | 2.86 |
| 25 | Q6GQL6_DANRE | Dynactin 3 (P22) | dctn3 | 6 | 1 | 21.7 | 2.98 |
| 26 | G5CTT5_DANRE | Polynucleotide phosphorylase |  | 2 | 1 | 85 | 2.9 |
| 27 | Q803S1_DANRE | SWI/SNF-related, matrix-associated, actin-dependent regulator of chromatin, subfamily e, member 1 (Smarce1 protein) | smarce1 | 2 | 1 | 46.9 | 0 |
| 28 | A0A0R4ICR8_DANRE | Basigin | bsg | 7 | 2 | 43.9 | 1.74 |
| 29 | F1R7L3_DANRE | Bystin-like (Fragment) | bysl | 4 | 2 | 53.1 | 1.7 |
| 30 | A2RV05_DANRE | LOC571625 protein (Fragment) | ncapd2 LOC571625 | 1 | 2 | 157.8 | 3.56 |
| 31 | Q1JPY6_DANRE | Prefoldin subunit 2 | pfdn2 | 15 | 2 | 17.1 | 3.13 |
| 32 | A0A2R8QGU6_DANRE | Striatin, calmodulin-binding protein 4 | strn4 | 1 | 1 | 86.3 | 0 |
| 33 | Q5XJ23_DANRE | Dut protein (dUTP pyrophosphatase) | dut | 5 | 1 | 20.1 | 2.36 |
| 34 | E7F143_DANRE | Charged multivesicular body protein 5a | chmp5a | 3 | 1 | 28.5 | 0 |
| 35 | A7MCN0_DANRE | Peptidyl-prolyl cis-trans isomerase (PPIase) (EC 5.2.1.8) | ppifb zgc:123307 | 16 | 1 | 20.8 | 9.78 |
| 36 | E7FBP9_DANRE | Cancer susceptibility candidate 4 | casc4 | 2 | 1 | 55.6 | 1.85 |
| 37 | KC15L_DANRE | BTB/POZ domain-Containing protein kctd15-like | kctd15l zgc:103747 | 4 | 1 | 29.3 | 2.81 |
| 38 | A9JRT4_DANRE | Ubiquitin-Conjugating enzyme E2D 3 (Zgc:56340 protein) | ube2d3 zgc:56340 | 7 | 1 | 16.7 | 2.2 |
| 39 | Q5SPD1_DANRE | Elongation factor-1, delta, b (Eukaryotic translation elongation factor 1 delta b (guanine nucleotide exchange protein)) | eef1db si:dkey-235d18.4 | 16 | 2 | 30.3 | 9.25 |
| 40 | A5PLB5_DANRE | LOC559122 protein (Fragment) | cfhl4 LOC559122 | 2 | 1 | 53.4 | 2.36 |
| 41 | A7MCF4_DANRE | Zgc:101710 protein (Fragment) | ech1 zgc:101710 | 4 | 1 | 34.2 | 2.09 |
| 42 | F1QRX2_DANRE | Small ubiquitin-related modifier (SUMO) | sumo3b | 27 | 2 | 10.7 | 3.96 |
| 43 | B2GSP4_DANRE | Ubiquitin-like modifier activating enzyme 3 | uba3 | 2 | 1 | 51.6 | 1.68 |
| 44 | Q1LVA8_DANRE | Acyl-CoA synthetase long chain family member 3a | acsl3a | 2 | 1 | 79 | 3.11 |
| 45 | E3NZJ1_DANRE | Plexin B2b long isoform | plxnb2b | 1 | 2 | 207.4 | 0 |
| 46 | A1L1V2_DANRE | Matrix-remodelling associated 8 (Si:ch211-15p9.5) | mxra8a | 2 | 1 | 51.3 | 2.63 |
| 47 | F1Q6K1_DANRE | Ubiquitin-specific peptidase 24 | usp24 | 0 | 1 | 294.7 | 1.74 |
| 48 | PRRC1_DANRE | Protein PRRC1 (Proline-rich and coiled-coil-Containing protein 1) | prrc1 mys zgc:103484 | 4 | 2 | 45.4 | 3.43 |
| 49 | Q66I20_DANRE | Serpin peptidase inhibitor, clade F (alpha-2 antiplasmin, pigment epithelium-derived factor), member 1 (Zgc:92072) | serpinf1 zgc:92072 | 5 | 2 | 44.9 | 0 |
| 50 | A8WGC1_DANRE | UDP-glucuronosyltransferase (EC 2.4.1.17) | zgc:172315 | 2 | 1 | 60.8 | 2.26 |
| 51 | A0A2R8QGF4_DANRE | Si:ch211-1a19.3 | si:ch211-1a19.3 | 6 | 1 | 27.7 | 0 |
| 52 | F8W5H1_DANRE | Myotubularin 1 | mtm1 | 1 | 1 | 73.5 | 0 |
| 53 | A0A0R4IHU9_DANRE | Glutaredoxin 3 | glrx3 | 15 | 1 | 36.3 | 8.37 |
| 54 | A0A0P0CMM0_DANRE | CARMIL1 | carmil1 lrrc16a | 1 | 2 | 163.4 | 0 |
| 55 | Q9DDG4_DANRE | Replication protein A | rpa2 rpa | 4 | 1 | 29.6 | 2.84 |
| 56 | Q5SPS1_DANRE | Family with sequence similarity 184, member A | fam184a | 1 | 1 | 133.8 | 1.9 |
| 57 | HSDL1_DANRE | Inactive hydroxysteroid dehydrogenase-like protein 1 | hsdl1 si:ch211-172b19.1 zgc:103498 | 3 | 1 | 35.1 | 0 |
| 58 | A3KP76_DANRE | Zgc:175088 protein (Fragment) | zgc:175088 | 6 | 1 | 21 | 0 |
| 59 | A9JT23_DANRE | Dehydrogenase/reductase (SDR family) member 13b | dhrs13b zgc:91936 | 2 | 1 | 37.6 | 0 |
| 60 | A5D6S1_DANRE | Im:7150662 protein | im:7150662 | 3 | 1 | 60.4 | 0 |
| 61 | F1Q5F3_DANRE | Lamin B1 | lmnb1 | 18 | 6 | 68.3 | 25.73 |
| 62 | F1QCK2_DANRE | ATP-binding cassette, sub-family B (MDR/TAP), member 6a | abcb6a abcb6 | 1 | 1 | 96.5 | 0 |
| 63 | A3KP18_DANRE | Zgc:171352 protein (Fragment) | habp2 zgc:171352 | 3 | 1 | 62.4 | 1.88 |
| 64 | TMCO1_DANRE | Calcium load-activated calcium channel (CLAC channel) (Transmembrane and coiled-coil domain-Containing protein 1) | tmco1 | 5 | 1 | 21.3 | 2.12 |
| 65 | F1R6R2_DANRE | Si:ch211-270n8.1 (Fragment) | si:ch211-270n8.1 | 14 | 2 | 29 | 3.32 |
| 66 | Q6P5J7_DANRE | Chloride intracellular channel 3 (Chloride intracellular channel a) | clic3 clica | 3 | 1 | 27.2 | 1.67 |
| 67 | F6NM51_DANRE | Ubiquitin-associated protein 2-like | ubap2l | 1 | 1 | 123.9 | 2.68 |
| 68 | E9QBZ2_DANRE | Ankyrin repeat domain 28b | ankrd28b | 1 | 1 | 112.9 | 0 |
| 69 | RHOAD_DANRE | Rho-related GTP-binding protein RhoA-D | rhoad | 22 | 1 | 22 | 12.19 |
| 70 | F1QYK7_DANRE | Protein kinase C-binding protein 1,-like | prkcbp1l | 1 | 1 | 124 | 1.64 |
| 71 | A0A2R8Q8P2_DANRE | Lysine-specific histone demethylase (EC 1.-.-.-) | kdm1a | 2 | 2 | 94.7 | 0 |
| 72 | A9JTF1_DANRE | Protein quaking-A (Quaking) | qkia qk | 2 | 1 | 42.2 | 1.86 |
| 73 | Q6DHS8_DANRE | Testis derived transcript (3 LIM domains) | tes | 10 | 4 | 57.2 | 9.47 |
| 74 | F1QIB2_DANRE | Cleavage stimulation factor, 3' pre-RNA, subunit 3 | cstf3 | 2 | 1 | 82.7 | 2.92 |
| 75 | A0A1L1QZG0_DANRE | Chaperonin-Containing TCP1, subunit 2 (beta) (Fragment) | cct2 | 40 | 1 | 11.2 | 10.17 |
| 76 | A3KQB2_DANRE | Eukaryotic translation initiation factor 4, gamma 2b | eif4g2b | 2 | 2 | 102.2 | 3.91 |
| 77 | A0A2R8QJ70_DANRE | SET nuclear proto-oncogene b | setb | 5 | 1 | 33.2 | 0 |
| 78 | Q6P0B3_DANRE | Secretory carrier-associated membrane protein (Secretory carrier membrane protein) | scamp1 zgc:77214 | 4 | 1 | 37.6 | 2.84 |
| 79 | G0ZJH8_DANRE | UBE4B-III splice isoform III | ube4b Ube4B | 1 | 1 | 151.6 | 3.14 |
| 80 | Q4V942_DANRE | Zgc:56304 (Zgc:56304 protein) | zgc:56304 | 2 | 1 | 55 | 1.65 |
| 81 | Q6DRE6_DANRE | 60S ribosomal protein L12 (Ribosomal protein L12) | rpl12 | 55 | 7 | 17.7 | 19.14 |
| 82 | A0A0R4IA89_DANRE | Si:dkey-27n6.1 | si:dkey-27n6.1 | 4 | 0 | 119.4 | 9.09 |
| 83 | Q6PC41_DANRE | Histone deacetylase complex subunit SAP18 | sap18 | 5 | 1 | 17.5 | 1.69 |
| 84 | A0A0R4IS45_DANRE | Conserved oligomeric Golgi complex subunit 6 (COG complex subunit 6) (Component of oligomeric Golgi complex 6) | cog6 | 1 | 1 | 73.4 | 0 |
| 85 | F1R1N9_DANRE | CD2-associated protein | cd2ap cd2apl | 1 | 1 | 72.8 | 1.66 |
| 86 | Q1L8E2_DANRE | Peptidase (mitochondrial-processing) beta | pmpcb | 4 | 1 | 52.3 | 2.48 |
| 87 | Q6ZM12_DANRE | Hemoglobin, beta adult 2 (Novel beta globin) | hbba2 | 26 | 4 | 16.3 | 8.08 |
| 88 | B0V1K1_DANRE | Aminoacyl tRNA synthetase complex-interacting multifunctional protein 1 | aimp1 | 3 | 1 | 34.5 | 1.74 |
| 89 | Q6P0G5_DANRE | Tfg protein (Trk-fused gene) | tfg | 3 | 1 | 42.3 | 0 |
| 90 | A9C3W5_DANRE | Si:dkey-159f12.2 (Fragment) | si:dkey-159f12.2 | 6 | 1 | 19.8 | 2.08 |
| 91 | A0A0R4IDW8_DANRE | Si:dkey-193i10.4 | si:dkey-193i10.4 | 5 | 3 | 135.8 | 11.53 |
| 92 | Q6IQH4_DANRE | Proteasome 26S subunit, non-ATPase 8 (Zgc:86762) | psmd8 zgc:86762 | 16 | 4 | 31 | 8.04 |
| 93 | Q75T39_DANRE | Neurocan (Fragment) | ncana cspg3 cspg3l ncan ncanl | 11 | 1 | 11.2 | 1.83 |
| 94 | A0A0R4IAH3_DANRE | Enoyl-CoA delta isomerase 2 | eci2 | 4 | 3 | 43 | 1.91 |
| 95 | A0A2R8RKC7_DANRE | Elongation factor 1-alpha | eef1a2 | 17 | 1 | 50.7 | 24.47 |

**Supplementary Table 7 Detailed information of O-GlcNAcylation protein (6 dpa-only)**

| **No.** | **Protein Acc No.** | **Protein name** | **Gene name** | **Coverage (%)** | **Unique Peptides** | **MW (kDa)** | **Score Sequest** |
| --- | --- | --- | --- | --- | --- | --- | --- |
| 1 | A0A2R8QJZ7_DANRE | Uncharacterized protein | rab31 | 6 | 1 | 21.8 | 0 |
| 2 | ARHL2_DANRE | ADP-ribose glycohydrolase ARH3 (ADP-ribosylhydrolase 3) (O-acetyl-ADP-ribose deacetylase ARH3) (EC 3.5.1.-) | adprhl2 arh3 zgc:92867 | 2 | 1 | 40.1 | 0 |
| 3 | A0A0A0VB08_DANRE | NADH-ubiquinone oxidoreductase chain 1 (EC 7.1.1.2) | ND1 | 3 | 1 | 35.7 | 0 |
| 4 | Q6IQP4_DANRE | RAP1A, member of RAS oncogene family (RAP1A, member of RAS oncogene family a) | rap1aa rap1a | 16 | 1 | 21 | 8.08 |
| 5 | COL12_DANRE | Collectin-12 | colec12 si:ch211-212m21.5 | 4 | 3 | 78 | 2.02 |
| 6 | A0A2R8Q1X8_DANRE | Vacuolar protein sorting-associated protein 26B | vps26b | 5 | 1 | 39.9 | 2.68 |
| 7 | A8WGK7_DANRE | Elongation factor 1-alpha | eef1a1b zgc:110335 | 18 | 1 | 50.3 | 31.31 |
| 8 | F1R8J6_DANRE | Palmitoyl-protein thioesterase 1 (ceroid-lipofuscinosis, neuronal 1, infantile) | ppt1 | 4 | 1 | 33.9 | 2.43 |
| 9 | E7F1J4_DANRE | DENN/MADD domain-Containing 5A | dennd5a | 1 | 1 | 147.3 | 0 |
| 10 | A0A0G2KTI4_DANRE | Solute carrier family 12 (sodium/potassium/chloride transporter), member 2 | slc12a2 | 1 | 1 | 124 | 1.64 |
| 11 | F1QRY4_DANRE | TIP41, TOR-signaling pathway regulator-like (S. cerevisiae) | tiprl | 3 | 1 | 31 | 2.38 |
| 12 | A8KBL8_DANRE | UDP-glucuronosyltransferase (EC 2.4.1.17) | zgc:112491 zgc:112490 | 10 | 3 | 61 | 2.61 |
| 13 | A0A0R4IBI6_DANRE | Peptidylprolyl isomerase (EC 5.2.1.8) | fkbp10a | 2 | 1 | 63 | 0 |
| 14 | A0A2R8PV02_DANRE | Beta-hexosaminidase (EC 3.2.1.52) | hexa | 2 | 1 | 60.8 | 0 |
| 15 | A0A0R4IBG2_DANRE | Acid phosphatase 1 | acp1 | 6 | 1 | 17.7 | 2.25 |
| 16 | Q4VBU0_DANRE | 2-oxoisovalerate dehydrogenase subunit alpha | bckdha | 2 | 1 | 50.7 | 2.75 |
| 17 | A3KMT0_DANRE | Zgc:158803 (Zgc:158803 protein) | zgc:158803 | 2 | 1 | 50.1 | 0 |
| 18 | Q6IMW8_DANRE | Ribosomal protein L39 (Zgc:92371) | rpl39 zgc:92371 | 20 | 1 | 6.4 | 2.7 |
| 19 | GT251_DANRE | Procollagen galactosyltransferase 1 (EC 2.4.1.50) (Collagen beta(1-O)galactosyltransferase 1) | colgalt1 glt25d1 si:ch211-114l13.7 zgc:110667 | 3 | 1 | 70.8 | 1.96 |
| 20 | F1R8S0_DANRE | DR1-associated protein 1 (negative cofactor 2 alpha) | drap1 | 5 | 1 | 23 | 2.14 |
| 21 | F8W2M8_DANRE | Tripeptidyl peptidase I | tpp1 | 2 | 1 | 64.3 | 3.51 |
| 22 | A0A0R4INA6_DANRE | Gephyrin a | gphna | 1 | 1 | 94.2 | 0 |
| 23 | A8KBQ8_DANRE | Rab proteins geranylgeranyltransferase component A | chm | 2 | 1 | 74 | 1.83 |
| 24 | A8KBQ9_DANRE | Zgc:56068 protein | gde1 zgc:56068 | 2 | 1 | 37.3 | 1.62 |
| 25 | E9QDS5_DANRE | Ubiquitin-like modifier-activating enzyme 5 | uba5 | 3 | 1 | 44.5 | 0 |
| 26 | Q2TBJ1_DANRE | Zgc:123077 | zgc:123077 | 9 | 1 | 28.3 | 7.39 |
| 27 | B8JLK8_DANRE | Integrin, alpha V | itgav | 11 | 11 | 115 | 24.55 |
| 28 | X1WBF3_DANRE | Si:dkey-73p2.3 | si:dkey-73p2.3 | 1 | 1 | 220.4 | 1.85 |
| 29 | Q7SXT2_DANRE | Tetraspanin | tspan7 | 6 | 1 | 27.7 | 2.77 |
| 30 | A0A2R8PYV5_DANRE | TAR DNA-binding protein,-like | tardbpl | 9 | 2 | 34.5 | 5.62 |
| 31 | Q3YMK9_DANRE | Iron regulatory protein 1 | aco1 | 6 | 5 | 98.9 | 8.91 |
| 32 | A0A0G2L9K3_DANRE | Sorting nexin 2 | snx2 | 3 | 1 | 57.2 | 3.78 |
| 33 | A0A0R4IZE4_DANRE | Vasodilator-stimulated phosphoprotein a | vaspa | 5 | 1 | 41.9 | 2.4 |
| 34 | UBC9A_DANRE | SUMO-Conjugating enzyme UBC9-A (EC 2.3.2.-) (RING-type E3 SUMO transferase UBC9-A) (SUMO-protein ligase A) (Ubiquitin carrier protein 9-A) (Ubiquitin carrier protein I-A) (Ubiquitin-Conjugating enzyme E2 I-A) (Ubiquitin-protein ligase I-A) | ube2ia ubc9a ube2i | 6 | 1 | 18 | 1.72 |
| 35 | DECR2_DANRE | Peroxisomal 2,4-dienoyl-CoA reductase (EC 1.3.1.34) (2,4-dienoyl-CoA reductase 2) | decr2 si:ch211-153c20.5 zgc:85626 | 9 | 2 | 32 | 4.24 |
| 36 | A0A0R4IB74_DANRE | Si:dkey-225n22.4 | si:dkey-225n22.4 | 2 | 1 | 53.9 | 0 |
| 37 | F1QF91_DANRE | Integrin beta | itgb5 | 2 | 1 | 91.3 | 1.7 |
| 38 | EDC3_DANRE | Enhancer of mRNA-decapping protein 3 (LSM16 protein homolog) (YjeF domain-Containing protein 1) | edc3 lsm16 yjdc zgc:112006 | 2 | 1 | 54.8 | 0 |
| 39 | X1WGC3_DANRE | Cytochrome P450, family 8, subfamily B, polypeptide 3 (Fragment) | cyp8b3 | 2 | 1 | 59 | 2.41 |
| 40 | A0A0N4SU34_DANRE | Major histocompatibility complex class I ZAA | mhc1zaa | 8 | 3 | 38.7 | 5.76 |
| 41 | A0A286YBE9_DANRE | Plectin a | pleca | 4 | 17 | 543.8 | 43.16 |
| 42 | Q8AW60_DANRE | Isovaleryl Coenzyme A dehydrogenase (Isovaleryl-CoA dehydrogenase) (Novel protein similar to human isovaleryl Coenzyme A dehydrogenase (IVD)) | ivd OTTDARP00000001747 dZ181P14.1-001 | 11 | 5 | 45.8 | 8.46 |
| 43 | Q6P2M0_DANRE | Zgc:77235 | rpl27a zgc:77235 | 19 | 2 | 16.6 | 5.94 |
| 44 | Q0VFW0_DANRE | Ribosomal protein L22-like 1 (Zgc:136591) | rpl22l1 zgc:136591 | 10 | 1 | 14.8 | 3.83 |
| 45 | F1R0C0_DANRE | 6-phosphogluConolactonase (6PGL) (EC 3.1.1.31) | pgls | 3 | 1 | 26.9 | 1.91 |
| 46 | X1WBX3_DANRE | Formyl peptide receptor 1 (Fragment) | fpr1 | 3 | 1 | 44 | 2.2 |
| 47 | E7F2P0_DANRE | Syntaxin 2b | stx2b si:dkey-245g2.3 | 3 | 1 | 33.7 | 2.71 |
| 48 | A0A0R4ICE9_DANRE | NEDD8-activating enzyme E1 regulatory subunit | nae1 | 3 | 1 | 62.3 | 3.39 |
| 49 | A7MCK2_DANRE | Hsd17b12b protein (Hydroxysteroid (17-beta) dehydrogenase 12b) | hsd17b12b | 7 | 1 | 34.3 | 3.73 |
| 50 | B0S784_DANRE | Receptor expression-enhancing protein | zgc:101744 | 4 | 1 | 20.9 | 0 |
| 51 | Q502F2_DANRE | Si:dkey-90m5.4 protein (Fragment) | si:dkey-90m5.4 | 6 | 2 | 41.1 | 1.83 |
| 52 | E7F6T2_DANRE | GRAM domain-Containing 4b | gramd4b | 2 | 1 | 70.4 | 0 |
| 53 | E7F354_DANRE | 3-monooxygenase/tryptophan 5-monooxygenase activation protein, gamma polypeptide 2 | ywhag2 | 30 | 1 | 28.3 | 35.85 |
| 54 | A3KNW4_DANRE | S100v2 protein | s100v2 si:dkey-78d16.1 | 23 | 2 | 11.1 | 5.01 |
| 55 | E7FC32_DANRE | Tubulin beta chain | tubb1 si:ch211-122i22.4 | 11 | 2 | 52.5 | 18.63 |
| 56 | F1QVM4_DANRE | COMM domain-Containing 5 | commd5 | 4 | 1 | 23.6 | 0 |
| 57 | A0A0G2L2R7_DANRE | Methionine synthase (EC 2.1.1.13) (5-methyltetrahydrofolate--homocysteine methyltransferase) | mtr | 1 | 1 | 139.3 | 2.04 |
| 58 | F1R852_DANRE | Transmembrane 9 superfamily member | tm9sf1 | 1 | 1 | 68.7 | 1.94 |
| 59 | E7F1V3_DANRE | TBC1 domain-Containing kinase | tbck | 1 | 1 | 100.9 | 1.83 |
| 60 | A0A0R4ITY1_DANRE | Cdkn1a-interacting zinc finger protein 1a | ciz1a | 1 | 1 | 78 | 0 |
| 61 | A0A0R4IQJ3_DANRE | Fraser extracellular matrix complex subunit 1 | fras1 | 0 | 1 | 443.7 | 0 |
| 62 | X1WCB1_DANRE | BTB and CNC homology 1, basic leucine zipper transcription factor 2a | bach2a | 1 | 1 | 85.7 | 0 |
| 63 | A0A2R8Q9J9_DANRE | Regucalcin | rgn | 3 | 1 | 32.8 | 0 |
| 64 | A5PLC6_DANRE | BCL2-associated athanogene 1 (Si:dkey-216e9.4 protein) | bag1 si:dkey-216e9.4 | 6 | 1 | 22.6 | 1.63 |
| 65 | E9QF87_DANRE | Tpd52-like 2a | tpd52l2a | 5 | 1 | 21.8 | 2.35 |
| 66 | Q7ZVF2_DANRE | Glutamine synthetase (EC 6.3.1.2) | glulb | 5 | 2 | 41.9 | 2.3 |
| 67 | Q5SPA1_DANRE | Heme-binding protein 2 (SOUL1) | hebp2 soul1 | 8 | 1 | 21.5 | 2.75 |
| 68 | A1L1W6_DANRE | Zgc:158466 | trappc12 ttc15 zgc:158466 | 1 | 1 | 84.4 | 1.84 |
| 69 | Q6P6F0_DANRE | Ribosomal protein S17 (Zgc:77702) | rps17 zgc:77702 | 15 | 1 | 15.4 | 2.9 |
| 70 | A0A286YBI9_DANRE | Mannose receptor, C type 2 | mrc2 | 2 | 3 | 167.8 | 7.98 |
| 71 | A3KNI1_DANRE | Sfrs7 protein | srsf7b sfrs7 srsf7 | 4 | 1 | 23.7 | 2.18 |
| 72 | A1L1S0_DANRE | Leucine rich repeat Containing 15 | lrrc15 | 2 | 1 | 62.9 | 2.2 |
| 73 | Q7ZUZ4_DANRE | Programmed cell death 5 | pdcd5 | 10 | 1 | 14.6 | 2.38 |
| 74 | F1Q5J7_DANRE | Selenoprotein N | selenon sepn1 | 2 | 1 | 62.6 | 1.95 |
| 75 | Q8QGV4_DANRE | Lipocalin-type prostaglandin D synthase-like protein (Prostaglandin D synthase homolog) | ptgdsb.1 ptgds ptgdsb | 7 | 1 | 20.9 | 0 |
| 76 | Q6DGH7_DANRE | Proteasome (Prosome, macropain) assembly Chaperone 3 (Psmg3 protein) | psmg3 | 10 | 1 | 13.4 | 0 |
| 77 | Q31364_DANRE | Mhc-UAA (Fragment) | mhc1uaa UAA*01 | 10 | 1 | 26.4 | 6.55 |
| 78 | WDR81_DANRE | WD repeat-Containing protein 81 | wdr81 | 0 | 1 | 228.3 | 0 |
| 79 | A9JT41_DANRE | Rrbp1 protein | rrbp1b rrbp1 | 14 | 1 | 108.5 | 31.88 |
| 80 | A0A2R8Q4N6_DANRE | Uncharacterized protein | LOC569153 LOC100005266 | 24 | 7 | 29.4 | 13.73 |
| 81 | Q6NZW5_DANRE | Hsd17b4 protein | hsd17b4 | 1 | 1 | 78.6 | 1.92 |
| 82 | Q3B7R0_DANRE | Cytochrome c-1 | cyc1 | 7 | 2 | 33.7 | 0 |
| 83 | A7MC20_DANRE | LOC559122 protein (Fragment) | cfhl4 LOC559122 | 2 | 1 | 53.4 | 0 |
| 84 | F8W573_DANRE | Guanine nucleotide-binding protein (G protein), alpha 14 | gna14 | 3 | 1 | 41.3 | 0 |
| 85 | Q6NWC1_DANRE | Vdac1 protein (Voltage-dependent anion channel 1) | vdac1 VDAC1 | 3 | 1 | 30.6 | 0 |
| 86 | F1R369_DANRE | Reticulocalbin 1, EF-hand calcium-binding domain | rcn1 | 5 | 1 | 37.8 | 1.77 |
| 87 | Q6DHJ0_DANRE | Serine and arginine-rich-splicing factor 5b (Splicing factor, arginine/serine-rich 5b) | srsf5b sfrs5b | 6 | 1 | 32.2 | 4.9 |
| 88 | A0EXC6_DANRE | Sulfotransferase (EC 2.8.2.-) | sult2st3 | 13 | 1 | 33.4 | 8.64 |
| 89 | MRPB_DANRE | MARCKS-related protein 1-B (MARCKS-like protein 1-A) (MARCKS-like protein 1-B) | marcksl1b marcksl1a marcksl2 zgc:85717 | 8 | 1 | 21 | 2.93 |
| 90 | Q8JIZ0_DANRE | Envelope protein | env | 2 | 1 | 71.2 | 0 |
| 91 | B0R194_DANRE | DNA polymerase beta | polb | 4 | 1 | 38.9 | 0 |
| 92 | RECK_DANRE | Reversion-inducing cysteine-rich protein with Kazal motifs | reck | 1 | 1 | 105.5 | 0 |
| 93 | A3KP65_DANRE | Malonyl-CoA decarboxylase (Zgc:162977 protein) | mlycd zgc:162977 | 2 | 1 | 57.5 | 0 |
| 94 | E9QC44_DANRE | Phosphatidylinositol transfer protein cytoplasmic 1b | pitpnc1b | 18 | 1 | 6.7 | 1.71 |
| 95 | A2BE49_DANRE | Protein phosphatase, Mg2+/Mn2+-dependent, 1F | ppm1f | 2 | 1 | 46.2 | 1.64 |
| 96 | Q4QRG3_DANRE | Ubiquinol-cytochrome c reductase core protein II | uqcrc2b uqcrc2 | 5 | 2 | 48.1 | 5.48 |
| 97 | F1R2L8_DANRE | DEAD (Asp-Glu-Ala-Asp) box helicase 1 | ddx1 | 1 | 1 | 82.4 | 1.88 |
| 98 | A0A2R8RUA0_DANRE | Nucleobindin 2a | nucb2a | 2 | 1 | 59.5 | 0 |
| 99 | F1QV93_DANRE | Si:dkey-97a13.6 (Fragment) | si:dkey-97a13.6 | 1 | 1 | 250 | 0 |
| 100 | A0A2R8PX02_DANRE | Cytoplasmic FMR1-interacting protein | cyfip2 | 2 | 3 | 145.8 | 6.54 |
| 101 | B0R1C4_DANRE | Fibroblast activation protein, alpha | fap si:dkey-81j5.3 | 3 | 2 | 85.8 | 4.19 |
| 102 | B3DIL4_DANRE | Similar to Phosphatidylcholine transfer protein (PC-TP) (StAR-related lipid transfer protein 2) (StARD2) (START domain-Containing protein 2) | pctp LOC566726 | 10 | 2 | 24.9 | 3.76 |
| 103 | Q7ZU43_DANRE | Carboxypeptidase, vitellogenic-like | cpvl | 3 | 1 | 54.5 | 0 |
| 104 | Q4V8Z9_DANRE | Protein geranylgeranyltransferase type I, beta subunit | pggt1b | 2 | 1 | 39.9 | 1.98 |
| 105 | A0A2R8PW45_DANRE | Alpha-1-microglobulin/bikunin precursor | ambp | 4 | 1 | 38.9 | 2.08 |
| 106 | H0WES8_DANRE | ATP synthase membrane subunit DAPIT | atp5md si:dkeyp-80c12.10 | 17 | 1 | 6.3 | 1.72 |
| 107 | F1RBH2_DANRE | ADP-ribosylation factor-like 8Bb | arl8bb | 5 | 1 | 21.5 | 2.1 |
| 108 | Q7T314_DANRE | translocase of Outer mitochondrial membrane 40 homolog (Yeast) | tomm40 | 3 | 1 | 36.2 | 1.88 |
| 109 | A0A0R4IIP1_DANRE | tRNA-dihydrouridine(47) synthase [NAD(P)(+)] (EC 1.3.1.-) (tRNA-dihydrouridine synthase 3) | dus3l | 2 | 1 | 75.4 | 0 |
| 110 | A0A0R4IEP9_DANRE | TRAF2 and NCK-interacting kinase a | tnika | 1 | 1 | 151.3 | 0 |
| 111 | CISD2_DANRE | CDGSH iron-sulfur domain-Containing protein 2 | cisd2 dkey-162b23.1 zgc:64148 | 8 | 1 | 15.5 | 1.82 |
| 112 | A0A2R8QF89_DANRE | Protein farnesyltransferase subunit beta (FTase-beta) (EC 2.5.1.58) | fntb | 4 | 2 | 49.9 | 1.62 |
| 113 | H0WEY0_DANRE | Si:dkey-84h14.1 | si:dkey-84h14.1 | 5 | 1 | 120 | 11.09 |
| 114 | Q4V8Q4_DANRE | Golgi transport 1Bb (Zgc:114204) | golt1bb golt1b zgc:114204 | 7 | 1 | 15.1 | 1.7 |
| 115 | A4U7F9_DANRE | Procollagen lysine 2-oxoglutarate 5-dioxygenase 2b isoform (EC 1.14.11.4) (Procollagen-lysine, 2-oxoglutarate 5-dioxygenase 2) | plod2 | 2 | 1 | 87.5 | 3.66 |
| 116 | E7F8S2_DANRE | Kinesin-like protein (Fragment) | kif5ab si:ch73-130n22.1 | 4 | 3 | 114.1 | 10.21 |
| 117 | F1QHZ3_DANRE | Tetratricopeptide repeat domain 4 | ttc4 | 4 | 2 | 46 | 0 |
| 118 | F1R9W6_DANRE | Zgc:110239 | zgc:110239 | 3 | 1 | 61.4 | 1.64 |
| 119 | A4JYL6_DANRE | Sid4 | sid4 | 3 | 1 | 40.1 | 0 |
| 120 | A2BIR0_DANRE | SEC14-like lipid-binding 8 | sec14l8 si:ch211-89f7.1 | 2 | 1 | 45.3 | 2.65 |
| 121 | F1QPT3_DANRE | GDP-mannose 4,6-dehydratase | gmds | 19 | 7 | 42.4 | 18.58 |
| 122 | B2GQM8_DANRE | Yipf3 protein | yipf3 | 3 | 1 | 37.8 | 0 |
| 123 | F1QHM3_DANRE | Transglutaminase 1-like 3 | tgm1l3 | 1 | 1 | 70.6 | 0 |
| 124 | F1QM43_DANRE | Guanosine-3',5'-bis(diphosphate) 3'-pyrophosphohydrolase MESH1 | hddc3 | 4 | 1 | 20.4 | 1.81 |
| 125 | F1Q8J2_DANRE | Ubiquitin protein ligase E3 component n-recognin 5 | ubr5 | 1 | 1 | 308.3 | 0 |
| 126 | TM258_DANRE | Transmembrane protein 258 (Dolichyl-diphosphooligosaccharide--protein glycosyltransferase subunit TMEM258) (Oligosaccharyl transferase subunit TMEM258) | tmem258 zgc:73269 | 10 | 1 | 9.1 | 1.68 |
| 127 | A9C3Q0_DANRE | Collagen beta(1-O)galactosyltransferase 2 | colgalt2 glt25d2 | 1 | 1 | 70.6 | 0 |
| 128 | A0A0R4IRS1_DANRE | Adaptor-related protein complex 1 subunit mu 1 | ap1m1 | 3 | 1 | 48.5 | 1.86 |
| 129 | IF6_DANRE | Eukaryotic translation initiation factor 6 (eIF-6) | eif6 si:ch211-239j9.6 | 8 | 2 | 26.6 | 4.21 |
| 130 | E7FDD7_DANRE | RNA polymerase II subunit E | polr2ea | 8 | 2 | 24.7 | 1.98 |
| 131 | F1QLU1_DANRE | Si:ch211-149a19.3 | si:ch211-149a19.3 si:ch211-149a19.4 | 2 | 1 | 152.1 | 3.76 |
| 132 | B0UXH8_DANRE | Tight junction protein 2b (zona occludens 2) | tjp2b | 2 | 1 | 132.1 | 5.41 |
| 133 | F1QTY6_DANRE | Acylaminoacyl-peptide hydrolase | apeh | 5 | 3 | 83.2 | 7.8 |
| 134 | Q6PFN9_DANRE | Glycine dehydrogenase (Decarboxylating) | gldc | 1 | 1 | 109.2 | 2.5 |
| 135 | Q6PBJ6_DANRE | NADH:ubiquinone oxidoreductase subunit B (Zgc:73375) | ndufb6 zgc:73375 | 10 | 2 | 15 | 0 |
| 136 | Q6P0J6_DANRE | Zgc:77221 | thumpd1 zgc:77221 | 3 | 1 | 39.2 | 1.71 |
| 137 | F1R676_DANRE | Protein O-fucosyltransferase 1 | pofut1 | 3 | 1 | 45.1 | 2.37 |

**Supplementary Table 8.** Primer sequences of related genes.

| **Gene** | **Primer sequence** | **Use** |
| --- | --- | --- |
| *ogt* -F | AGCAGCAACAGGAGAAGAAG | qRT-PCR |
| *ogt*-R | GGGTTGGAGGGTCAATCTTATAG |  |
| *oga-*F | AGAGGATTCAGGCAGTGATATTG |  |
| *oga-*R | CAGAGTAAGTGCCTCGGTAATG |  |
| β-actin-F | GACTGAGCGCGGTTATTCTT |  |
| β-actin-R | CTCAAAGTCCAGAGCCACATAG |  |
| *ogt*-F | AGAAGCCCTCATGCACTATAAG | PCR |
| *ogt*-R | GGCTGAGAGCATAGCAGAAA |  |
| *oga*-F | TCTTCATTACCGAGGCACTTAC |  |
| *oga*-R | GTCAGGAAACCTCCCACTAAC |  |

**Supplementary Table 9 O-GlcNAc glycoprotein involved in regeneration-related biological processes**

| **No.** | **Protein Acc No.** | **Protein Name** | **Gene Name** | **KO** |
| --- | --- | --- | --- | --- |
| 1 | A0A0R4IS70_DANRE | Purine-rich element-binding protein Ba | purba | K21799 |
| 2 | Q0ZBR7_DANRE | Macrophage migration inhibitory factor | mif MIF | K07253 |
| 3 | F1QV31_DANRE | Keratin 5 | krt5 | K07605 |
| 4 | Q6DHB6_DANRE | Keratin 91 (Zgc:92533) | krt91 krt17 zgc:92533 | K07604 |
| 5 | Q7T3A8_DANRE | Annexin | anxa1a |  |
| 6 | Q803B0_DANRE | Heat shock 60 protein 1 | hspd1 | K04077 |
| 7 | F1Q890_DANRE | Plasminogen | plg | K01315 |
| 8 | A0A2R8QR17_DANRE | Plectin b | plecb |  |
| 9 | E7F8G7_DANRE | Plectin a | pleca |  |
| 10 | A0A0R4IAS2_DANRE | Periplakin | ppl |  |
| 11 | F1Q4X8_DANRE | Envoplakin a | evpla | K10383 |
| 12 | F8W4Q1_DANRE | Desmoplakin a | dspa |  |
| 13 | I3ISA6_DANRE | Epiplakin 1 | eppk1 |  |
| 14 | A0A2R8QH31_DANRE | Smoothelin,-like | smtnl |  |
| 15 | A7MCP4_DANRE | Ras homolog gene family, member Ab | rhoab |  |
| 16 | Q6NZV4_DANRE | Cysteine and glycine-rich protein 1 | csrp1a csrp1 | K09377 |
| 17 | Q8JHE0_DANRE | Casein kinase 1, alpha 1 | csnk1a1 |  |
| 18 | A0A0R4IL09_DANRE | AP-2 complex subunit mu-A | ap2m1a |  |
| 19 | Q8AWD4_DANRE | Tax1-binding protein 3 | tax1bp3 |  |
| 20 | A0A2R8QIW0_DANRE | Histone deacetylase (EC 3.5.1.98) | hdac1 |  |
| 21 | A0A2R8QN11_DANRE | Fermitin family homolog 2 | fermt2 |  |
| 22 | E7F2R9_DANRE | Protein tyrosine kinase 7a | ptk7a |  |
| 23 | F1QFC0_DANRE | Heat shock protein 9 | hspa9 |  |
| 24 | Q4JHL7_DANRE | Voltage-dependent anion channel 2 | vdac2 |  |
| 25 | Q1LXE1_DANRE | Hyaluronan and proteoglycan link protein 1a | hapln1a | K06848 |
| 26 | Q8JGN2_DANRE | Fas-associated factor 1 | faf1 |  |
| 27 | Q1LYP4_DANRE | Chromodomain helicase DNA-binding protein 4a | chd4a si:ch211-51m24.3 | K11643 |
| 28 | B3DKM0_DANRE | Aldehyde dehydrogenase 1 family, member A2 | aldh1a2 |  |
| 29 | Q7ZSY3_DANRE | Brahma protein-like protein 1 dependent regulator of chromatin, subfamily a, member 4a | smarca4a brg1 smarca4 | K11647 |
| 30 | X1WE42_DANRE | Nidogen 2a (osteonidogen) | nid2a |  |
| 31 | F1QCE3_DANRE | Peroxiredoxin 5 | prdx5 |  |
| 32 | A0A2R8QIW0_DANRE | Histone deacetylase | hdac1 |  |
| 33 | F1QLG2_DANRE | Metastasis-associated 1 family, member 2 | mta2 |  |
| 34 | F1R2E0_DANRE | Heterogeneous nuclear ribonucleoprotein M | hnrnpm |  |
| 35 | B0S754_DANRE | Small nuclear ribonucleoprotein 200 (U5) | snrnp200 si:ch211-251j10.5 | K12854 |
| 36 | Q6DRE8_DANRE | Small nuclear ribonucleoprotein 70 | snrnp70 snrp70 | K11093 |
| 37 | SF3B3_DANRE | Splicing factor 3B subunit 3 | sf3b3 zgc:55440 | K12830 |
| 38 | A8WFS1_DANRE | Eftud2 protein | eftud2 | K12852 |
| 39 | A0A0R4J8E6_DANRE | Pre-mRNA-processing factor 8 | prpf8 | K12856 |
| 40 | F1QKC0_DANRE | SMU1, DNA replication regulator and spliceosomal factor a | smu1a smu1 |  |
| 41 | B1H1M2_DANRE | Zgc:63611 protein | aqr zgc:63611 | K12874 |
| 42 | Q7ZV92_DANRE | PRP19/PSO4 homolog (S. cerevisiae) | prpf19 prp19 | K10599 |
| 43 | PRP39_DANRE | Pre-mRNA-processing factor 39 | prpf39 si:dz261o22.3 | K13217 |
| 44 | Q7ZUE4_DANRE | PRP40 pre-mRNA processing factor 40 homolog A (Yeast) | prpf40a | K12821 |
| 45 | Q6DG11_DANRE | Spliceosome-associated factor 1, recruiter of U4/U6.U5 tri-snRNP | sart1 | K11984 |
| 46 | F1R300_DANRE | Small nuclear ribonucleoprotein polypeptide A' | snrpa1 | K11092 |
| 47 | Q6YMR2_DANRE | MCG1346-like protein (PHD finger protein 5A) | phf5a | K12834 |
| 48 | Q6PBZ6_DANRE | Snrpb2 protein (Fragment) | snrpb2 |  |
| 49 | F1R300_DANRE | Small nuclear ribonucleoprotein polypeptide A' | snrpa1 | K11092 |
| 50 | A3KMS1_DANRE | Ddx46 protein (Fragment) | ddx46 |  |
| 51 | Q1LY92_DANRE | Similar to Xab2 protein | xab2 LOC555321 | K12867 |
| 52 | A7YT51_DANRE | Splicing factor 3b, subunit 2 | sf3b2 zgc:136773 | K12829 |
